# Supplementary material for: Topobexin targets the Topoisomerase II ATPase domain for beta isoform-selective inhibition and anthracycline cardioprotection
Source: Nat Commun. 2025 May 28;16:4928. doi: 10.1038/s41467-025-60167-9 (PMC12116762; doi:10.1038/s41467-025-60167-9)
Supplement: Supplementary file 1 — Supplementary Information [file 41467_2025_60167_MOESM1_ESM.pdf]

Supplementary Information for

Topobexin Targets the Topoisomerase II ATPase Domain for Beta Isoform-Selective Inhibition and Anthracycline Cardioprotection

Authors: Jan Kubeš[1]*, Galina Karabanovich[2]*, Anh T.Q. Cong[3,4]*, Iuliia Melnikova[2], Olga Lenčová[5], Petra Kollárová[5], Hana Bavlovič Piskáčková[6], Veronika Keresteš[1], Lenka Applová[1], Lise C.M. Arrouye[3], Julia R. Alvey[3], Jasmina Paluncic[3], Taylor L. Witter[3,4], Anna Jirkovská[1], Jiří Kuneš[2], Petra Štěrbová-Kovaříková[6], Caroline A. Austin[7]†, Martin Štěrba[5]†, Tomáš Šimůnek[1]†, Jaroslav Roh[2]†, and Matthew J. Schellenberg[3]†

*Contributed equally

†Correspondence and requests for materials should be addressed to: schellenberg.matthew@mayo.edu, rohj@faf.cuni.cz, simunekt@faf.cuni.cz, sterbam@lfhk.cuni.cz or caroline.austin@newcastle.ac.uk

The PDF file includes:

Supplementary Figs. 1 to 8

Supplementary Tables 1 to 5

Supplementary Methods – Chemistry (Synthetic procedures and characterization of compounds)

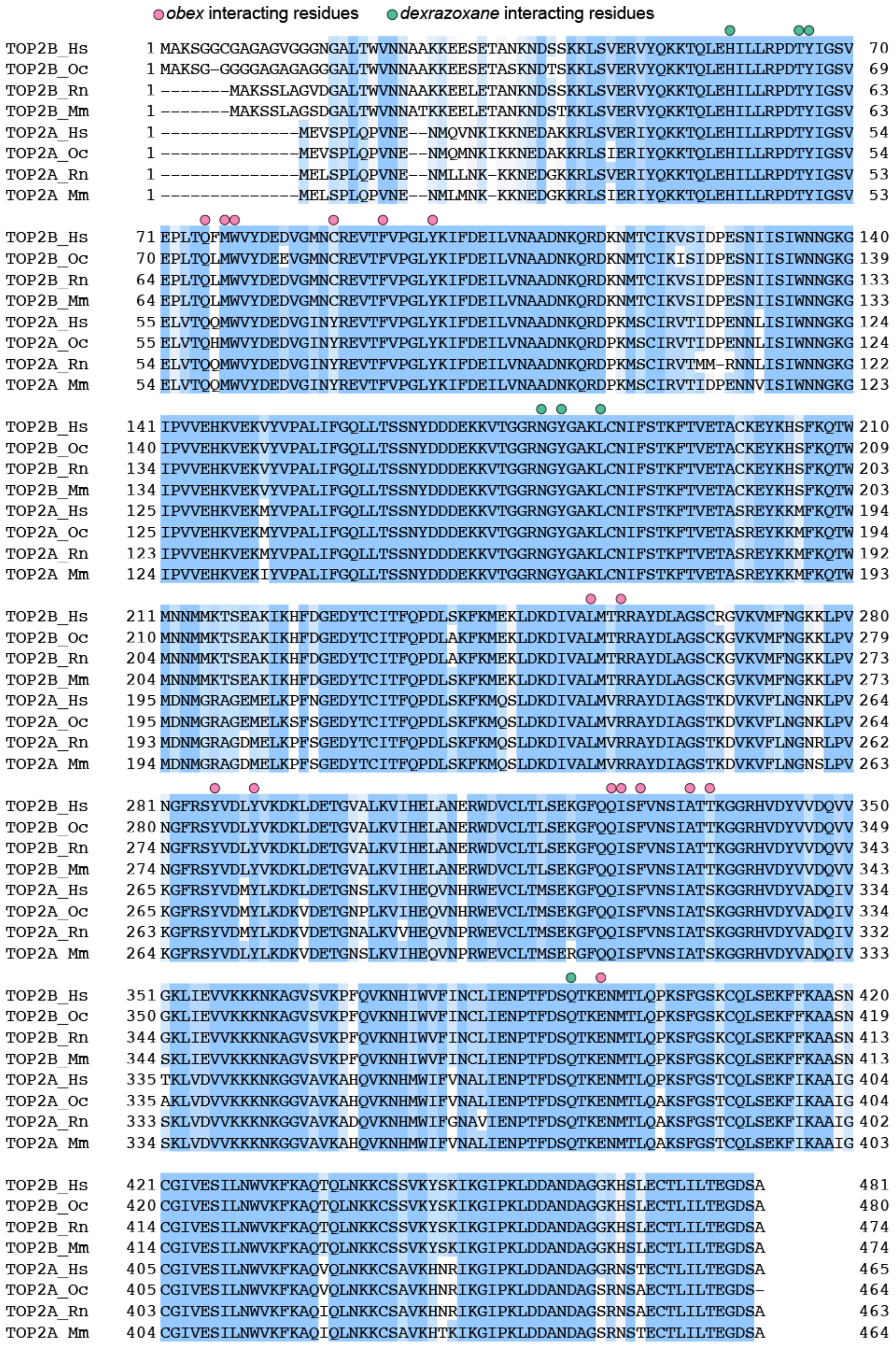

Supplementary Fig. 1. Sequence alignment of TOP2A and TOP2B ATPase domains from species used in this study. Hs, Homo sapiens; Oc, Oryctolagus cuniculus; Rn Rattus norvegicus; Ms, Mus musculus. Residues that comprise the obex (pink) and dexrazoxane (teal) binding sites are indicated.

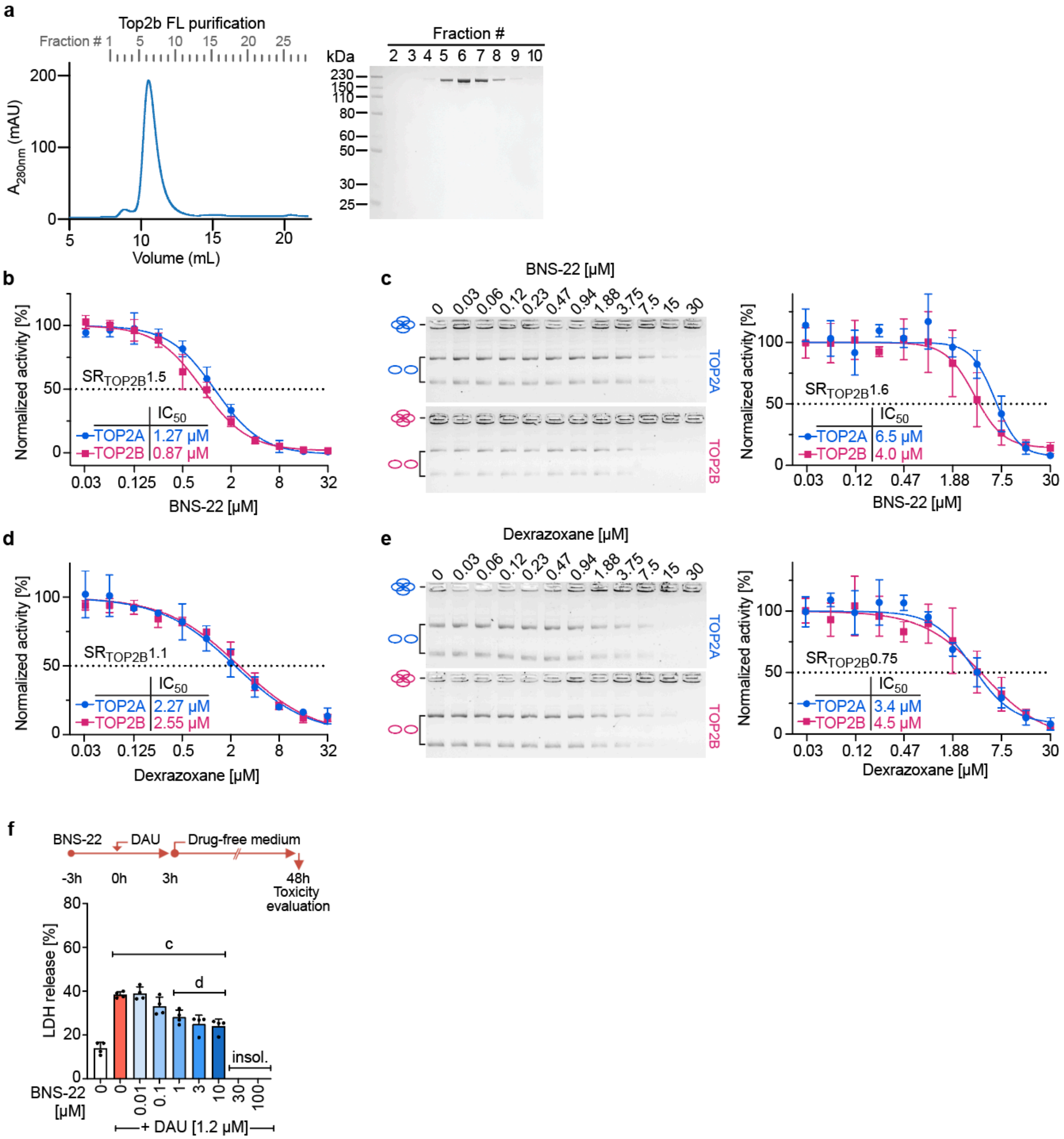

Supplementary Fig. 2. a, Final size-exclusion purification of recombinant TOP2B from YFP-tag HEK293F cell expression system. **b,** Inhibition of ATPase activity of recombinant human TOP2A and TOP2B by BNS22 (n=3, mean ± SD normalized to untreated control). **c,** Inhibition of decatenation activity of recombinant human TOP2A and TOP2B by BNS22 (n=3, mean ± SD normalized to untreated control). Representative agarose gel is shown (right). **d,** Inhibition of ATPase activity of recombinant human TOP2A and TOP2B by dexrazoxane (n=3, mean ± SD normalized to untreated control). **e,** Inhibition of decatenation activity of recombinant human TOP2A and TOP2B by dexrazoxane (n=3, mean ± SD normalized to untreated control). Representative agarose gel is shown (right). **f,** Protective effects of BNS-22 against toxicity (LDH release) induced by DAU [1.2 µM] in isolated rat neonatal ventricular cardiomyocytes (NVCM) 48 h after DAU addition, n = 4, mean ± SD. Statistical significance ($P \leq 0.05$, one-way ANOVA) against untreated cells in column 1 is indicated as (c) or DAU treated cells in column 2 indicated as (d).

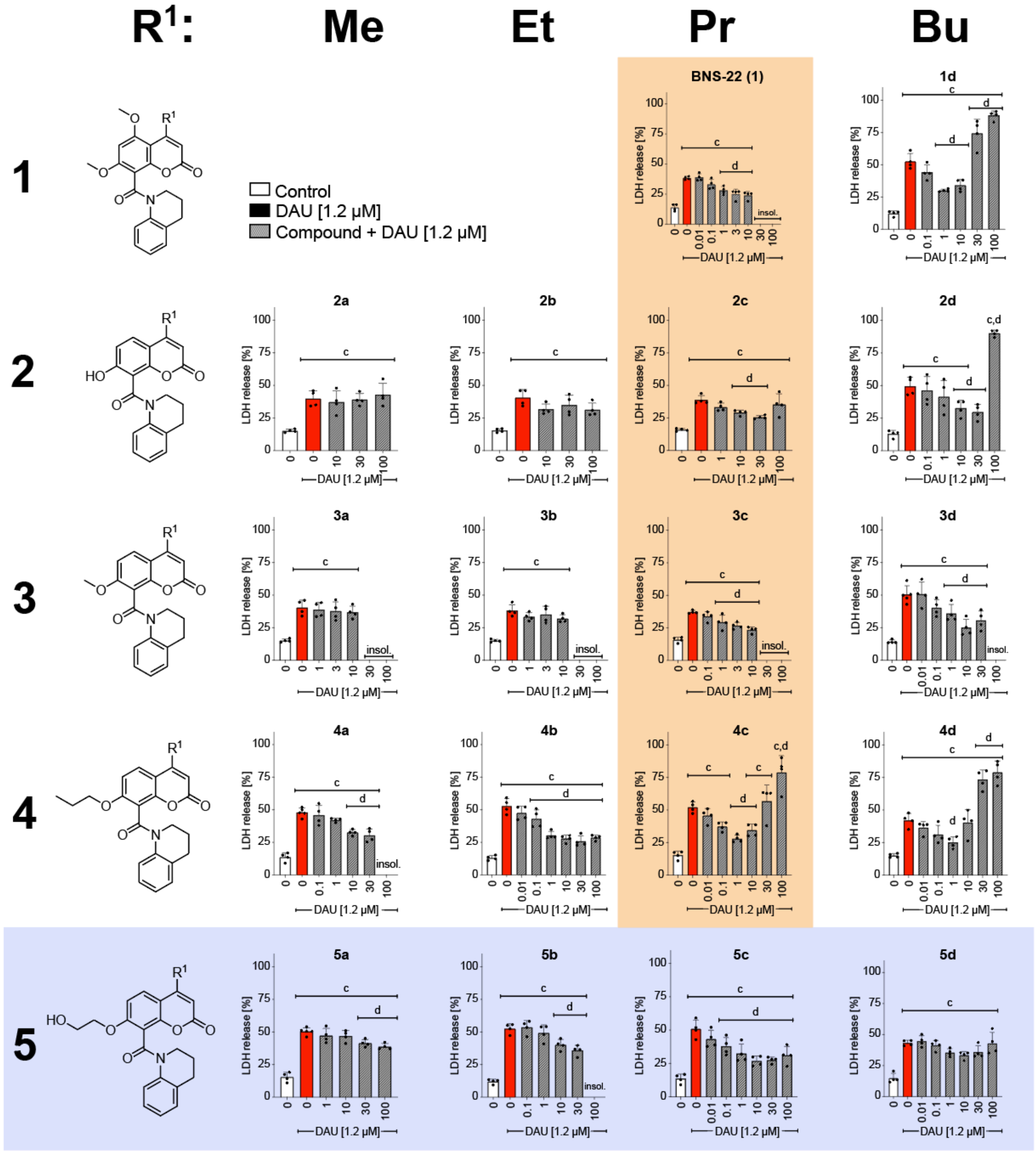

Supplementary Fig. 3. Protective effects of the first series of the novel compounds against DAU-induced cytotoxicity in primary cultures of rat cardiomyocytes. Cells were pre-treated with the indicated compound (or vehicle for controls; DMSO 0.1 % final concentration) for 3 h, another 3 h co-incubated with DAU (1.2 μM) and then left in drug-free media until evaluation by LDH release, which took place 48 h after DAU, n = 4, mean ± SD. Statistical significance ($P \leq 0.05$, one-way ANOVA) against untreated cells in column 1 is indicated as (c) or DAU treated cells in column 2 indicated as (d). Compounds in colored rectangles were selected for more detailed investigation as representatives of two axes of structural modifications.

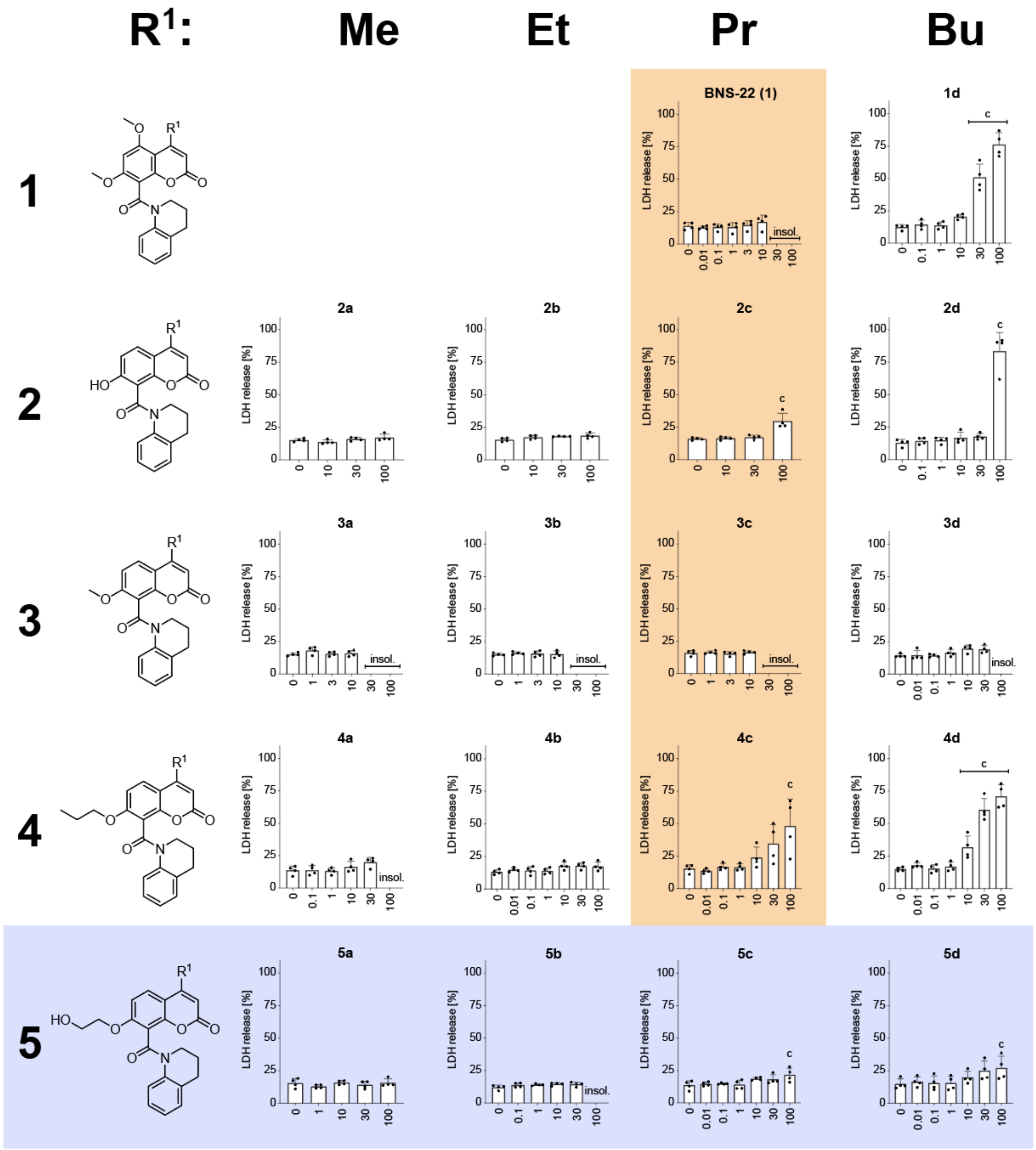

Supplementary Fig. 4. Inherent toxicities of the first series of the novel compounds in primary cultures of rat cardiomyocytes in settings corresponding to the cardioprotection model experiments. Cells were treated with the indicated compound (or vehicle for controls; DMSO 0.1 % final concentration) for 6 h, and then left in drug-free media for 45 h. Cellular toxicity was evaluated by LDH release, n = 4, mean ± SD. Statistical significance ($P \leq 0.05$, one-way ANOVA) against untreated cells in column 1 indicated as (c). Compounds in colored rectangles were selected for more detailed investigation as representatives of two axes of structural modifications.

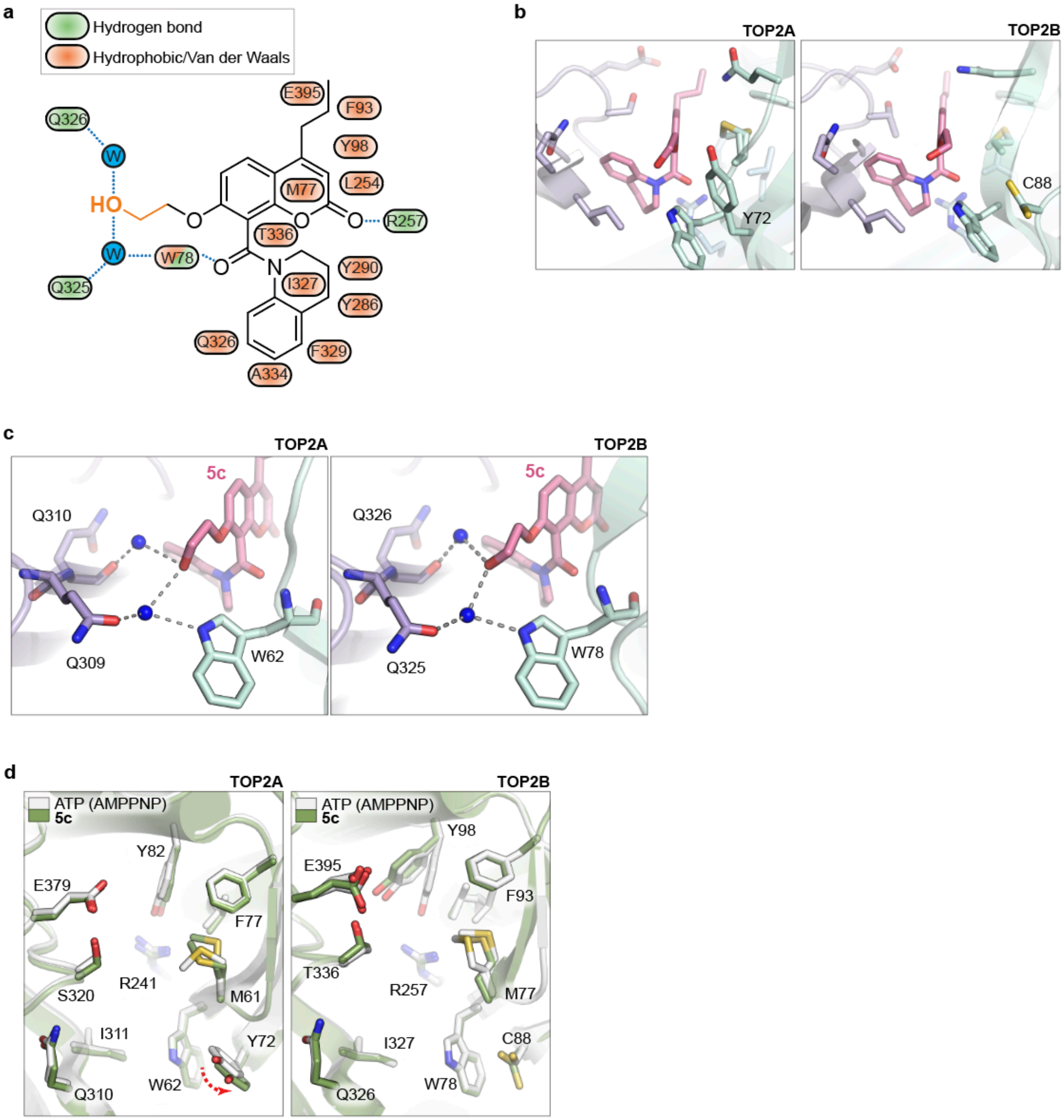

Supplementary Fig. 5. a, Diagram depicting residues of TOP2B that interact with compound **5c**. Residues are colored coded according to the type of interaction (hydrogen bonding or hydrophobic/Van der Waals). **b,** The position 7 hydroxyl group of compound **5c** extends towards the non-conserved Y72/C88 residue. **c,** The position 7 hydroxyl group of **5c** forms hydrogen bonds with ordered water molecules in both TOP2A and TOP2B. **d,** Alignment of the structures of TOP2A and TOP2B with or without **5c** showing similarities between the bound and unbound states.

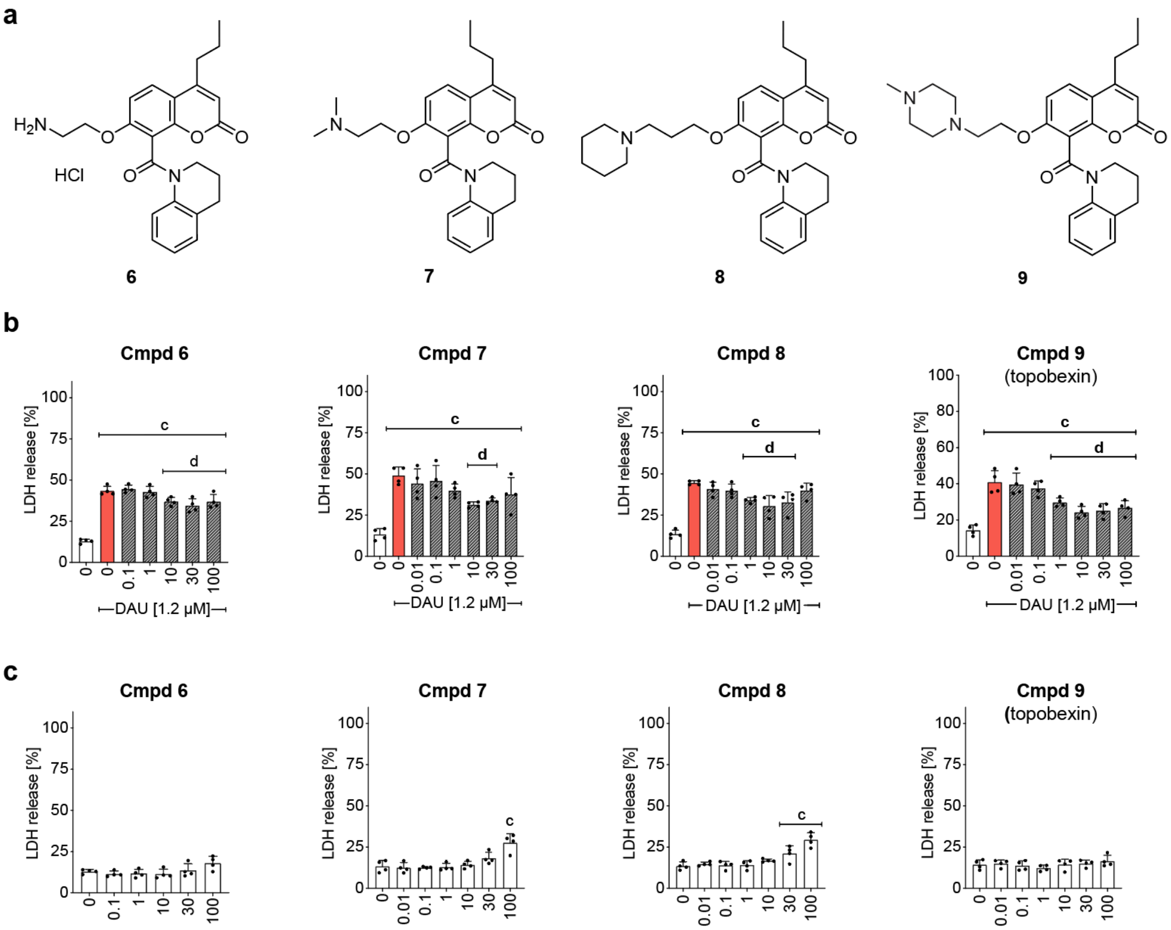

Supplementary Fig. 6. a, Chemical structures of analogues containing nitrogen in a side chain in position 7. **b,** Protective effects against DAU-induced cytotoxicity and **c,** corresponding inherent toxicities of derivatives based on **5c** designed for improved solubility and administrability. Cellular toxicity was evaluated by LDH release, n = 4, mean ± SD. Statistical significance ($P \leq 0.05$, one-way ANOVA) against untreated cells indicated as (c) or DAU treated cells indicated as (d). Data shown in panel B and C for topobexin (**9**) is reproduced from Fig. 4B.

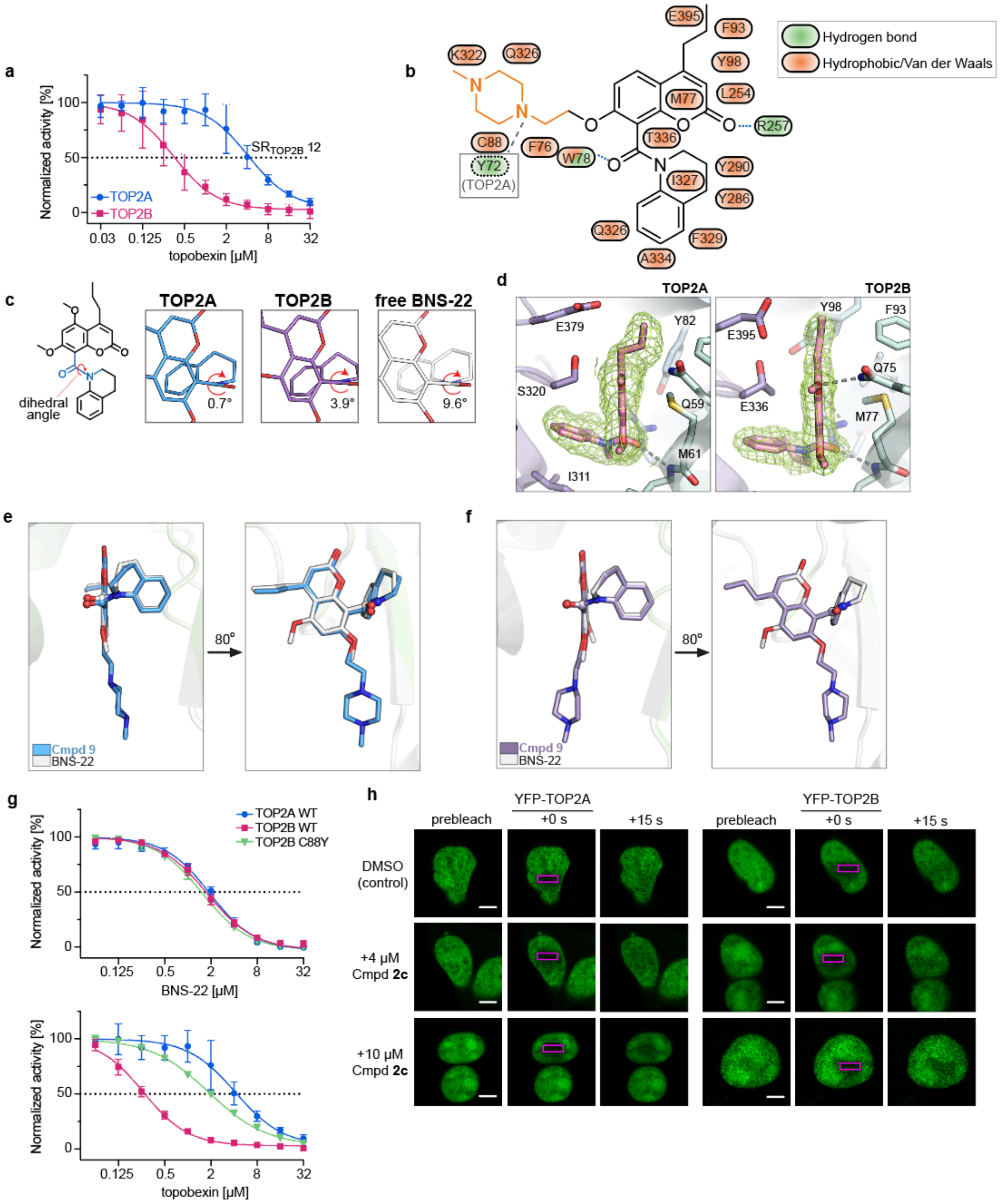

Supplementary Fig. 7. a, Inhibition of ATPase activity of recombinant human TOP2A and TOP2B by topobexin (**9**) (n=3, mean ± SD normalized to untreated control). **b,** Diagram depicting residues of TOP2B that interact with topobexin (**9**). Residues are colored coded according to the type of interaction (hydrogen bonding or hydrophobic/Van der Waals). **c,** Details of the amide dihedral angle of BNS-22 when bound to TOP2A, TOP2B or in unbound (free) state. **d,** Molecular architecture of the obex pocket binding BNS-22 in TOP2A and TOP2B. Electron density corresponding to BNS-22 from a composite omit map (green mesh, contoured at 1σ) reveals the location and conformation of BNS-22 (pink) within this pocket. **e,** Overlay of BNS22 and topobexin (**9**) bound to TOP2A reveals differences in the conformation of the tetrahydroquinoline ring. **f,** Overlay of BNS-22 and topobexin (**9**) bound to TOP2B reveals that the tetrahydroquinoline ring remains unchanged in topobexin (**9**). **g,** Inhibition of ATPase of TOP2B WT and C88Y mutants by BNS-22 (upper) and topobexin (**9**) (lower). TOP2A data from experiments described in Supplementary Data Fig. 7a is also shown for

comparison. The IC_{50} values of TOP2B C88Y mutant were 1.55 µM for BNS-22 and 1.88 µM for topobexin (**9**). **h,** Fluorescence Recovery After Photobleaching (FRAP) analysis. HEK293F cells that express yellow-fluorescent protein (YFP)-TOP2A or YFP-TOP2B were pre-incubated with or without compound **2c** for 15 min prior to FRAP analysis. YFP-TOP2 in the bleached area (purple box) rapidly mixes throughout the nucleus and the bleached area is no longer visible after 20 seconds in untreated cells. When inhibited by compound **2c** the bleached area is still visible after 20 seconds for both YFP-TOP2B and YFP-TOP2A.

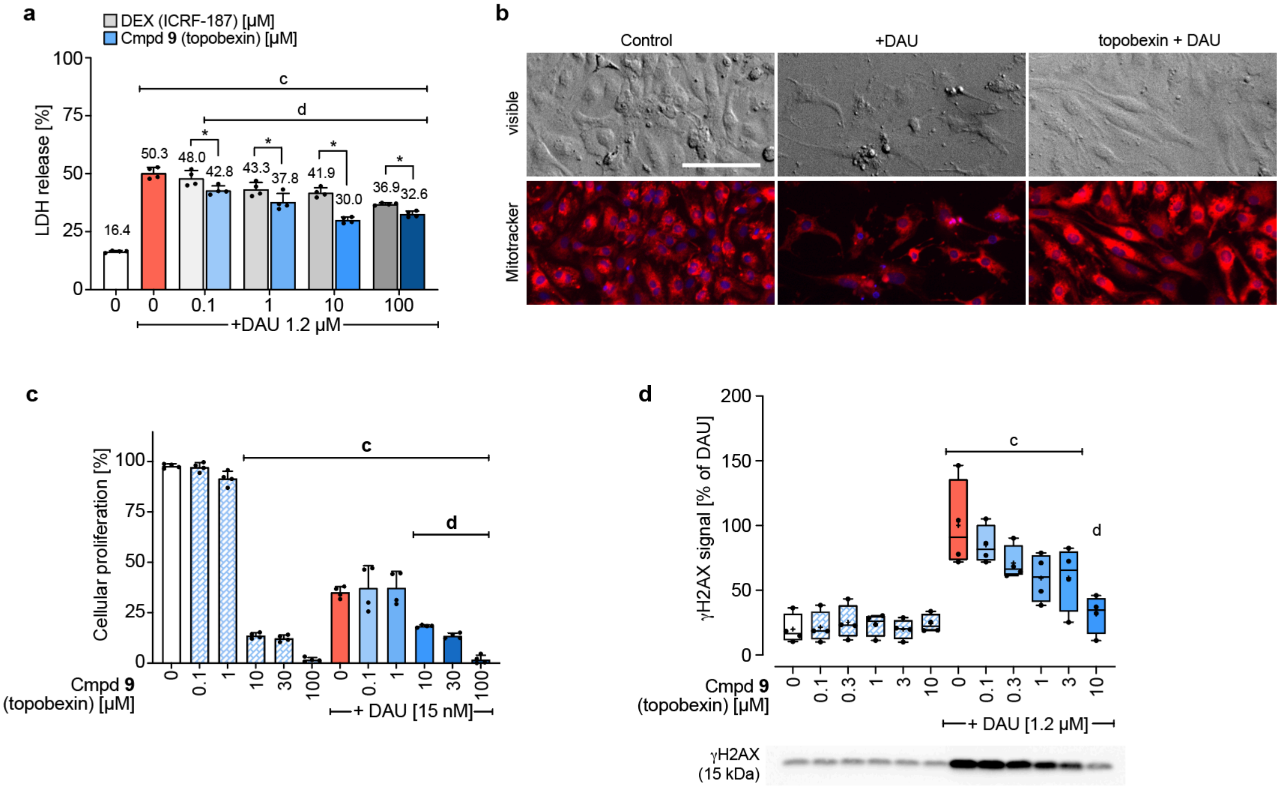

Supplementary Fig. 8. a, Side-by-side comparison of protective effects of dexrazoxane (ICRF-187) and topobexin (**9**) in isolated rat neonatal ventricular cardiomyocytes (NVCM) against toxicity (LDH release) induced by DAU [1.2 µM] 48 h after DAU addition, n = 4, mean ± SD. Statistical significance ($P \leq 0.05$): one-way ANOVA against untreated cells in column 1 indicated as (c) or against DAU treated cells in column 2 indicated as (d), or pairwise comparison in same concentrations by two-tailed ratio paired t-test indicated as (*). **b,** Live-cell imaging of NVCM cells in the schedule corresponding to the cytotoxicity/protection experiments (48 h after DAU addition). Changes in morphology are shown in visible light microscopy, red is fluorescence from MitoTracker Red CMXRos corresponding to active mitochondria, and blue signal is from staining nuclear DNA with Hoechst 33342. Scale bar = 100 µm. **c,** Antiproliferative activity of topobexin (**9**) and its effects on antiproliferative activity of DAU were examined on HL-60 promyelocytic leukemia cell line. Cells were incubated for 72 h in the presence of 0.1–100 µM topobexin (**9**), either alone or in combination with DAU in concentration corresponding to its previously determined IC_{50} (15 nM), n = 4, mean ± SD. Statistical significance ($P \leq 0.05$, one-way ANOVA) against untreated cells in column 1 indicated as (c) or against DAU treated cells in column 7 indicated as (d). **d,** The levels of phosphorylated γH2AX in HL-60 promyelocytic leukemia cell line evaluated by Western blotting (n = 4, $P \leq 0.05$ against untreated control cells (c) or DAU (d) (one-way ANOVA)). For all box and whisker plots, center line represents the median, "+" represents the mean. Bounds of box indicate 25th to 75th percentile, whiskers indicate minimal and maximal value.

Supplementary Table 1. Inhibitory concentrations and biochemical parameters of selected *obex* inhibitors. LEC = lowest effective concentration, LTC = lowest toxic concentration, n.d. = not determined (not cardioprotective at any concentration used).

	NVCM (n=4)		TOP2 ATPase activity (n=3)					Decatenation Activity of TOP2 Isoforms Expressed in Human Cells (HEK293F) (n=3)				
			SR_{beta}	TOP2A		TOP2B		SR_{beta}	TOP2A		TOP2B	
compound	LEC	LTC		IC_{50}	95% CI	IC_{50}	95% CI		IC_{50}	95% CI	IC_{50}	95% CI
BNS-22	1	> 10	1.5	1.27	(1.11–1.46)	0.87	(0.76–1.00)	1.6	6.51	(5.35–8.16)	4.00	(3.09–5.46)
dexrazoxane			0.89	2.27	(1.91–2.70)	2.55	(2.14–3.02)	0.75	3.35	(2.40-5.16)	4.50	(2.95-10.93)
**1d**	1	30										
**2a**	n.d.	> 100										
**2b**	n.d.	> 100										
**2c**	10	100	1.0	0.69	(0.62–0.77)	0.66	(0.60–0.74)					
**2d**	10	100										
**3a**	n.d.	> 10										
**3b**	n.d.	> 10										
**3c**	1	> 10	1.6	1.27	(1.15–1.40)	0.78	(0.70–0.86)					
**3d**	1	> 30										
**4a**	10	> 30										
**4b**	0.1	> 100										
**4c**	0.1	100	2.2	1.87	(1.25–2.72)	0.85	(0.66–1.13)					
**4d**	1	10										
**5a**	30	> 100										
**5b**	10	> 30	1.0	1.42	(0.90–2.50)	1.46	(0.88–2.79)					
**5c**	0.1	100	3.8	1.54	(1.45–1.64)	0.41	(0.39–0.43)	2.2	3.21	(2.52–4.19)	1.43	(1.23–1.67)
**5d**	n.d.	100										
**6**	10	> 100										
**7**	10	100										
**8**	1	30										
topobexin (**9**)	1	> 100	12	4.09	(2.89–6.29)	0.35	(0.27–0.46)	25	4.80	(4.08–5.71)	0.19	(0.16–0.23)

Supplementary Table 2. Macromolecular X-ray crystallography data collection and refinement statistics.

ATPase Protein	TOP2A	TOP2A	TOP2A	TOP2A	TOP2B	TOP2B	TOP2B	TOP2B
Compound	-	BNS-22	**5c**	Topobexin (**9**)	-	BNS-22	**5c**	Topobexin (**9**)
PDB entry ID	9BQ6	9BQ7	9BQ9	9BQB	9BQ8	9BQA	9BQC	9BQD
**Data collection**								
Space group	$P2_12_12_1$	$P2_12_12_1$	$P2_12_12_1$	$P2_12_12_1$	$P4_12_12$	$P4_12_12$	$P4_12_12$	$P4_12_12$
Cell dimensions								
a, b, c (Å)	69.78, 92.70, 125.46	69.93, 92.85, 126.95	70.10, 92.62, 126.90	69.80, 92.54, 125.58	83.76, 83.76, 127.1	84.00, 84.00, 126.69	84.15, 84.15, 127.75	83.94, 83.94, 127.12
α, β, γ (°)	90, 90, 90	90, 90, 90	90, 90, 90	90, 90, 90	90, 90, 90	90, 90, 90	90, 90, 90	90, 90, 90
Resolution (Å)	50-1.90 (1.97-1.90)	50-2.05 (2.12-2.05)	50-1.95 (2.02-1.95)	50-1.50 (1.55-1.50)	50-1.25 (1.29-1.25)	50.00-1.90 (1.97-1.90)	50-1.45 (1.50-1.45)	50-1.50 (1.55-1.50)
R_{merge}	0.095 (1.40)	0.118 (2.04)	0.118 (1.74)	0.072 (1.54)	0.081 (2.13)	0.118 (1.78)	0.047 (1.23)	0.078 (2.22)
$I / \sigma I$	18.7 (1.6)	19.4 (1.2)	16.4 (1.1)	32.5 (1.85)	34.8 (1.3)	23.5 (1.7)	36.6 (1.6)	34.3 (1.25)
$CC_{1/2}$	0.99 (0.53)	1.00 (0.50)	1.00 (0.49)	1.00 (0.61)	1.00 (0.49)	0.99 (0.60)	1.00 (0.57)	0.99 (0.43)
Completeness (%)	99.9 (99.6)	100 (100)	99.3 (99.7)	99.9 (100)	100 (99.9)	99.9 (99.9)	99.9 (99.9)	100 (100)
Redundancy	6.8 (6.8)	6.8 (7.0)	6.8 (6.8)	13.2 (12.9)	13.2 (12.3)	12.7 (12.4)	7.4 (7.5)	13.1 (13.1)
**Refinement**								
Resolution (Å)	50-1.90	47-2.05	47.05-1.95	46.68-1.50	43.33-1.25	43.33-1.90	43.5-1.45	43.4-1.50
No. reflections	64689	52588	60669	130275	122574	36224	81098	72408
R_{work} / R_{free}	0.176/0.196	0.225/0.263	0.199/0.221	0.140/0.176	0.130/0.159	0.167/0.194	0.156/0.172	0.131/0.162
Non-H atoms								
Protein	6208	6150	6194	6274	3223	3132	3150	3169
Ligand/ion	64	132	132	144	32	68	62	68
Water	400	293	355	641	519	215	398	383
B-factors (Å^2)								
Protein	57.6	80.3	72.6	34.6	21.8	40.3	30.0	28.9
Ligand/ion	34.0	51.4	46.7	23.1	12.5	30.5	20.2	21.1
Water	47.7	53.4	53.3	42.0	36.4	40.3	40.4	40.7
Ramachandran:								
Favoured (%)	97.0	97.1	97.9	97.6	98.2	98.4	98.4	98.4
Allowed (%)	3.0	2.9	2.1	2.4	1.83	1.57	1.57	1.56
Outliers (%)	0	0	0	0	0	0	0	0
R.m.s. deviations								
Bond lengths (Å)	0.007	0.002	0.004	0.007	0.005	0.005	0.007	0.008
Bond angles (°)	1.03	0.538	0.690	1.01	0.875	0.838	1.01	1.13

*Each dataset was collected from a single crystal. Values in parentheses are for highest-resolution shell (10 % of reflections).

Supplementary Table 3: Small molecule X-ray crystallography data collection and refinement statistics

**Compound**	BNS-22	Topobexin (**9**)
CCDC #	2354203	2354347
Formula	$C_{24}H_{25}NO_5$	$C_{29}H_{37}Cl_2N_3O_4$
Formula Weight	407.47	513.24
Space Group	P-1	$P2_1/n$
Flack Parameter	n/a	n/a
Cell Dimensions:		
a/Å	9.6814	17.560
b/Å	9.7569	7.3543
c/Å	11.232	21.712
a/°	78.836	90
b/°	87.650	95.231
g/°	72.619	90
Volume/Å^3	993.25	2792.5
Z (molecules per unit cell)	2	4
Wavelength/Å	1.5418	1.5418
Radiation type	Cu Kα	Cu Kα
Measured Refl's.	31793	88959
Indep't Refl's	4010	5753
Resolution rage/Å	9.20-0.80	14.25-0.80
R_{int}	0.0213	0.0447
Parameters	274	349
Restraints	0	0
Largest Peak/eÅ^{-3}	0.27	0.52
Deepest Hole/eÅ^{-3}	-0.34	-0.43
wR_2 (all data)	0.1112	0.1100
wR_2	0.1110	0.1076
R_1 (all data)	0.0417	0.0443
R_1	0.0414	0.0405
Structure	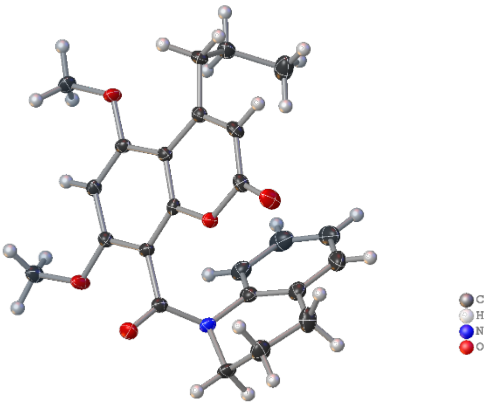	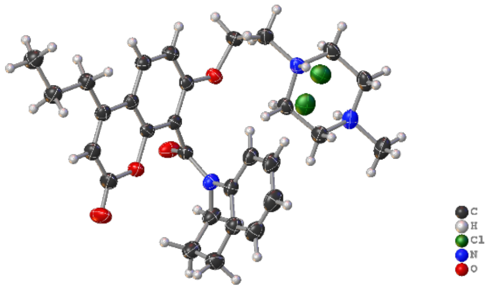

Supplementary Table 4: Summary of gene expression assays used for qPCR

Gene symbol	Protein name	Producer	qPCR assay	Sequence for design (Gene accession number)
Col1a1	Collagen type I α 1	GB	ocCOL1A1_Q1	AY633663
Fn1	Fibronectin 1	AB	Oc06726463_m1	XM_002712573.1
Hprt1	Hypoxanthine guanine phosphoribosyl transferase 1	GB	ocHPRT1_Q3	NM_001105671
Nppb	Natriuretic peptide B (ANP)	GB	ocNPPB_Q1	XM_008275383.2

Individual gene expression assays were obtained from Generi Biotech (GB) or Applied Biosystems (ABI).

Supplementary Table 5: P values and statistical tests from this study

Ctr = untreated control, ANOVA = One-way ANOVA with the Holm-Sidak post hoc test, Supp = Supplementary Figure, N = number of independent replicates. For results in Fig. 5 the numbers of animals in each experimental group were as follows: Ctr (n=9), topobexin (n=7), DAU (n=10), topobexin + DAU (n=11).

Figure	Test	N	Comparison	P value	Summary
1c	ANOVA	4	Ctr vs. DAU [1.2 µM]	<0.0001	****
			Ctr vs. 5c [0.01 µM] + DAU [1.2 µM]	<0.0001	****
			Ctr vs. 5c [0.1 µM] + DAU [1.2 µM]	<0.0001	****
			Ctr vs. 5c [1 µM] + DAU [1.2 µM]	<0.0001	****
			Ctr vs. 5c [10 µM] + DAU [1.2 µM]	0.0019	**
			Ctr vs. 5c [30 µM] + DAU [1.2 µM]	0.0012	**
			Ctr vs. 5c [100 µM] + DAU [1.2 µM]	<0.0001	****
			DAU [1.2 µM] vs. 5c [0.01 µM] + DAU [1.2 µM]	0.0825	ns
			DAU [1.2 µM] vs. 5c [0.1 µM] + DAU [1.2 µM]	0.0102	*
			DAU [1.2 µM] vs. 5c [1 µM] + DAU [1.2 µM]	0.0007	***
			DAU [1.2 µM] vs. 5c [10 µM] + DAU [1.2 µM]	<0.0001	****
			DAU [1.2 µM] vs. 5c [30 µM] + DAU [1.2 µM]	<0.0001	****
			DAU [1.2 µM] vs. 5c [100 µM] + DAU [1.2 µM]	0.0004	***
4b	ANOVA		TOP2A: Ctr vs. topobexin (9) [10 µM]	0.9806	ns
			TOP2A: Ctr vs. etoposide [5 µM]	<0.0001	****
			TOP2A: Ctr vs. topobexin (9) [0.1 µM] + etoposide [5 µM]	<0.0001	****

Figure	Test	N	Comparison	P value	Summary
			TOP2A: Ctr vs. topobexin (9) [1 µM] + etoposide [5 µM]	0.0001	***
			TOP2A: Ctr vs. topobexin (9) [10 µM] + etoposide [5 µM]	0.4735	ns
			TOP2A: etoposide [5 µM] vs. topobexin (9) [0.1 µM] + etoposide [5 µM]	0.9509	ns
			TOP2A: etoposide [5 µM] vs. topobexin (9) [1 µM] + etoposide [5 µM]	0.3483	ns
			TOP2A: etoposide [5 µM] vs. topobexin (9) [10 µM] + etoposide [5 µM]	<0.0001	****
			TOP2B: Ctr vs. topobexin (9) [10 µM]	0.9883	ns
			TOP2B: Ctr vs. etoposide [5 µM]	<0.0001	****
			TOP2B: Ctr vs. topobexin (9) [0.1 µM] + etoposide [5 µM]	0.0006	***
			TOP2B: Ctr vs. topobexin (9) [1 µM] + etoposide [5 µM]	0.3078	ns
			TOP2B: Ctr vs. topobexin (9) [10 µM] + etoposide [5 µM]	0.9822	ns
			TOP2B: etoposide [5 µM] vs. topobexin (9) [0.1 µM] + etoposide [5 µM]	0.1431	ns
			TOP2B: etoposide [5 µM] vs. topobexin (9) [1 µM] + etoposide [5 µM]	0.0004	***
			TOP2B: etoposide [5 µM] vs. topobexin (9) [10 µM] + etoposide [5 µM]	<0.0001	****
	Unpaired t test with Welch's correction, two-tailed		etoposide [5 µM]: TOP2A vs. TOP2B	>0.9999	ns
			topobexin (9) [0.1 µM] + etoposide [5 µM]: TOP2A vs. TOP2B	0.2668	ns
			topobexin (9) [1 µM] + etoposide [5 µM]: TOP2A vs. TOP2B	0.0191	*
			topobexin (9) [10 µM] + etoposide [5 µM]: TOP2A vs. TOP2B	0.0315	*
4c	ANOVA	4	Ctr vs. topobexin (9) [100 µM]	0.9642	ns
			Ctr vs. DAU [1.2 µM]	<0.0001	****
			Ctr vs. topobexin (9) [0.01 µM] + DAU [1.2 µM]	<0.0001	****
			Ctr vs. topobexin (9) [0.1 µM] + DAU [1.2 µM]	<0.0001	****
			Ctr vs. topobexin (9) [1 µM] + DAU [1.2 µM]	<0.0001	****
			Ctr vs. topobexin (9) [10 µM] + DAU [1.2 µM]	0.0037	**
			Ctr vs. topobexin (9) [30 µM] + DAU [1.2 µM]	0.0014	**
			Ctr vs. topobexin (9) [100 µM] + DAU [1.2 µM]	0.0003	***
			DAU [1.2 µM] vs. topobexin (9) [0.01 µM] + DAU [1.2 µM]	0.6901	ns

Figure	Test	N	Comparison	P value	Summary
			DAU [1.2 µM] vs. topobexin (9) [0.1 µM] + DAU [1.2 µM]	0.5031	ns
			DAU [1.2 µM] vs. topobexin (9) [1 µM] + DAU [1.2 µM]	0.0062	**
			DAU [1.2 µM] vs. topobexin (9) [10 µM] + DAU [1.2 µM]	0.0002	***
			DAU [1.2 µM] vs. topobexin (9) [30 µM] + DAU [1.2 µM]	0.0003	***
			DAU [1.2 µM] vs. topobexin (9) [100 µM] + DAU [1.2 µM]	0.0009	***
4d	ANOVA	3	Ctr vs. topobexin (9) [0.1 µM]	0.0002	***
			Ctr vs. topobexin (9) [1 µM]	0.0001	***
			Ctr vs. topobexin (9) [10 µM]	0.0001	***
4e	ANOVA	3	Ctr vs. topobexin (9) [0.1 µM]	0.9998	ns
			Ctr vs. topobexin (9) [1 µM]	0.9998	ns
			Ctr vs. topobexin (9) [10 µM]	0.9998	ns
			Ctr vs. DAU [1.2 µM]	<0.0001	****
			Ctr vs. topobexin (9) [0.1 µM] + DAU [1.2 µM]	<0.0001	****
			Ctr vs. topobexin (9) [1 µM] + DAU [1.2 µM]	0.9482	ns
			Ctr vs. topobexin (9) [10 µM] + DAU [1.2 µM]	0.9998	ns
			DAU [1.2 µM] vs. topobexin (9) [0.1 µM] + DAU [1.2 µM]	<0.0001	****
			DAU [1.2 µM] vs. topobexin (9) [1 µM] + DAU [1.2 µM]	<0.0001	****
			DAU [1.2 µM] vs. topobexin (9) [10 µM] + DAU [1.2 µM]	<0.0001	****
4f	Ratio paired t-test, two-tailed	6	Csp3/7: Cmpd 9 + DAU vs. DAU	<0.0001	****
			Csp8: Cmpd 9 + DAU vs. DAU	0.0387	*
			Csp9: Cmpd 9 + DAU vs. DAU	0.0001	***
5b	ANOVA	6	topobexin+DAU vs. DAU	<0.001	***
			CTR vs. DAU	<0.001	***
			topobexin vs. DAU	<0.001	***
			topobexin+DAU vs. CTR	0.947	No
			topobexin+DAU vs. topobexin	0.902	No
			topobexin vs. CTR	0.932	No
5d	ANOVA	7–11	topobexin vs. DAU	<0.001	***
			topobexin vs. topobexin+DAU	0.004	**
			CTR vs. DAU	0.024	*

Figure	Test	N	Comparison	P value	Summary
			topobexin vs. CTR	0.164	ns
			CTR vs. topobexin+DAU	0.267	ns
			topobexin+DAU vs. DAU	0.221	ns
5e	ANOVA	7–11	topobexin+DAU vs. DAU	<0.001	***
			CTR vs. DAU	<0.001	***
			topobexin vs. DAU	<0.001	***
			topobexin+DAU vs. CTR	0.953	ns
			topobexin vs. CTR	0.906	ns
			topobexin+DAU vs. topobexin	0.979	ns
5f	ANOVA	7–11	topobexin+DAU vs. DAU	<0.001	***
			CTR vs. DAU	<0.001	***
			topobexin vs. DAU	<0.001	***
			topobexin+DAU vs. topobexin	0.982	ns
			topobexin+DAU vs. CTR	0.952	ns
			CTR vs. topobexin	0.943	ns
5g	ANOVA	7–11	topobexin+DAU vs. DAU	<0.001	***
			CTR vs. DAU	<0.001	***
			topobexin vs. DAU	<0.001	***
			CTR vs. topobexin+DAU	0.453	ns
			topobexin vs. topobexin+DAU	0.669	ns
			CTR vs. topobexin	0.672	ns
5h	Kruskal-Wallis with the Dunn's post hoc test	7–11	topobexin vs DAU	0.003	**
			CTR vs DAU	0.002	**
			topobexin+DAU vs DAU	0.017	*
			topobexin+DAU vs topobexin	1	ns
			topobexin+DAU vs CTR	1	ns
			CTR vs topobexin	1	ns
5i	ANOVA	7–11	CTR vs. DAU	<0.001	***

Figure	Test	N	Comparison	P value	Summary
			topobexin vs. DAU	0.002	**
			topobexin+DAU vs. DAU	0.025	*
			topobexin+DAU vs. CTR	0.122	ns
			topobexin+DAU vs. topobexin	0.229	ns
			topobexin vs. CTR	0.638	ns
5j	ANOVA	7–11	topobexin vs DAU	<0.001	***
			CTR vs DAU	<0.001	***
			topobexin+DAU vs DAU	<0.001	***
			topobexin+DAU vs. CTR	0.992	ns
			topobexin vs. CTR	0.988	ns
			topobexin+DAU vs. topobexin	0.928	ns
5k	ANOVA	7–11	topobexin vs DAU	<0.001	***
			CTR vs DAU	<0.001	***
			topobexin+DAU vs DAU	<0.001	***
			topobexin+DAU vs. CTR	0.859	ns
			topobexin+DAU vs. topobexin	0.886	ns
			topobexin vs. CTR	0.831	ns
**SI**					
Supp 2f	ANOVA	4	Ctr vs. DAU [1.2 µM]	<0.0001	****
			Ctr vs. BNS-22 [0.01 µM] + DAU [1.2 µM]	<0.0001	****
			Ctr vs. BNS-22 [0.1 µM] + DAU [1.2 µM]	<0.0001	****
			Ctr vs. BNS-22 [1 µM] + DAU [1.2 µM]	<0.0001	****
			Ctr vs. BNS-22 [3 µM] + DAU [1.2 µM]	0.0005	***
			Ctr vs. BNS-22 [10 µM] + DAU [1.2 µM]	0.0015	**
			DAU [1.2 µM] vs. BNS-22 [0.01 µM] + DAU [1.2 µM]	0.8002	ns
			DAU [1.2 µM] vs. BNS-22 [0.1 µM] + DAU [1.2 µM]	0.0755	ns
			DAU [1.2 µM] vs. BNS-22 [1 µM] + DAU [1.2 µM]	0.0012	**
			DAU [1.2 µM] vs. BNS-22 [3 µM] + DAU [1.2 µM]	<0.0001	****
			DAU [1.2 µM] vs. BNS-22 [10 µM] + DAU [1.2 µM]	<0.0001	****

Figure	Test	N	Comparison	P value	Summary
Supp 3	ANOVA	4	1: Ctr vs. DAU [1.2 µM]	<0.0001	****
			1: Ctr vs. BNS-22 [0.01 µM] + DAU [1.2 µM]	<0.0001	****
			1: Ctr vs. BNS-22 [0.1 µM] + DAU [1.2 µM]	<0.0001	****
			1: Ctr vs. BNS-22 [1 µM] + DAU [1.2 µM]	<0.0001	****
			1: Ctr vs. BNS-22 [3 µM] + DAU [1.2 µM]	0.0003	***
			1: Ctr vs. BNS-22 [10 µM] + DAU [1.2 µM]	0.0009	***
	ANOVA	4	1: DAU [1.2 µM] vs. BNS-22 [0.01 µM] + DAU [1.2 µM]	0.8002	ns
			1: DAU [1.2 µM] vs. BNS-22 [0.1 µM] + DAU [1.2 µM]	0.0755	ns
			1: DAU [1.2 µM] vs. BNS-22 [1 µM] + DAU [1.2 µM]	0.0012	**
			1: DAU [1.2 µM] vs. BNS-22 [3 µM] + DAU [1.2 µM]	<0.0001	****
			1: DAU [1.2 µM] vs. BNS-22 [10 µM] + DAU [1.2 µM]	<0.0001	****
	ANOVA	4	1d: Ctr vs. DAU [1.2 µM]	<0.0001	****
			1d: Ctr vs. 1d [0.1 µM] + DAU [1.2 µM]	<0.0001	****
			1d: Ctr vs. 1d [1 µM] + DAU [1.2 µM]	0.0007	***
			1d: Ctr vs. 1d [10 µM] + DAU [1.2 µM]	<0.0001	****
			1d: Ctr vs. 1d [30 µM] + DAU [1.2 µM]	<0.0001	****
			1d: Ctr vs. 1d [100 µM] + DAU [1.2 µM]	<0.0001	****
	ANOVA	4	1d: DAU [1.2 µM] vs. 1d [0.1 µM] + DAU [1.2 µM]	0.0638	ns
			1d: DAU [1.2 µM] vs. 1d [1 µM] + DAU [1.2 µM]	0.0002	***
			1d: DAU [1.2 µM] vs. 1d [10 µM] + DAU [1.2 µM]	0.0009	***
			1d: DAU [1.2 µM] vs. 1d [30 µM] + DAU [1.2 µM]	0.0003	***
			1d: DAU [1.2 µM] vs. 1d [100 µM] + DAU [1.2 µM]	<0.0001	****
	ANOVA	4	2a: Ctr vs. DAU [1.2 µM]	<0.0001	****
			2a: Ctr vs. 2a [10 µM] + DAU [1.2 µM]	<0.0001	****
			2a: Ctr vs. 2a [30 µM] + DAU [1.2 µM]	<0.0001	****
			2a: Ctr vs. 2a [100 µM] + DAU [1.2 µM]	<0.0001	****
	ANOVA	4	2a: DAU [1.2 µM] vs. 2a [10 µM] + DAU [1.2 µM]	0.9188	ns
			2a: DAU [1.2 µM] vs. 2a [30 µM] + DAU [1.2 µM]	0.9188	ns
			2a: DAU [1.2 µM] vs. 2a [100 µM] + DAU [1.2 µM]	0.9188	ns
	ANOVA	4	2b: Ctr vs. DAU [1.2 µM]	<0.0001	****
			2b: Ctr vs. 2b [10 µM] + DAU [1.2 µM]	<0.0001	****

Figure	Test	N	Comparison	P value	Summary
			2b: Ctr vs. 2b [30 µM] + DAU [1.2 µM]	<0.0001	****
			2b: Ctr vs. 2b [100 µM] + DAU [1.2 µM]	<0.0001	****
	ANOVA	4	2b: DAU [1.2 µM] vs. 2b [10 µM] + DAU [1.2 µM]	0.1288	ns
			2b: DAU [1.2 µM] vs. 2b [30 µM] + DAU [1.2 µM]	0.1919	ns
			2b: DAU [1.2 µM] vs. 2b [100 µM] + DAU [1.2 µM]	0.1288	ns
	ANOVA	4	2c: Ctr vs. DAU [1.2 µM]	<0.0001	****
			2c: Ctr vs. 2c [1 µM] + DAU [1.2 µM]	<0.0001	****
			2c: Ctr vs. 2c [10 µM] + DAU [1.2 µM]	<0.0001	****
			2c: Ctr vs. 2c [30 µM] + DAU [1.2 µM]	0.0035	**
			2c: Ctr vs. 2c [100 µM] + DAU [1.2 µM]	<0.0001	****
	ANOVA	4	2c: DAU [1.2 µM] vs. 2c [1 µM] + DAU [1.2 µM]	0.1694	ns
			2c: DAU [1.2 µM] vs. 2c [10 µM] + DAU [1.2 µM]	0.0171	*
			2c: DAU [1.2 µM] vs. 2c [30 µM] + DAU [1.2 µM]	0.0021	**
			2c: DAU [1.2 µM] vs. 2c [100 µM] + DAU [1.2 µM]	0.2739	ns
	ANOVA	4	2d: Ctr vs. DAU [1.2 µM]	<0.0001	****
			2d: Ctr vs. 2d [0.1 µM] + DAU [1.2 µM]	<0.0001	****
			2d: Ctr vs. 2d [1 µM] + DAU [1.2 µM]	<0.0001	****
			2d: Ctr vs. 2d [10 µM] + DAU [1.2 µM]	0.0031	**
			2d: Ctr vs. 2d [30 µM] + DAU [1.2 µM]	0.0128	*
			2d: Ctr vs. 2d [100 µM] + DAU [1.2 µM]	<0.0001	****
	ANOVA	4–5	2d: DAU [1.2 µM] vs. 2d [0.1 µM] + DAU [1.2 µM]	0.5859	ns
			2d: DAU [1.2 µM] vs. 2d [1 µM] + DAU [1.2 µM]	0.3259	ns
			2d: DAU [1.2 µM] vs. 2d [10 µM] + DAU [1.2 µM]	0.0272	*
			2d: DAU [1.2 µM] vs. 2d [30 µM] + DAU [1.2 µM]	0.0117	*
			2d: DAU [1.2 µM] vs. 2d [100 µM] + DAU [1.2 µM]	<0.0001	****
	ANOVA	4	3a: Ctr vs. DAU [1.2 µM]	<0.0001	****
			3a: Ctr vs. 3a [1 µM] + DAU [1.2 µM]	<0.0001	****
			3a: Ctr vs. 3a [3 µM] + DAU [1.2 µM]	<0.0001	****
			3a: Ctr vs. 3a [10 µM] + DAU [1.2 µM]	<0.0001	****
	ANOVA	4–5	3a: DAU [1.2 µM] vs. 3a [1 µM] + DAU [1.2 µM]	0.7736	ns
			3a: DAU [1.2 µM] vs. 3a [3 µM] + DAU [1.2 µM]	0.7736	ns

Figure	Test	N	Comparison	P value	Summary
			3a: DAU [1.2 µM] vs. 3a [10 µM] + DAU [1.2 µM]	0.7736	ns
	ANOVA	4	3b: Ctr vs. DAU [1.2 µM]	<0.0001	****
			3b: Ctr vs. 3b [1 µM] + DAU [1.2 µM]	<0.0001	****
			3b: Ctr vs. 3b [3 µM] + DAU [1.2 µM]	<0.0001	****
			3b: Ctr vs. 3b [10 µM] + DAU [1.2 µM]	<0.0001	****
	ANOVA	4	3b: DAU [1.2 µM] vs. 3b [1 µM] + DAU [1.2 µM]	0.3074	ns
			3b: DAU [1.2 µM] vs. 3b [3 µM] + DAU [1.2 µM]	0.3634	ns
			3b: DAU [1.2 µM] vs. 3b [10 µM] + DAU [1.2 µM]	0.2150	ns
	ANOVA	4	3c: Ctr vs. DAU [1.2 µM]	<0.0001	****
			3c: Ctr vs. 3c [0.1 µM] + DAU [1.2 µM]	<0.0001	****
			3c: Ctr vs. 3c [1 µM] + DAU [1.2 µM]	<0.0001	****
			3c: Ctr vs. 3c [3 µM] + DAU [1.2 µM]	<0.0001	****
			3c: Ctr vs. 3c [10 µM] + DAU [1.2 µM]	0.0012	**
	ANOVA	4	3c: DAU [1.2 µM] vs. 3c [0.1 µM] + DAU [1.2 µM]	0.1762	ns
			3c: DAU [1.2 µM] vs. 3c [1 µM] + DAU [1.2 µM]	0.0093	**
			3c: DAU [1.2 µM] vs. 3c [3 µM] + DAU [1.2 µM]	0.0014	**
			3c: DAU [1.2 µM] vs. 3c [10 µM] + DAU [1.2 µM]	0.0001	***
	ANOVA	4	3d: Ctr vs. DAU [1.2 µM]	<0.0001	****
			3d: Ctr vs. 3d [0.01 µM] + DAU [1.2 µM]	<0.0001	****
			3d: Ctr vs. 3d [0.1 µM] + DAU [1.2 µM]	<0.0001	****
			3d: Ctr vs. 3d [1 µM] + DAU [1.2 µM]	<0.0001	****
			3d: Ctr vs. 3d [10 µM] + DAU [1.2 µM]	0.0496	*
			3d: Ctr vs. 3d [30 µM] + DAU [1.2 µM]	0.0010	**
	ANOVA	4	3d: DAU [1.2 µM] vs. 3d [0.01 µM] + DAU [1.2 µM]	0.9523	ns
			3d: DAU [1.2 µM] vs. 3d [0.1 µM] + DAU [1.2 µM]	0.0926	ns
			3d: DAU [1.2 µM] vs. 3d [1 µM] + DAU [1.2 µM]	0.0208	*
			3d: DAU [1.2 µM] vs. 3d [10 µM] + DAU [1.2 µM]	0.0002	***
			3d: DAU [1.2 µM] vs. 3d [30 µM] + DAU [1.2 µM]	0.0023	**
	ANOVA	4	4a: Ctr vs. DAU [1.2 µM]	<0.0001	****
			4a: Ctr vs. 4a [0.1 µM] + DAU [1.2 µM]	<0.0001	****
			4a: Ctr vs. 4a [1 µM] + DAU [1.2 µM]	<0.0001	****

Figure	Test	N	Comparison	P value	Summary
			4a: Ctr vs. 4a [10 µM] + DAU [1.2 µM]	<0.0001	****
			4a: Ctr vs. 4a [30 µM] + DAU [1.2 µM]	<0.0001	****
	ANOVA	4–5	4a: DAU [1.2 µM] vs. 4a [0.1 µM] + DAU [1.2 µM]	0.4863	ns
			4a: DAU [1.2 µM] vs. 4a [1 µM] + DAU [1.2 µM]	0.1212	ns
			4a: DAU [1.2 µM] vs. 4a [10 µM] + DAU [1.2 µM]	0.0003	***
			4a: DAU [1.2 µM] vs. 4a [30 µM] + DAU [1.2 µM]	<0.0001	****
	ANOVA	4	4b: Ctr vs. DAU [1.2 µM]	<0.0001	****
			4b: Ctr vs. 4b [0.01 µM] + DAU [1.2 µM]	<0.0001	****
			4b: Ctr vs. 4b [0.1 µM] + DAU [1.2 µM]	<0.0001	****
			4b: Ctr vs. 4b [1 µM] + DAU [1.2 µM]	<0.0001	****
			4b: Ctr vs. 4b [10 µM] + DAU [1.2 µM]	<0.0001	****
			4b: Ctr vs. 4b [30 µM] + DAU [1.2 µM]	0.0001	***
			4b: Ctr vs. 4b [100 µM] + DAU [1.2 µM]	<0.0001	****
	ANOVA	4	4b: DAU [1.2 µM] vs. 4b [0.01 µM] + DAU [1.2 µM]	0.1177	ns
			4b: DAU [1.2 µM] vs. 4b [0.1 µM] + DAU [1.2 µM]	0.0124	*
			4b: DAU [1.2 µM] vs. 4b [1 µM] + DAU [1.2 µM]	<0.0001	****
			4b: DAU [1.2 µM] vs. 4b [10 µM] + DAU [1.2 µM]	<0.0001	****
			4b: DAU [1.2 µM] vs. 4b [30 µM] + DAU [1.2 µM]	<0.0001	****
			4b: DAU [1.2 µM] vs. 4b [100 µM] + DAU [1.2 µM]	<0.0001	****
	ANOVA	4	4c: Ctr vs. DAU [1.2 µM]	<0.0001	****
			4c: Ctr vs. 4c [0.01 µM] + DAU [1.2 µM]	0.0002	***
			4c: Ctr vs. 4c [0.1 µM] + DAU [1.2 µM]	0.0106	*
			4c: Ctr vs. 4c [1 µM] + DAU [1.2 µM]	0.2503	ns
			4c: Ctr vs. 4c [10 µM] + DAU [1.2 µM]	0.0313	*
			4c: Ctr vs. 4c [30 µM] + DAU [1.2 µM]	<0.0001	****
			4c: Ctr vs. 4c [100 µM] + DAU [1.2 µM]	<0.0001	****
	ANOVA	4	4c: DAU [1.2 µM] vs. 4c [0.01 µM] + DAU [1.2 µM]	0.4467	ns
			4c: DAU [1.2 µM] vs. 4c [0.1 µM] + DAU [1.2 µM]	0.0392	*
			4c: DAU [1.2 µM] vs. 4c [1 µM] + DAU [1.2 µM]	0.0011	**
			4c: DAU [1.2 µM] vs. 4c [10 µM] + DAU [1.2 µM]	0.0162	*
			4c: DAU [1.2 µM] vs. 4c [30 µM] + DAU [1.2 µM]	0.4467	ns

Figure	Test	N	Comparison	P value	Summary
			4c: DAU [1.2 µM] vs. 4c [100 µM] + DAU [1.2 µM]	0.0004	***
	ANOVA	4	4d: Ctr vs. DAU [1.2 µM]	<0.0001	****
			4d: Ctr vs. 4d [0.01 µM] + DAU [1.2 µM]	0.0003	***
			4d: Ctr vs. 4d [0.1 µM] + DAU [1.2 µM]	0.0063	**
			4d: Ctr vs. 4d [1 µM] + DAU [1.2 µM]	0.1423	ns
			4d: Ctr vs. 4d [10 µM] + DAU [1.2 µM]	<0.0001	****
			4d: Ctr vs. 4d [30 µM] + DAU [1.2 µM]	<0.0001	****
			4d: Ctr vs. 4d [100 µM] + DAU [1.2 µM]	<0.0001	****
	ANOVA	4	4d: DAU [1.2 µM] vs. 4d [0.01 µM] + DAU [1.2 µM]	0.4723	ns
			4d: DAU [1.2 µM] vs. 4d [0.1 µM] + DAU [1.2 µM]	0.1168	ns
			4d: DAU [1.2 µM] vs. 4d [1 µM] + DAU [1.2 µM]	0.0106	*
			4d: DAU [1.2 µM] vs. 4d [10 µM] + DAU [1.2 µM]	0.7305	ns
			4d: DAU [1.2 µM] vs. 4d [30 µM] + DAU [1.2 µM]	<0.0001	****
			4d: DAU [1.2 µM] vs. 4d [100 µM] + DAU [1.2 µM]	<0.0001	****
	ANOVA	4	5a: Ctr vs. DAU [1.2 µM]	<0.0001	****
			5a: Ctr vs. 5a [1 µM] + DAU [1.2 µM]	<0.0001	****
			5a: Ctr vs. 5a [10 µM] + DAU [1.2 µM]	<0.0001	****
			5a: Ctr vs. 5a [30 µM] + DAU [1.2 µM]	<0.0001	****
			5a: Ctr vs. 5a [100 µM] + DAU [1.2 µM]	<0.0001	****
	ANOVA	4–5	5a: DAU [1.2 µM] vs. 5a [1 µM] + DAU [1.2 µM]	0.3090	ns
			5a: DAU [1.2 µM] vs. 5a [10 µM] + DAU [1.2 µM]	0.3090	ns
			5a: DAU [1.2 µM] vs. 5a [30 µM] + DAU [1.2 µM]	0.0065	**
			5a: DAU [1.2 µM] vs. 5a [100 µM] + DAU [1.2 µM]	0.0009	***
	ANOVA	4	5b: Ctr vs. DAU [1.2 µM]	<0.0001	****
			5b: Ctr vs. 5b [0.1 µM] + DAU [1.2 µM]	<0.0001	****
			5b: Ctr vs. 5b [1 µM] + DAU [1.2 µM]	<0.0001	****
			5b: Ctr vs. 5b [10 µM] + DAU [1.2 µM]	<0.0001	****
			5b: Ctr vs. 5b [30 µM] + DAU [1.2 µM]	<0.0001	****
	ANOVA	4	5b: DAU [1.2 µM] vs. 5b [0.1 µM] + DAU [1.2 µM]	0.7670	ns
			5b: DAU [1.2 µM] vs. 5b [1 µM] + DAU [1.2 µM]	0.5634	ns
			5b: DAU [1.2 µM] vs. 5b [10 µM] + DAU [1.2 µM]	0.0064	**

Figure	Test	N	Comparison	P value	Summary
			5b: DAU [1.2 µM] vs. 5b [30 µM] + DAU [1.2 µM]	0.0007	***
	ANOVA	4	5c: Ctr vs. DAU [1.2 µM]	<0.0001	****
			5c: Ctr vs. 5c [0.01 µM] + DAU [1.2 µM]	<0.0001	****
			5c: Ctr vs. 5c [0.1 µM] + DAU [1.2 µM]	<0.0001	****
			5c: Ctr vs. 5c [1 µM] + DAU [1.2 µM]	<0.0001	****
			5c: Ctr vs. 5c [10 µM] + DAU [1.2 µM]	0.0019	**
			5c: Ctr vs. 5c [30 µM] + DAU [1.2 µM]	0.0012	**
			5c: Ctr vs. 5c [100 µM] + DAU [1.2 µM]	<0.0001	****
	ANOVA	4	5c: DAU [1.2 µM] vs. 5c [0.01 µM] + DAU [1.2 µM]	0.0825	ns
			5c: DAU [1.2 µM] vs. 5c [0.1 µM] + DAU [1.2 µM]	0.0102	*
			5c: DAU [1.2 µM] vs. 5c [1 µM] + DAU [1.2 µM]	0.0007	***
			5c: DAU [1.2 µM] vs. 5c [10 µM] + DAU [1.2 µM]	<0.0001	****
			5c: DAU [1.2 µM] vs. 5c [30 µM] + DAU [1.2 µM]	<0.0001	****
			5c: DAU [1.2 µM] vs. 5c [100 µM] + DAU [1.2 µM]	0.0004	***
	ANOVA	4	5d: Ctr vs. DAU [1.2 µM]	<0.0001	****
			5d: Ctr vs. 5d [0.01 µM] + DAU [1.2 µM]	<0.0001	****
			5d: Ctr vs. 5d [0.1 µM] + DAU [1.2 µM]	<0.0001	****
			5d: Ctr vs. 5d [1 µM] + DAU [1.2 µM]	<0.0001	****
			5d: Ctr vs. 5d [10 µM] + DAU [1.2 µM]	0.0001	***
			5d: Ctr vs. 5d [30 µM] + DAU [1.2 µM]	<0.0001	****
			5d: Ctr vs. 5d [100 µM] + DAU [1.2 µM]	<0.0001	****
	ANOVA	4	5d: DAU [1.2 µM] vs. 5d [0.01 µM] + DAU [1.2 µM]	0.9271	ns
			5d: DAU [1.2 µM] vs. 5d [0.1 µM] + DAU [1.2 µM]	0.9271	ns
			5d: DAU [1.2 µM] vs. 5d [1 µM] + DAU [1.2 µM]	0.1610	ns
			5d: DAU [1.2 µM] vs. 5d [10 µM] + DAU [1.2 µM]	0.0551	ns
			5d: DAU [1.2 µM] vs. 5d [30 µM] + DAU [1.2 µM]	0.1732	ns
			5d: DAU [1.2 µM] vs. 5d [100 µM] + DAU [1.2 µM]	0.9271	ns
Supp 4	ANOVA	4	1: Ctr vs. BNS-22 [0.01 µM]	0.9742	ns
			1: Ctr vs. BNS-22 [0.1 µM]	0.9742	ns
			1: Ctr vs. BNS-22 [1 µM]	0.9742	ns
			1: Ctr vs. BNS-22 [3 µM]	0.9742	ns

Figure	Test	N	Comparison	P value	Summary
			1: Ctr vs. BNS-22 [10 µM]	0.6836	ns
	ANOVA	4	1d: Ctr vs. 1d [0.1 µM]	0.8671	ns
			1d: Ctr vs. 1d [1 µM]	0.8671	ns
			1d: Ctr vs. 1d [10 µM]	0.1840	ns
			1d: Ctr vs. 1d [30 µM]	<0.0001	****
			1d: Ctr vs. 1d [100 µM]	<0.0001	****
	ANOVA	4	2a: Ctr vs. 2a [10 µM]	0.9389	ns
			2a: Ctr vs. 2a [30 µM]	0.9389	ns
			2a: Ctr vs. 2a [100 µM]	0.9389	ns
	ANOVA	4	2b: Ctr vs. 2b [10 µM]	0.6757	ns
			2b: Ctr vs. 2b [30 µM]	0.6757	ns
			2b: Ctr vs. 2b [100 µM]	0.6757	ns
	ANOVA	4	2c: Ctr vs. 2c [1 µM]	0.9468	ns
			2c: Ctr vs. 2c [10 µM]	0.9468	ns
			2c: Ctr vs. 2c [30 µM]	0.9467	ns
			2c: Ctr vs. 2c [100 µM]	<0.0001	****
	ANOVA	4	2d: Ctr vs. 2d [0.1 µM]	0.9148	ns
			2d: Ctr vs. 2d [1 µM]	0.9148	ns
			2d: Ctr vs. 2d [10 µM]	0.8461	ns
			2d: Ctr vs. 2d [30 µM]	0.8311	ns
			2d: Ctr vs. 2d [100 µM]	<0.0001	****
	ANOVA	4	3a: Ctr vs. 3a [1 µM]	0.7160	ns
			3a: Ctr vs. 3a [3 µM]	0.9575	ns
			3a: Ctr vs. 3a [10 µM]	0.9575	ns
	ANOVA	4	3b: Ctr vs. 3b [1 µM]	0.9627	ns
			3b: Ctr vs. 3b [3 µM]	0.9627	ns
			3b: Ctr vs. 3b [10 µM]	0.9627	ns
	ANOVA	4	3c: Ctr vs. 3c [0.1 µM]	0.9958	ns
			3c: Ctr vs. 3c [1 µM]	0.9958	ns
			3c: Ctr vs. 3c [3 µM]	0.9958	ns
			3c: Ctr vs. 3c [10 µM]	0.9958	ns

Figure	Test	N	Comparison	P value	Summary
	ANOVA	4	3d: Ctr vs. 3d [0.01 µM]	0.9976	ns
			3d: Ctr vs. 3d [0.1 µM]	0.9976	ns
			3d: Ctr vs. 3d [1 µM]	0.9355	ns
			3d: Ctr vs. 3d [10 µM]	0.6273	ns
			3d: Ctr vs. 3d [30 µM]	0.6404	ns
	ANOVA	4	4a: Ctr vs. 4a [0.1 µM]	0.9789	ns
			4a: Ctr vs. 4a [1 µM]	0.9789	ns
			4a: Ctr vs. 4a [10 µM]	0.7172	ns
			4a: Ctr vs. 4a [30 µM]	0.1208	ns
	ANOVA	4	4b: Ctr vs. 4b [0.01 µM]	0.8921	ns
			4b: Ctr vs. 4b [0.1 µM]	0.8921	ns
			4b: Ctr vs. 4b [1 µM]	0.8921	ns
			4b: Ctr vs. 4b [10 µM]	0.3956	ns
			4b: Ctr vs. 4b [30 µM]	0.3956	ns
			4b: Ctr vs. 4b [100 µM]	0.3956	ns
	ANOVA	4	4c: Ctr vs. 4c [0.01 µM]	0.9900	ns
			4c: Ctr vs. 4c [0.1 µM]	0.9900	ns
			4c: Ctr vs. 4c [1 µM]	0.9900	ns
			4c: Ctr vs. 4c [10 µM]	0.5694	ns
			4c: Ctr vs. 4c [30 µM]	0.0313	*
			4c: Ctr vs. 4c [100 µM]	<0.0001	****
	ANOVA	4	4d: Ctr vs. 4d [0.01 µM]	0.9052	ns
			4d: Ctr vs. 4d [0.1 µM]	0.9518	ns
			4d: Ctr vs. 4d [1 µM]	0.9052	ns
			4d: Ctr vs. 4d [10 µM]	0.0052	**
			4d: Ctr vs. 4d [30 µM]	<0.0001	****
			4d: Ctr vs. 4d [100 µM]	<0.0001	****
	ANOVA	4	5a: Ctr vs. 5a [1 µM]	0.7394	ns
			5a: Ctr vs. 5a [10 µM]	0.9751	ns
			5a: Ctr vs. 5a [30 µM]	0.9287	ns
			5a: Ctr vs. 5a [100 µM]	0.9751	ns

Figure	Test	N	Comparison	P value	Summary
	ANOVA	4	5b: Ctr vs. 5b [0.1 µM]	0.7837	ns
			5b: Ctr vs. 5b [1 µM]	0.7837	ns
			5b: Ctr vs. 5b [10 µM]	0.7837	ns
			5b: Ctr vs. 5b [30 µM]	0.7837	ns
	ANOVA	4	5c: Ctr vs. 5c [0.01 µM]	0.9795	ns
			5c: Ctr vs. 5c [0.1 µM]	0.9795	ns
			5c: Ctr vs. 5c [1 µM]	0.9795	ns
			5c: Ctr vs. 5c [10 µM]	0.5423	ns
			5c: Ctr vs. 5c [30 µM]	0.5623	ns
			5c: Ctr vs. 5c [100 µM]	0.1222	ns
	ANOVA	4	5d: Ctr vs. 5d [0.01 µM]	0.9649	ns
			5d: Ctr vs. 5d [0.1 µM]	0.9786	ns
			5d: Ctr vs. 5d [1 µM]	0.9786	ns
			5d: Ctr vs. 5d [10 µM]	0.5967	ns
			5d: Ctr vs. 5d [30 µM]	0.0564	ns
			5d: Ctr vs. 5d [100 µM]	0.0164	*
Supp 6b	ANOVA	4	Cmpd 6: Ctr vs. DAU [1.2 µM]	<0.0001	****
			Cmpd 6: Ctr vs. Cmpd 6 [0.1 µM] + DAU [1.2 µM]	<0.0001	****
			Cmpd 6: Ctr vs. Cmpd 6 [1 µM] + DAU [1.2 µM]	<0.0001	****
			Cmpd 6: Ctr vs. Cmpd 6 [10 µM] + DAU [1.2 µM]	<0.0001	****
			Cmpd 6: Ctr vs. Cmpd 6 [30 µM] + DAU [1.2 µM]	<0.0001	****
			Cmpd 6: Ctr vs. Cmpd 6 [100 µM] + DAU [1.2 µM]	<0.0001	****
	ANOVA	4	Cmpd 6: DAU [1.2 µM] vs. Cmpd 6 [0.1 µM] + DAU [1.2 µM]	0.8741	ns
			Cmpd 6: DAU [1.2 µM] vs. Cmpd 6 [1 µM] + DAU [1.2 µM]	0.8741	ns
			Cmpd 6: DAU [1.2 µM] vs. Cmpd 6 [10 µM] + DAU [1.2 µM]	0.0491	*
			Cmpd 6: DAU [1.2 µM] vs. Cmpd 6 [30 µM] + DAU [1.2 µM]	0.0064	**
			Cmpd 6: DAU [1.2 µM] vs. Cmpd 6 [100 µM] + DAU [1.2 µM]	0.0491	*
	ANOVA	4	Cmpd 7: Ctr vs. DAU [1.2 µM]	<0.0001	****
			Cmpd 7: Ctr vs. Cmpd 7 [0.01 µM] + DAU [1.2 µM]	<0.0001	****

Figure	Test	N	Comparison	P value	Summary
			Cmpd 7: Ctr vs. Cmpd 7 [0.1 µM] + DAU [1.2 µM]	<0.0001	****
			Cmpd 7: Ctr vs. Cmpd 7 [1 µM] + DAU [1.2 µM]	<0.0001	****
			Cmpd 7: Ctr vs. Cmpd 7 [10 µM] + DAU [1.2 µM]	0.0002	***
			Cmpd 7: Ctr vs. Cmpd 7 [30 µM] + DAU [1.2 µM]	<0.0001	****
			Cmpd 7: Ctr vs. Cmpd 7 [100 µM] + DAU [1.2 µM]	<0.0001	****
	ANOVA	4	Cmpd 7: DAU [1.2 µM] vs. Cmpd 7 [0.01 µM] + DAU [1.2 µM]	0.5381	ns
			Cmpd 7: DAU [1.2 µM] vs. Cmpd 7 [0.1 µM] + DAU [1.2 µM]	0.5381	ns
			Cmpd 7: DAU [1.2 µM] vs. Cmpd 7 [1 µM] + DAU [1.2 µM]	0.1928	ns
			Cmpd 7: DAU [1.2 µM] vs. Cmpd 7 [10 µM] + DAU [1.2 µM]	0.0077	**
			Cmpd 7: DAU [1.2 µM] vs. Cmpd 7 [30 µM] + DAU [1.2 µM]	0.0219	*
			Cmpd 7: DAU [1.2 µM] vs. Cmpd 7 [100 µM] + DAU [1.2 µM]	0.1011	ns
	ANOVA	4	Cmpd 8: Ctr vs. DAU [1.2 µM]	<0.0001	****
			Cmpd 8: Ctr vs. Cmpd 8 [0.01 µM] + DAU [1.2 µM]	<0.0001	****
			Cmpd 8: Ctr vs. Cmpd 8 [0.1 µM] + DAU [1.2 µM]	<0.0001	****
			Cmpd 8: Ctr vs. Cmpd 8 [1 µM] + DAU [1.2 µM]	<0.0001	****
			Cmpd 8: Ctr vs. Cmpd 8 [10 µM] + DAU [1.2 µM]	<0.0001	****
			Cmpd 8: Ctr vs. Cmpd 8 [30 µM] + DAU [1.2 µM]	<0.0001	****
			Cmpd 8: Ctr vs. Cmpd 8 [100 µM] + DAU [1.2 µM]	<0.0001	****
	ANOVA	4	Cmpd 8: DAU [1.2 µM] vs. Cmpd 8 [0.01 µM] + DAU [1.2 µM]	0.3717	ns
			Cmpd 8: DAU [1.2 µM] vs. Cmpd 8 [0.1 µM] + DAU [1.2 µM]	0.3717	ns
			Cmpd 8: DAU [1.2 µM] vs. Cmpd 8 [1 µM] + DAU [1.2 µM]	0.0122	*
			Cmpd 8: DAU [1.2 µM] vs. Cmpd 8 [10 µM] + DAU [1.2 µM]	0.0012	**
			Cmpd 8: DAU [1.2 µM] vs. Cmpd 8 [30 µM] + DAU [1.2 µM]	0.0054	**
			Cmpd 8: DAU [1.2 µM] vs. Cmpd 8 [100 µM] + DAU [1.2 µM]	0.3717	ns
	ANOVA	4	topobexin (9): Ctr vs. DAU [1.2 µM]	<0.0001	****
			topobexin (9): Ctr vs. topobexin (9) [0.01 µM] + DAU [1.2 µM]	<0.0001	****

Figure	Test	N	Comparison	P value	Summary
			topobexin (9): Ctr vs. topobexin (9) [0.1 µM] + DAU [1.2 µM]	<0.0001	****
			topobexin (9): Ctr vs. topobexin (9) [1 µM] + DAU [1.2 µM]	<0.0001	****
			topobexin (9): Ctr vs. topobexin (9) [10 µM] + DAU [1.2 µM]	0.0037	**
			topobexin (9): Ctr vs. topobexin (9) [30 µM] + DAU [1.2 µM]	0.0014	**
			topobexin (9): Ctr vs. topobexin (9) [100 µM] + DAU [1.2 µM]	0.0003	***
	ANOVA	4	topobexin (9): DAU [1.2 µM] vs. topobexin (9) [0.01 µM] + DAU [1.2 µM]	0.6901	ns
			topobexin (9): DAU [1.2 µM] vs. topobexin (9) [0.1 µM] + DAU [1.2 µM]	0.5031	ns
			topobexin (9): DAU [1.2 µM] vs. topobexin (9) [1 µM] + DAU [1.2 µM]	0.0062	**
			topobexin (9): DAU [1.2 µM] vs. topobexin (9) [10 µM] + DAU [1.2 µM]	0.0002	***
			topobexin (9): DAU [1.2 µM] vs. topobexin (9) [30 µM] + DAU [1.2 µM]	0.0003	***
			topobexin (9): DAU [1.2 µM] vs. topobexin (9) [100 µM] + DAU [1.2 µM]	0.0009	***
Supp 6c	ANOVA	4	Cmpd 6: Ctr vs. Cmpd 6 [0.1 µM]	0.9451	ns
			Cmpd 6: Ctr vs. Cmpd 6 [1 µM]	0.9451	ns
			Cmpd 6: Ctr vs. Cmpd 6 [10 µM]	0.9451	ns
			Cmpd 6: Ctr vs. Cmpd 6 [30 µM]	0.9451	ns
			Cmpd 6: Ctr vs. Cmpd 6 [100 µM]	0.1287	ns
	ANOVA	4	Cmpd 7: Ctr vs. Cmpd 7 [0.01 µM]	0.9989	ns
			Cmpd 7: Ctr vs. Cmpd 7 [0.1 µM]	0.9989	ns
			Cmpd 7: Ctr vs. Cmpd 7 [1 µM]	0.9989	ns
			Cmpd 7: Ctr vs. Cmpd 7 [10 µM]	0.9989	ns
			Cmpd 7: Ctr vs. Cmpd 7 [30 µM]	0.6692	ns
			Cmpd 7: Ctr vs. Cmpd 7 [100 µM]	0.0026	**
	ANOVA	4	Cmpd 8: Ctr vs. Cmpd 8 [0.01 µM]	0.9615	ns
			Cmpd 8: Ctr vs. Cmpd 8 [0.1 µM]	0.9676	ns

Figure	Test	N	Comparison	P value	Summary
			Cmpd 8: Ctr vs. Cmpd 8 [1 µM]	0.9676	ns
			Cmpd 8: Ctr vs. Cmpd 8 [10 µM]	0.6402	ns
			Cmpd 8: Ctr vs. Cmpd 8 [30 µM]	0.0322	*
			Cmpd 8: Ctr vs. Cmpd 8 [100 µM]	<0.0001	****
	ANOVA	4	topobexin (9): Ctr vs. topobexin (9) [0.01 µM]	0.9983	ns
			topobexin (9): Ctr vs. topobexin (9) [0.1 µM]	0.9983	ns
			topobexin (9): Ctr vs. topobexin (9) [1 µM]	0.9642	ns
			topobexin (9): Ctr vs. topobexin (9) [10 µM]	0.9983	ns
			topobexin (9): Ctr vs. topobexin (9) [30 µM]	0.9983	ns
			topobexin (9): Ctr vs. topobexin (9) [100 µM]	0.9642	ns
Supp 8a	ANOVA	4	DAU [1.2 µM] vs. dexrazoxane [0.1 µM] + DAU [1.2 µM]	0.2188	ns
			DAU [1.2 µM] vs. dexrazoxane [1 µM] + DAU [1.2 µM]	0.0023	**
			DAU [1.2 µM] vs. dexrazoxane [10 µM] + DAU [1.2 µM]	0.0007	***
			DAU [1.2 µM] vs. dexrazoxane [100 µM] + DAU [1.2 µM]	<0.0001	****
			DAU [1.2 µM] vs. topobexin (9) [0.1 µM] + DAU [1.2 µM]	0,0004	***
			DAU [1.2 µM] vs. topobexin (9) [1 µM] + DAU [1.2 µM]	<0.0001	****
			DAU [1.2 µM] vs. topobexin (9) [10 µM] + DAU [1.2 µM]	<0.0001	****
			DAU [1.2 µM] vs. topobexin (9) [100 µM] + DAU [1.2 µM]	<0.0001	****
	Ratio paired t-test, two-tailed	4	dexrazoxane [0.1 µM] + DAU [1.2 µM] vs. topobexin (9) [0.1 µM] + DAU [1.2 µM]	0.0356	*
			dexrazoxane [1 µM] + DAU [1.2 µM] vs. topobexin (9) [1 µM] + DAU [1.2 µM]	0.0317	*
			dexrazoxane [10 µM] + DAU [1.2 µM] vs. topobexin (9) [10 µM] + DAU [1.2 µM]	0.0013	**
			dexrazoxane [100 µM] + DAU [1.2 µM] vs. topobexin (9) [100 µM] + DAU [1.2 µM]	0.0075	**
Supp 8c	ANOVA	4	Ctr vs. topobexin (9) [0.01 µM]	0.8162	ns
			Ctr vs. topobexin (9) [0.1 µM]	0.8616	ns
			Ctr vs. topobexin (9) [1 µM]	0.1626	ns
			Ctr vs. topobexin (9) [10 µM]	<0.0001	****
			Ctr vs. topobexin (9) [30 µM]	<0.0001	****

Figure	Test	N	Comparison	P value	Summary
			Ctr vs. topobexin (9) [100 µM]	<0.0001	****
			Ctr vs. DAU [1.2 µM]	<0.0001	****
			Ctr vs. topobexin (9) [0.1 µM] + DAU [1.2 µM]	<0.0001	****
			Ctr vs. topobexin (9) [1 µM] + DAU [1.2 µM]	<0.0001	****
			Ctr vs. topobexin (9) [10 µM] + DAU [1.2 µM]	<0.0001	****
			Ctr vs. topobexin (9) [30 µM] + DAU [1.2 µM]	<0.0001	****
			Ctr vs. topobexin (9) [100 µM] + DAU [1.2 µM]	<0.0001	****
	ANOVA	4	DAU [1.2 µM] vs. topobexin (9) [0.1 µM] + DAU [1.2 µM]	0.8454	ns
			DAU [1.2 µM] vs. topobexin (9) [1 µM] + DAU [1.2 µM]	0.8454	ns
			DAU [1.2 µM] vs. topobexin (9) [10 µM] + DAU [1.2 µM]	0.0026	**
			DAU [1.2 µM] vs. topobexin (9) [30 µM] + DAU [1.2 µM]	0.0002	***
			DAU [1.2 µM] vs. topobexin (9) [100 µM] + DAU [1.2 µM]	<0.0001	****
Supp 8d	ANOVA	4	Ctr vs. topobexin (9) [0.1 µM]	0.9953	ns
			Ctr vs. topobexin (9) [0.3 µM]	0.9953	ns
			Ctr vs. topobexin (9) [1 µM]	0.9953	ns
			Ctr vs. topobexin (9) [3 µM]	0.9953	ns
			Ctr vs. topobexin (9) [10 µM]	0.9953	ns
			Ctr vs. DAU [1.2 µM]	<0.0001	****
			Ctr vs. topobexin (9) [0.1 µM] + DAU [1.2 µM]	<0.0001	****
			Ctr vs. topobexin (9) [0.3 µM] + DAU [1.2 µM]	0.0011	**
			Ctr vs. topobexin (9) [1 µM] + DAU [1.2 µM]	0.0148	*
			Ctr vs. topobexin (9) [3 µM] + DAU [1.2 µM]	0.0148	*
			Ctr vs. topobexin (9) [10 µM] + DAU [1.2 µM]	0.9102	ns
			DAU [1.2 µM] vs. topobexin (9) [0.1 µM] + DAU [1.2 µM]	0.334	ns
			DAU [1.2 µM] vs. topobexin (9) [0.3 µM] + DAU [1.2 µM]	0.1361	ns
			DAU [1.2 µM] vs. topobexin (9) [1 µM] + DAU [1.2 µM]	0.0582	ns
			DAU [1.2 µM] vs. topobexin (9) [3 µM] + DAU [1.2 µM]	0.0582	ns
			DAU [1.2 µM] vs. topobexin (9) [10 µM] + DAU [1.2 µM]	0.0013	**

Supplementary Methods – Chemistry (Synthetic procedures and characterization of compounds)

The prepared compounds were characterized using 1H NMR and 13C NMR spectroscopy. The purities of the prepared compounds were determined using elemental analysis or HPLC-HRMS experiments. All chemicals used in the syntheses were obtained from Sigma-Aldrich (Schnelldorf, Germany) and PENTA s.r.o. (Prague, Czech Republic) and were used as received. TLC separations were performed on Merck aluminum plates with silica gel 60 F254. Merck Kieselgel 60 (0.040-0.063 mm) was used for column chromatography. Melting points were recorded with a Büchi B-545 apparatus (BUCHI Labortechnik AG, Flawil, Switzerland) and are uncorrected. 1H and 13C NMR spectra were recorded using Varian Mercury Vx BB 300, VNMR S500 NMR (Varian, Palo Alto, CA, USA) or Jeol JNM-ECZ600R (JEOL Ltd., Akishima, Tokyo, Japan) spectrometers. Chemical shifts are reported as δ values in parts per million (ppm) and were indirectly referenced to tetramethylsilane (TMS) via the solvent signal. Elemental analyses were performed on an Automatic Microanalyzer EA1110CE (Fisons Instruments S.p.A., Milano, Italy). UHPLC system Acquity UPLC I-class (Waters, Millford, USA) coupled to high resolution mass spectrometer (HRMS) Synapt G2Si (Waters, Manchester, UK) based on Q-TOF were used for HRMS spectra measurement.

Scheme 1. Synthesis of BNS-22 (**1c**) and its analog **1d**

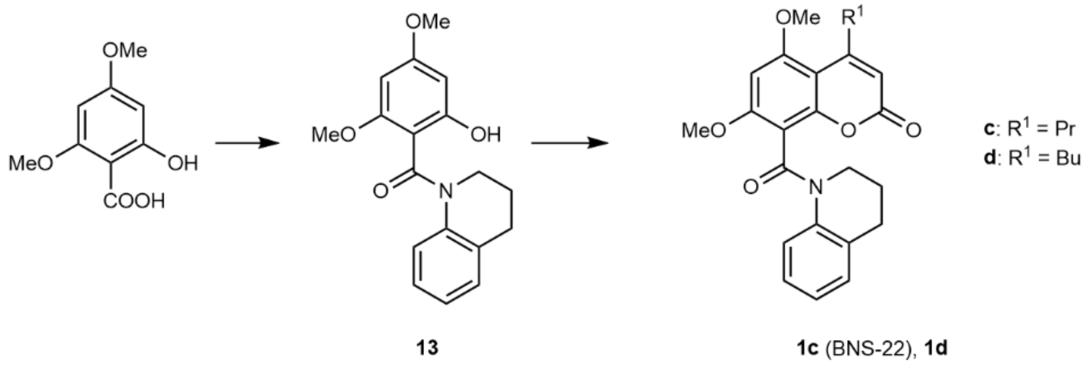

General Method 1. A mixture of *N*-(2-hydroxy-4,6-dimethoxy)benzoyl-1,2,3,4-tetrahydroquinoline **13** (0.7 g, 2.23 mmol), corresponding ethyl ester of 3-oxocarboxylic acid (2.34 mmol) and methane sulfonic acid (7 ml) was stirred for 24 h at RT. The reaction mixture was poured into ice water (150 ml) and stirred for 30 min. The aqueous layer was extracted with EtOAc (2 × 100 ml), combined organic layer was dried over anhydrous sodium sulfate, and concentrated under reduced pressure. The product was purified by column chromatography (mobile phase: hexan/EtOAc, 2:1 or hexan/EtOAc/acetic acid, 20:20:1).

*4-Butyl-5,7-dimethoxy-8-(1,2,3,4-tetrahydroquinolin-1-carbonyl)-2H-chromen-2-one (**1d**)*: The product was prepared according to General Method 1. Yield: 46 % as a beige solid; mp 129-131 °C as a dynamic equilibrium of *cis-trans* amide bond rotamers distinguishable by NMR in a ratio 2.2:1. ^{1}H NMR (500 MHz, DMSO) δ 8.08 (1H, d, *J*=8.6 Hz, CH, **B**), 7.22-7.15 (2H, m, CH, **B**), 7.13-7.05 (1H, m, CH, **A**, 1H, m, CH, **B**), 6.91 (1H, t, *J*=7.3 Hz, CH, **A**), 6.77-6.70 (1H, m, CH, **A**), 6.56-6.50 (2H, m, CH, **A**, 1H, m, CH, **B**), 6.05 (1H, s, CH, **B**), 5.89 (1H, s, CH, **A**), 4.05-3.97 (1H, m, CH_2, **A**), 4.01 (3H, s, CH_3, **B**), 3.96 (3H, s, CH_3, **B**), 3.91 (3H, s, CH_3, **A**), 3.80 (3H, s, CH_3, **A**), 3.73-3.64 (1H, m, CH_2, **A**), 3.46-3.40 (2H, m, CH_2, **B**), 2.94-2,67 (4H, m, CH_2, **A**, 4H, m, CH_2, **B**), 2,16-2.05 (1H, m, CH_2, **A**), 2.00-1.82 (1H, m, CH_2, **A**,2H, m, CH_2, **B**), 1.57-1.12 (4H, m, CH_2, **A**, 4H, m, CH_2, **B**), 0.95 (3H, t, *J*=7.3 Hz, CH_3, **B**), 0.88 (3H, t, *J*=7.1 Hz, CH_3, **A**). ^{13}C NMR (125 MHz, DMSO) **isomer A**: δ 163.5, 159.3, 159.2, 158.9, 158.0, 152.1, 138.6, 133.2, 128.0, 125.3, 125.3, 122.8, 110.3, 108.1, 102.5, 92.5, 56.6, 56.5, 42.5, 35.6, 31.5, 26.1, 23.5, 22.2,

13.9. ^{13}C NMR (125 MHz, DMSO) **isomer B**: δ 163.3, 15932, 159.1, 158.4, 158.3, 151.8, 137.7, 129.8, 129.3, 125.5, 124.6, 124.1, 110.7, 107.9, 103.1, 92.9, 56.8, 56.7, 46.5, 35.8, 31.7, 26.6, 23.3, 22.4, 14.0. HRMS (m/z): $[M+H]^+$ calcd. for $C_{25}H_{28}NO_5$, 422.1962; found, 422.1963; Elem. Anal. Calcd for $C_{25}H_{27}NO_5$: C, 71.24; H, 6.46; N, 3.32. Found: C, 71.62; H, 6.83; N, 3.98.

Scheme 2. Synthesis of compounds **2a-d**.

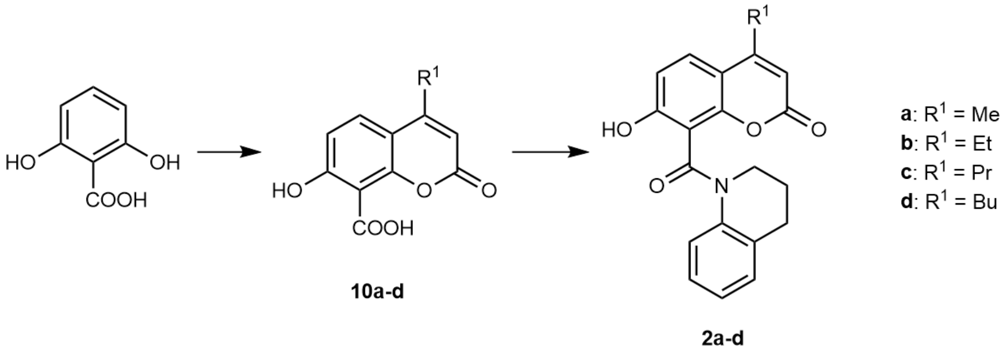

General Method 2. Synthesis of carboxylic acids 10a-d. Methyl or ethyl ester of corresponding aliphatic 3-oxocarboxylic acid (14.6 mmol) was added dropwise to a suspension of 2,6-dihydroxybenzoic acid (1.5 g, 9.73 mmol) in conc. H_2SO_4 (4.6 g, 2.5 mL, 45 mmol). The reaction mixture was heated to 75 °C for 2-5 h. After cooling to RT, the reaction mixture was poured into ice water (150 mL) and a precipitation was filtered off, washed with water to neutral pH and dried over P_2O_5.

*7-Hydroxy-4-methyl-2-oxo-2H-chromene-8-carboxylic acid (**10a**)*: The product was prepared according to General Method 2. Ethyl ester of 3-oxobutyric acid was used as a substrate. The reaction mixture was heated to 75 °C for 2 h. Yield: 70% as a white solid; mp 255-257 °C. ^1H NMR (500 MHz, DMSO) δ 7.66 (d, J = 8.7 Hz, 1H), 6.90 (d, J = 8.8 Hz, 1H), 6.18 (d, J = 1.3 Hz, 1H), 2.37 (d, J = 1.2 Hz, 3H). ^{13}C NMR (126 MHz, DMSO) δ 166.26, 159.81, 158.56, 153.86, 151.42, 127.58, 112.93, 112.03, 110.76, 110.70, 18.49. HRMS (m/z): $[M+H]^+$ calcd. for $C_{11}H_9O_5$, 221.0444; found, 221.0450; Elem. Anal. Calcd. for $C_{11}H_8O_5$: C, 60.01; H, 3.66. Found: C, 59.62; H, 3.82.

*4-Ethyl-7-hydroxy-2-oxo-2H-chromene-8-carboxylic acid (**10b**)*: The product was prepared according to General Method 2. Ethyl ester of 3-oxovaleric acid was used as a substrate. The reaction mixture was heated to 75 °C for 2 h. Yield: 61% as a white solid; mp 234-236 °C. ^1H NMR (500 MHz, DMSO) δ 11.05 (s, 1H), 7.70 (d, J = 8.8 Hz, 1H), 6.90 (d, J = 8.8 Hz, 1H), 6.13 (d, J = 1.4 Hz, 1H), 2.77 (qd, J = 7.4, 1.2 Hz, 2H), 1.20 (t, J = 7.4 Hz, 3H). ^{13}C NMR (126 MHz, DMSO) δ 166.16, 160.00, 158.59, 158.34, 151.51, 127.06, 112.88, 111.12, 110.92, 108.88, 24.35, 12.54. HRMS (m/z): $[M+H]^+$ calcd. for $C_{12}H_{11}O_5$, 235.0601; found, 235.0609; Elem. Anal. Calcd. for $C_{12}H_{10}O_5$: C, 61.54; H, 4.30. Found: C, 61.16; H, 4.14.

*7-Hydroxy-4-propyl-2-oxo-2H-chromene-8-carboxylic acid (**10c**):* The product was prepared according to General Method 2. Ethyl ester of 3-oxohexanoic acid was used as a substrate. The reaction mixture was heated to 75 °C for 5 h. Yield: 51% as a white solid; mp 210-212 °C. ^1H NMR (500 MHz, DMSO) δ 7.71 (d, J = 8.8 Hz, 1H), 6.90 (d, J = 8.8 Hz, 1H), 6.13 (s, 1H), 2.71 (t, J = 7.6 Hz, 2H), 1.61 (h, J = 7.4 Hz, 2H), 0.95 (t, J = 7.3 Hz, 3H). ^{13}C NMR (126 MHz, DMSO) δ 166.13, 159.85, 158.36, 157.11, 151.63, 127.24,

112.94, 111.16, 110.96, 109.83, 33.11, 21.56, 13.81. HRMS (m/z): $[M+H]^+$ calcd. for $C_{13}H_{13}O_5$, 249.0757; found, 249.0765; Elem. Anal. Calcd. for $C_{13}H_{12}O_5$: C, 62.9; H, 4.87. Found: C, 62.92; H, 4.81.

*4-Butyl-7-hydroxy-2-oxo-2H-chromene-8-carboxylic acid (**10d**)*: The product was prepared according to General Method 2. Methyl ester of 3-oxoheptanoic acid was used as a substrate. The reaction mixture was heated to 75 °C for 3 h. Yield: 73% as a white solid; mp 179-181 °C. ^1H NMR (600 MHz, DMSO-D_6) δ 7.67 (d, J = 8.9 Hz, 1H), 6.87 (d, J = 8.8 Hz, 1H), 6.10 (s, 1H), 2.73 – 2.64 (m, 2H), 1.58 – 1.48 (m, 2H), 1.37 – 1.31 (m, 2H), 0.87 (t, J = 7.4 Hz, 3H). ^{13}C NMR (151 MHz, DMSO-D_6) δ 166.50, 160.22, 158.77, 157.73, 152.01, 127.58, 113.33, 111.48, 111.30, 110.09, 31.30, 30.75, 22.43, 14.20. HRMS (m/z): $[M+H]^+$ calcd. for $C_{14}H_{15}O_5$, 263.0914; found, 263.0923; Elem. Anal. Calcd. for $C_{14}H_{14}O_5$: C, 64.12; H, 5.38. Found: C, 64.08; H, 5.48.

General Method 3. Synthesis of compounds 2a-d. To a solution of corresponding carboxylic acid **10a-d** (4.27 mmol) and 1,2,3,4-tetrahydroquinoline (2.16 g, 2.04 mL, 16.2 mmol) in CH_2Cl_2 (50 mL) EDC.HCl (1.88 g, 9.83 mmol) and DMAP (26 mg, 0.214 mmol) were added. The reaction mixture was stirred at room temperature (RT) for 48-72 h. Then, the reaction mixture was washed with water (2 × 50 mL), 1M HCl (2 × 50 mL), and brine (1 × 50 mL). Organic phase was dried over anhydrous sodium sulfate and concentrated under reduced pressure. The product was purified by column chromatography (mobile phase: hexane/EtOAc/acetic acid, 20:20:1).

*7-Hydroxy-4-methyl-8-(1,2,3,4-tetrahydroquinoline-1-carbonyl)-2H-chromen-2-one (**2a**)*: The product was prepared according to General Method 3 using substrate **10a**. The reaction mixture was stirred at RT for 48 h. Yield: 64% as a white solid; mp 241-243 °C as a dynamic equilibrium of *cis-trans* amide bond rotamers distinguishable by NMR in a ratio 2.2:1. ^1H NMR (500 MHz, DMSO) δ 11.02 (1H, bs, OH, **A**, 1H, bs, OH, **B**), 8.11 (1H, d, J = 8.3 Hz, CH, **B**), 7.67 (1H, d, J = 8.3 Hz, CH, **B**), 7.50 (1H, d, J = 8.8 Hz, CH, **A**), 7.23-7.18 (2H, m, CH, **B**), 7.13-7.06 (1H, m, CH, **A**, 1H, m, CH, **B**), 6.95 (1H, d, J = 8.8 Hz, CH, **B**), 6.90 (1H, t, J = 7.3 Hz, CH, **A**), 6.76 (1H, d, J = 8.8 Hz, CH, **A**), 6.73 (1H, t, J = 7.3 Hz, CH, **A**), 6.66 (1H, d, J = 7.3 Hz, CH, **A**), 6.19 (1H, s, CH, **B**), 6.03 (1H, s, CH, **A**), 4.14-4.04 (1H, m, CH_2, **A**), 3.71-3.61 (1H, m, CH_2, **A**), 3.50-3.42 (2H, m, CH_2, **B**), 2.91-2.68 (2H, m, CH_2, **A**, 2H, m, CH_2, **B**), 2.40 (3H, s, CH_3, **B**), 2.28 (3H, s, CH_3, **A**), 2.20-2.09 (1H, m, CH_2, **A**), 1.99-1.81 (1H, m, CH_2, **A**, 2H, m, CH_2, **B**). **Isomer A**: ^{13}C NMR (125 MHz, DMSO) δ 163.8, 159.5, 158.2, 153.7, 150.8, 138.5, 133.3, 128.1, 126.7, 125.4, 125.3, 122.8, 113.5, 112.5, 111.5, 110.3, 42.5, 26.2, 23.6, 18.3. **Isomer B**: ^{13}C NMR (125 MHz, DMSO) δ 163.6, 159.9, 157.2, 153.9, 150.7, 137.7, 129.8, 129.3, 126.7, 125.5, 124.6, 124.2, 113.2, 112.9, 112.2, 110.7, 46.5, 26.6, 23.4, 18.4. HRMS (m/z): $[M+H]^+$ calcd. for $C_{20}H_{18}NO_4$, 336.1230; found, 336.1240; Elem. Anal. Calcd for $C_{20}H_{17}NO_4$: C, 71.63; H, 5.11; N, 4.18. Found: C, 71.38; H, 5.18; N, 4.31.

*4-Ethyl-7-hydroxy-8-(1,2,3,4-tetrahydroquinolin-1-carbonyl)-2H-chromen-2-one (**2b**):* The product was prepared according to General Method 3 using substrate **10b**. The reaction mixture was stirred at RT for 72 h Yield: 64% as a white solid; mp 218-219 °C as a dynamic equilibrium of *cis-trans* amide bond rotamers distinguishable by NMR in a ratio 2:1. ^1H NMR (500 MHz, DMSO) δ 10.96 (1H, bs, OH, **B**), 10.87 (1H,

bs, OH, **A**), 8.11 (1H, d, *J* = 8.3 Hz, CH, **B**), 7.72 (1H, d, *J* = 8.3 Hz, CH, **B**), 7.55 (1H, d, *J* = 8.8 Hz, CH, **A**), 7.23-7.17 (2H, m, CH, **B**), 7.13-7.05 (1H, m, CH, **A**, 1H, m, CH, **B**), 6.96 (1H, d, *J* = 8.8 Hz, CH, **B**), 6.90 (1H, dt, *J* = 7.6 Hz, *J* = 1.0 Hz, CH, **A**), 6.77 (1H, d, *J* = 8.8 Hz, CH, **A**), 6.73 (1H, t, *J* = 7.6 Hz, CH, **A**), 6.65 (1H, d, *J* = 7.6 Hz, CH, **A**), 6.15 (1H, s, CH, **B**) 5.98 (1H, s, CH, **A**), 4.13-4.06 (1H, m, CH_2, **A**), 3.69-3.62 (1H, m, CH_2, **A**), 3.49-3.43 (2H, m, CH_2, **B**), 2.95-2.60 (4H, m, CH_2, **A**, 4H, m, CH_2, **B**), 2.20-2.09 (1H, m, CH_2, **A**), 1.98-1,82 (1H, m, CH_2, **A**, 2H, m, CH_2, **B**), 1.23 (3H, t, *J* = 7.3 Hz, CH_3, **B**), 1.14 (3H, t, *J* = 7.3 Hz, CH_3, **A**). **Isomer A**: ^{13}C NMR (125 MHz, DMSO) δ 163.8, 159.7, 158.4, 157.9, 150.9, 138.5, 133.3, 128.0, 126.3, 125.3, 125.3, 122.7, 113.6, 112.5, 110.8, 108.4, 42.5, 26.2, 24.1, 23.5, 12.2. **Isomer B**: ^{13}C NMR (125 MHz, DMSO) δ 163.5, 160.1, 158.6, 156.9, 150.8, 137.7, 129.8, 129.3, 126.3, 125.5, 124.6, 124.1, 113.4, 112.8, 111.4, 109.0, 46.5, 26.6, 24.3, 23.4, 12.5. HRMS (m/z): $[M+H]^+$ calcd. for $C_{21}H_{20}NO_4$, 350.1387; found, 350.1396; Elem. Anal. Calcd for $C_{21}H_{19}NO_4$: C, 72.19; H, 5.48; N, 4.01. Found: C, 72.32; H, 5.39; N, 3.91.

*7-Hydroxy-4-propyl-8-(1,2,3,4-tetrahydroquinolin-1-carbonyl)-2H-chromen-2-one (**2c**)*:

The product was prepared according to General Method 3 using substrate **10c**. The reaction mixture was stirred at RT for 72 h. Yield: 64% as a white solid; mp 184-185 °C as a dynamic equilibrium of *cis-trans* amide bond rotamers distinguishable by NMR in a ratio 2:1. ^{1}H NMR (500 MHz, DMSO) δ 10.60 (1H, bs, OH, **A** , 1H, bs, OH, **B**), 8.11 (1H, d, *J* = 8.6 Hz, CH, **B**), 7.71 (1H, d, *J* = 8.6 Hz, CH, **B**), 7.55 (1H, d, *J* = 8.8 Hz, CH, **A**), 7.23-7.18 (2H, m, CH, **B**), 7.13-7.06 (1H, m, CH, **A**, 1H, m, CH, **B**), 6.94 (1H, d, *J* = 8.8 Hz, CH, **B**), 6.90 (1H, t, *J* = 7.6 Hz, CH, **A**), 6.75 (1H, d, *J* = 8.8 Hz, CH, **A**), 6.72 (1H, t, *J* = 7.6 Hz, CH, **A**), 6.65 (1H, d, *J* = 7.6 Hz, CH, **A**), 6.14 (1H, s, CH, **B**), 5.97 (1H, s, CH, **A**), 4.13-4.06 (1H, m, CH_2, **A**), 3.68-3.61 (1H, m, CH_2, **A**), 3.46 (2H, t, *J* = 5.6 Hz, CH_2, **B**), 2.91-2.52 (4H, m, CH_2, **A**, 4H, m, CH_2, **B**), 2.20-2.09 (1H, m, CH_2, **A**), 1.97-1.83 (1H, m, CH_2, **A**, 2H, m, CH_2, **B**), 1.71-1.46 (2H, m, CH_2, **A**, 2H, m, CH_2, **B**), 0.98 (3H, t, *J* = 7.3 Hz, CH_3, **B**), 0.89 (3H, t, *J* = 7.3 Hz, CH_3, **A**). **Isomer A**: ^{13}C NMR (125 MHz, DMSO) δ 163.9, 159.6, 158.1, 157.0, 151.1, 138.5, 133.3, 128.0, 126.4, 125.3, 125.3, 122.7, 113.6, 112.5, 110.7, 109.3, 42.4, 32.9, 26.2, 23.5, 21.4, 13.7. **Isomer B**: ^{13}C NMR (125 MHz, DMSO) δ 163.6, 160.0, 158.1, 157.2, 151.0, 137.7, 129.8, 129.3, 126.4, 125.5, 124.5, 124.1, 113.4, 112.9, 111.3, 109.7, 46.5, 33.1, 26.6, 23.4, 21.5, 13.9. HRMS (m/z): $[M+H]^+$ calcd. for $C_{22}H_{22}NO_4$, 364.1543; found, 364.1549; Elem. Anal. Calcd for $C_{22}H_{21}NO_4$: C, 72.71; H, 5.82; N, 3.85. Found: C, 72.33; H, 5.87; N 3.77.

*4-Butyl-7-hydroxy-8-(1,2,3,4-tetrahydroqquinolin-1-carbonyl)-2H-chromen-2-one (**2d**)*: The product was prepared according to General Method 3 using substrate **10d**. The reaction mixture was stirred at RT for 48 h. Yield: 46% as a beige solid; mp 133-135 °C as a dynamic equilibrium of *cis-trans* amide bond rotamers distinguishable by NMR in a ratio 2:1. ^{1}H NMR (500 MHz, DMSO) δ 10.98 (1H, bs, OH, **A**, 1H, bs, OH, **B**), 8.11 (1H, d, *J* = 8.3 Hz, CH, **B**), 7.72 (1H, d, *J* = 8.3 Hz, CH, **B**), 7.56 (1H, d, *J* = 8.7 Hz, CH, **A**), 7.23-7.18 (2H, m, CH, **B**), 7.13-7.05 (1H, m, CH, **A**, 1H, m, CH, **B**), 6.95 (1H, d, *J* = 8.7 Hz, CH, **B**), 6.90 (1H, t, *J* = 7.7 Hz, CH, **A**), 6.76 (1H, d, *J* = 8.7 Hz, CH, **A**), 6.72 (1H, t, *J* = 7.7 Hz, CH, **A**), 6.64 (1H, d, *J* = 7.7 Hz, CH, **A**), 6.14 (1H, s, CH, **B**), 5.97 (1H, s, CH, **A**), 4.14-4.04 (1H, m, CH_2, **A**), 3.69-3.59 (1H, m, CH_2, **A**), 3.48-3.44 (2H, m, CH_2, **B**), 2.90-2.55 (4H, m, CH_2, **A**, 4H, m, CH_2, **B**), 2.20-2.09 (1H, m, CH_2, **A**), 1.98-1.83 (1H, m, CH_2, **A**, 2H, m, CH_2, **B**), 1.65-1.55 (2H, m, CH_2, **B**), 1.55-1.45 (2H, m, CH_2, **A**), 1.45-1.37

(2H, m, CH_2, **B**), 1.36-1.25 (2H, m, CH_2, **A**) 0.93 (3H, t, *J* = 7.5 Hz, CH_3, **B**), 0.87 (3H, t, *J* = 7.3 Hz, CH_3, **A**). **Isomer A**: ^{13}C NMR (125 MHz, DMSO) δ 163.9, 159.6, 158.0, 157.3, 151.1, 138.5, 133.3, 128.0, 126.5, 125.3, 125.3, 122.8, 113.7, 112.5, 110.7, 109.3, 42.5, 30.8, 30.2, 26.2, 23.6, 20.0, 13.9. **Isomer B**: ^{13}C NMR (125 MHz, DMSO) δ 163.6, 160.0, 157.5, 157.1, 151.0, 137.7, 129.8, 129.3, 126.5, 125.5, 124.6, 124.1, 113.5, 112.9, 111.4, 109.7, 46.5, 31.0, 30.4, 26.6, 23.4, 22.2, 13.9. HRMS (m/z): $[M+H]^+$ calcd. for $C_{23}H_{24}NO_4$, 378.1700; found, 378.1711; Elem. Anal. Calcd for $C_{23}H_{23}NO_4$: C, 73.19; H, 6.14; N, 3.71. Found: C, 72.81; H 6.05; N 3.62.

Scheme 3. Synthesis of compounds **3a-d, 4a-d, and 5a-d.**

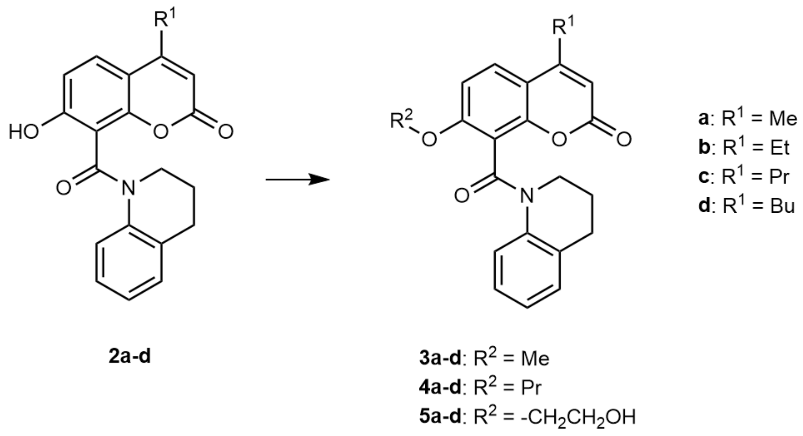

General Method 4. Synthesis of compounds 3a-d. The mixture of corresponding 4-alkyl-7-hydroxy-8-(1,2,3,4-tetrahydroquinoline-1-carbonyl)-2*H*-chromen-2-one **2a-d** (0.9 mmol), dimethyl sulfate (0.23 g, 0.17 mL, 1.8 mmol) and potassium carbonate (0.25 g, 1.8 mmol) in DMF (10 mL) was heated to 100 ºC for 3-6 h. Then, volatiles were evaporated under reduced pressure. The residue was dissolved in EtOAc (50 mL) and washed with water (2 × 40 mL) and brine (2 × 30 mL). The organic phase was dried over anhydrous sodium sulfate and concentrated under reduced pressure. Product was suspended with EtOAc (5 mL), filtered off and obtained in high quality without additional purification.

*7-Methoxy-4-methyl-8-(1,2,3,4-tetrahydroquinolin-1-carbonyl)-2H-chromen-2-one (**3a**):* The product was prepared according to General Method 4. Compound **2a** was used as starting material. The reaction mixture was heated to reflux for 3 h. Yield: 97% as a beige solid; mp 170-171 °C as a dynamic equilibrium of *cis-trans* amide bond rotamers distinguishable by NMR in a ratio 2.5:1. ^{1}H NMR (500 MHz, DMSO) δ 8.12-8.08 (1H, m, CH, **B**), 7.84 (1H, d, *J*=8.8 Hz, CH, **B**), 7.67 (1H, d, *J* = 8.8 Hz, CH, **A**), 7.23-7.18 (3H, m, CH, **B**), 7.14-7.07 (1H, m, CH, **A**, 1H, m, CH, **B**), 7.00 (1H, d, *J* = 8.8 Hz, CH, **A**), 6.91 (1H, dt, *J* = 7.5 Hz, *J* = 1.0 Hz, CH, **A**), 6.70 (1H, t, *J* = 7.5 Hz, CH, **A**), 6.53 (1H, d, *J* = 7.5 Hz, CH, **A**), 6.28 (1H, s, CH, **B**), 6.13 (1H, s, CH, **A**), 4.05-3.96 (1H, m, CH_2, **A**), 3.93 (3H, s, CH_3, **B**), 3.77 (3H, s, CH_3, **A**), 3.78-3.70 (1H, m, CH_2, **A**), 3.45-3.38 (2H, m, CH_2, **B**), 2.91-2.78 (1H, m, CH_2, **A**, 2H, m, CH_2, **B**), 2.78-2.69 (1H, m, CH_2, **A**), 2.44 (3H, s CH_3, **B**), 2.33 (3H, s CH_3, **A**), 2.16-2.06 (1H, m, CH_2, **A**), 2.00-1.82 (1H, m, CH_2, **A**, 2H, m, CH_2, **B**). **Isomer A**: ^{13}C NMR (125 MHz, DMSO) δ 163.3, 159.3, 158.6, 153.5, 150.1, 138.3, 133.4,

128.0, 127.3, 125.5, 125.4, 122.8, 114.6, 113.2, 111.5, 108.2, 56.5, 42.5, 26.1, 23.5, 18.3. **Isomer B**: ^{13}C NMR (125 MHz, DMSO) δ 163.0, 159.6, 157.9, 153.7, 149.9, 137.5, 129.9, 129.4, 127.2, 125.6, 124.7, 124.1, 114.4, 113.9, 111.9, 108.5, 56.8, 46.5, 26.5, 23.3, 18.4. HRMS (m/z): $[M+H]^+$ calcd. for $C_{21}H_{20}NO_4$, 350.1387; found, 350.1394; Elem. Anal. Calcd for $C_{21}H_{19}NO_4$: C, 72.19; H, 5.48; N, 4.01. Found: C, 72.58; H, 5.49; N, 3.89.

*4-Ethyl-7-methoxy-8-(1,2,3,4-tetrahydroquinolin-1-carbonyl)-2H-chromen-2-one (**3b**):* The product was prepared according to General Method 4. Compound **2b** was used as starting material. The reaction mixture was heated to reflux for 3 h. Yield: 86 % as a white solid; mp 218-220 °C as a dynamic equilibrium of *cis-trans* amide bond rotamers distinguishable by NMR in a ratio 2.4:1. ^{1}H NMR (500 MHz, DMSO) δ 8.10 (1H, d, *J* = 8.6 Hz, CH, **B**), 7.89 (1H, d, *J* = 8.6 Hz, CH, **B**), 7.72 (1H, d, *J* = 8.8 Hz, CH, **A**), 7.24-7.18 (3H, m, CH, **B**), 7.14-7.06 (1H, m, CH, **A**, 1H, m, CH, **B**), 6.99 (1H, d, *J* = 8.8 Hz, CH, **A**), 6.91 (1H, t, *J* = 7.4 Hz, CH, **A**), 6.70 (1H, t, *J* = 7.4 Hz, CH, **A**), 6.53 (1H, d, *J* = 7.4 Hz, CH, **A**), 6.22 (1H, s, CH, **B**), 6.07 (1H, s, CH, **A**), 4.06-3.96 (1H, m, CH_2, **A**), 3.93 (3H, s, CH_3, **B**), 3.77 (3H, s, CH_3, **A**), 3.80-3.69 (1H, m, CH_2, **A**), 3.46-3.37 (2H, m, CH_2, **B**), 2.92-2.66 (4H, m, CH_2, **A**, 4H, m, CH_2, **B**), 2.16-2.06 (1H, m, CH_2, **A**), 2.01-1.83 (1H, m, CH_2, **A**, 2H, m, CH_2, **B**), 1.24 (3H, t, *J* = 7.4 Hz, CH_3, **B**), 1.15 (3H, t, *J* = 7.2 Hz, CH_3, **A**). **Isomer A**: ^{13}C NMR (125 MHz, DMSO) δ 163.3, 159.6, 158.5, 158.2, 150.3, 138.3, 133.4, 128.0, 126.8, 125.5, 125.4, 122.8, 114.8, 112.4, 109.6, 108.2, 56.5, 42.5, 26.1, 24.1, 23.5, 12.2. **Isomer B**: ^{13}C NMR (125 MHz, DMSO) δ 163.0, 159.9, 158.4, 157.7, 150.0, 137.5, 129.9, 129.4, 126.8, 125.6, 124.7, 124.1, 114.5, 113.0, 110.1, 108.6, 56.8, 46.6, 26.5, 24.3, 23.3, 12.5. HRMS (m/z): $[M+H]^+$ calcd. for $C_{22}H_{22}NO_4$, 364.1543; found, 364.1549; Elem. Anal. Calcd for $C_{22}H_{21}NO_4$: C, 72.71; H, 5.82; N, 3.85. Found: C, 72.32; H, 5.89; N, 3.69.

*7-Methoxy-4-propyl-8-(1,2,3,4-tetrahydroquinolin-1-carbonyl)-2H-chromen-2-one (**3c**):* The product was prepared according to General Method 4. Compound **2c** was used as starting material. The reaction mixture was heated to reflux for 6 h. Yield: 80% as a white solid; mp 200-202 °C as a dynamic equilibrium of *cis-trans* amide bond rotamers distinguishable by NMR in a ratio 2.4:1. ^{1}H NMR (500 MHz, DMSO) δ 8.10 (1H, d, *J* = 8.2 Hz, CH, **B**), 7.90 (1H, d, *J* = 8.2 Hz, CH, **B**), 7.73 (1H, d, *J* = 8.9 Hz, CH, **A**), 7.23-7.18 (3H, m, CH, **B**), 7.14-7.07 (1H, m, CH, **A**, 1H, m, CH, **B**), 6.99 (1H, d, *J* = 8.9 Hz, CH, **A**), 6.90 (1H, dt, *J* = 7.3 Hz, *J* = 1.4 Hz, CH, **A**), 6.69 (1H, t, *J* = 7.3 Hz, CH, **A**), 6.52 (1H, d, *J* = 7.3 Hz, CH, **A**), 6.23 (1H, s, CH, **B**), 6.07 (1H, s, CH, **A**), 4.06-3.97 (1H, m, CH_2, **A**), 3.93 (3H, s, CH_3, **B**), 3.77 (3H, s, CH_3, **A**), 3.78-3.70 (1H, m, CH_2, **A**), 3.46-3.39 (2H, m, CH_2, **B**), 2.91-2.59 (4H, m, CH_2, **A**, 4H, m, CH_2, **B**), 2.16-2.06 (1H, m, CH_2, **A**), 2.00-1.84 (1H, m, CH_2, **A**, 2H, m, CH_2, **B**), 1.75-1.49 (2H, m, CH_2, **A**, 2H, m, CH_2, **B**), 0.99 (3H, t, *J* = 7.3 Hz, CH_3, **B**), 0.91 (3H, t, *J* = 7.5 Hz, CH_3, **A**). **Isomer A**: ^{13}C NMR (125 MHz, DMSO) δ 163.3, 159.4, 158.5, 156.8, 150.4, 138.3, 133.4, 128.0, 127.0, 125.5, 125.3, 122.8, 114.8, 112.4, 110.6, 108.2, 56.5, 42.5, 32.8, 26.1, 24.5, 21.3, 13.7. **Isomer B**: ^{13}C NMR (125 MHz, DMSO) δ 163.0, 159.7, 157.7, 156.9, 150.1, 137.5, 129.9, 129.4, 127.0, 125.6, 124.7, 124.1, 114.6, 113.1, 111.0, 108.6, 56.8, 46.5, 33.0, 26.5, 24.3, 21.5, 13.9. HRMS (m/z): $[M+H]^+$ calcd. for $C_{23}H_{24}NO_4$, 378.1700; found, 378.1711; Elem. Anal. Calcd for $C_{23}H_{23}NO_4$: C, 73.19; H, 6.14; N, 3.71. Found: C, 72.8; H, 6.10; N, 3.58.

*4-Butyl-7-methoxy-8-(1,2,3,4-tetrahydroquinolin-1-carbonyl)-2H-chromen-2-one (**3d**)*: The product was prepared according to General Method 4. Compound **2d** was used as starting material. The reaction mixture

was refluxed for 6 h. Yield: 63 % as a beige solid; mp 161-163 °C as a dynamic equilibrium of *cis-trans* amide bond rotamers distinguishable by NMR in a ratio 2.9:1. ^{1}H NMR (600 MHz, DMSO) δ 8.07 (1H, d, *J* = 8.1 Hz, CH, **B**), 7.85 (1H, d, *J* = 8.1 Hz, CH, **B**), 7.69 (1H, d, *J* = 9.0 Hz, CH, **A**), 7.20-7.14 (3H, m, CH, **B**), 7.11-7.02 (1H, m, CH, **A**, 1H, m, CH, **B**), 6.95 (1H, d, *J* = 9.0 Hz, CH, **A**), 6.87 (1H, t, *J* = 7.4 Hz, CH, **A**), 6.66 (1H, t, *J*=7.4 Hz, CH, **A**), 6.48 (1H, d, *J* = 7.4 Hz, CH, **A**), 6.19 (1H, s, CH, **B**), 6.04 (1H, s, CH, **A**), 4.03-3.94 (1H, m, CH_2, **A**), 3.90 (3H, s, CH_3, **B**), 3.74 (3H, s, CH_3, **A**), 3.73-3.67 (1H, m, CH_2, **A**), 3.42-3.36 (2H, m, CH_2, **B**), 2.88-2.57 (4H, m, CH_2, **A**, 4H, m, CH_2, **B**), 2.12-2.03 (1H, m, CH_2, **A**), 1.94-1.87 (1H, m, CH_2, **A**), 1.87-1.80 (2H, m, CH_2, **B**), 1.63-1.53 (2H, m, CH_2, **B**), 1.53-1.42 (2H, m, CH_2, **A**), 1.41-1.35 (2H, m, CH_2, **B**), 1.33-1.25 (2H, m, CH_2, **A**), 0.90 (3H, t, *J* = 7.2 Hz, CH_3, **B**), 0.85 (3H, t, *J* = 7.5 Hz, CH_3, **A**). **Isomer A**: ^{13}C NMR (150 MHz, DMSO) δ 163.3, 159.4, 158.5, 157.0, 150.4, 138.3, 133.4, 128.0, 127.0, 125.4, 125.3, 122.8, 114.8, 112.4, 110.5, 108.2, 56.5, 42.5, 30.7, 30.1, 26.1, 23.5, 21.9, 13.8. **Isomer B**: ^{13}C NMR (150 MHz, DMSO) δ 163.0, 159.7, 157.7, 157.2, 150.1, 137.5, 129.8, 129.3, 126.9, 125.6, 124.7, 124.1, 114.6, 113.0, 110.9, 108.6, 56.7, 46.5, 30.9, 30.3, 26.5, 23.3, 22.1, 13.9. HRMS (m/z): $[M+H]^+$ calcd. for $C_{24}H_{26}NO_4$, 392.1856; found, 392.1865; Elem. Anal. Calcd for $C_{24}H_{25}NO_4$: C, 73.64; H, 6.44; N, 3.58. Found: C, 73.25; H, 6.61; N, 3.42.

General Method 5. Synthesis of compounds 4a-d. 1-Bromopropane (0.2 g, 0.15 mL, 1.63 mmol) was added to a solution of corresponding 4-alkyl-7-hydroxy-8-(1,2,3,4-tetrahydroquinoline-1-carbonyl)-2*H*-chromen-2-one **2a-d** (0.54 mmol) and potassium carbonate (0.23 g, 1.63 mmol) in DMF (7 mL). The reaction mixture was heated to 75 °C for 12 h. After cooling, the reaction mixture was diluted with EtOAc (50 mL), washed with water (3 × 30 mL) and brine (1 × 30 mL), organic phase was dried over anhydrous sodium sulfate, and concentrated under reduced pressure. The product was suspended in diethyl ether (10-15 mL), filtered off and obtained in high quality without additional purification.

*4-Methyl-7-propoxy-8-(1,2,3,4-tetrahydroquinolin-1-carbonyl)-2H-chromen-2-one (**4a**)*: The product was prepared according to General Method 5. Compound **2a** was used as starting material. Yield: 69% as a white solid; mp 158-159 °C as a dynamic equilibrium of *cis-trans* amide bond rotamers distinguishable by NMR in a ratio 2.2:1. ^{1}H NMR (500 MHz, DMSO) δ 8.06 (1H, d, *J* = 8.9 Hz, CH, **B**), 7.80 (1H, d, *J* = 8.9 Hz, CH, **B**), 7.65 (1H, d, *J* = 8.8 Hz, CH, **A**), 7.25-7.16 (3H, m, CH, **B**), 7.14-7.06 (1H, m, CH, **A**, 1H, m, CH, **B**), 6.98 (1H, d, *J* = 8.8 Hz, CH, **A**), 6.91 (1H, t, *J* = 7.6 Hz CH, **A**), 6.70 (1H, t, *J* = 7.6 Hz, CH, **A**), 6.56 (1H, d, *J* = 7.6 Hz, CH, **A**), 6.27 (1H, s, CH, **B**), 6.14 (1H, s, CH, **A**), 4.16-4.10 (2H, m, CH_2, **B**), 4.04-3.97 (1H, m, CH_2, **A**), 3.97-3.82 (3H, m, CH_2, **A**), 3.51-3.42 (1H, m, CH_2, **B**), 3.41-3.34 (1H, m, CH_2, **B**), 2.92-2.83 (1H, m, CH_2, **A**), 2.83-2.69 (1H, m, CH_2, **A**, 2H, m, CH_2, **B**), 2.43 (3H, s CH_3, **B**), 2.33 (3H, s CH_3, **A**), 2.15-2.06 (1H, m CH_2, **A**), 2.00-1.82 (1H, m CH_2, **A**, 2H, m CH_2, **B**), 1.75-1.59 (2H, m CH_2, **A**, 2H, m CH_2, **B**), 0.92 (3H, t, *J* = 7.3 Hz, CH_3, **A**, 3H, t, *J* = 7.3 Hz, CH_3, **B**). **Isomer A:** ^{13}C NMR (125 MHz, DMSO) δ 163.3, 159.4, 157.9, 153.5, 150.4, 138.2, 132.9, 128.2, 127.1, 125.4, 125.4, 122.6, 114.8, 113.0, 111.5, 108.8, 70.3, 42.6, 26.3, 23.4, 22.0, 18.3, 10.3. **Isomer B:** ^{13}C NMR (125 MHz, DMSO) δ 162.1, 159.6, 157.3, 153.6, 150.0, 137.5, 129.9, 129.3, 127.1, 125.6, 124.7, 124.0, 114.5, 113.7, 111.8, 109.3, 70.4, 46.4, 26.5, 23.5, 22.1, 18.4, 10.4. HRMS (m/z): $[M+H]^+$ calcd. for $C_{23}H_{24}NO_4$, 378.1700; found, 378.1702; Elem. Anal. Calcd for $C_{23}H_{23}NO_4$: C, 73.19; H, 6.14; N, 3.71. Found: C, 72.97; H 5.94, N 3.65.

*4-Ethyl-7-propoxy-8-(1,2,3,4-tetrahydroquinolin-1-carbonyl)-2H-chromen-2-one (**4b**):* The product was prepared according to General Method 5. Compound **2b** was used as starting material. Yield: 75% as a white solid; mp 139-140 °C as a dynamic equilibrium of *cis-trans* amide bond rotamers distinguishable by NMR in a ratio 2:1. ^{1}H NMR (600 MHz, DMSO) δ 8.01 (1H, d, *J* = 8.5 Hz, CH, **B**), 7.80 (1H, d, *J* = 8.5 Hz, CH, **B**), 7.65 (1H, d, *J* = 9.0 Hz, CH, **A**), 7.20-7.12 (3H, m, CH, **B**), 7.09-7.02 (1H, m, CH, **A**, 1H, m, CH, **B**), 6.93 (1H, d, *J* = 9.0 Hz, CH, **A**), 6.85 (1H, t, *J* = 7.6 Hz, CH, **A**), 6.65 (1H, t, *J* = 7.6 Hz, CH, **A**), 6.51 (1H, d, *J* = 7.6 Hz, CH, **A**), 6.16 (1H, s, CH, **B**), 6.03 (1H, s, CH, **A**), 4.13-4.04 (2H, m, CH_2, **B**), 4.00-3.77 (4H, m, CH_2, **A**), 3.45-3.38 (1H, m, CH_2, **B**), 3.36-3.30 (1H, m, CH_2, **B**), 2.86-2.62 (4H, m, CH_2, **A**, 4H, m, CH_2, **B**), 2.10-2.00 (1H, m, CH_2, **A**), 1.94-1.84 (1H, m, CH_2, **A**), 1,84-1.77 (2H, m, CH_2, **B**), 1.70-1.55 (2H, m, CH_2, **A**, 2H, m, CH_2, **B**), 1.19 (3H, t, *J* = 7.5 Hz, CH_3, **B**), 1.11 (3H, t, *J* = 7.7 Hz, CH_3, **A**), 0.87 (3H, t, *J* = 7.4 Hz, CH_3, **A**), 0.84 (3H, t, *J* = 7.2 Hz, CH_3, **B**). **Isomer A**: ^{13}C NMR (150 MHz, DMSO) δ 163.6, 160.0, 158.5, 158.1, 150.9, 138.6, 133.2, 128.6, 127.0, 125.7, 125.7, 122.9, 115.3, 112.6, 109.9, 109.2, 70.7, 42.9, 26.7, 24.5, 23.7, 22.3, 12.6, 10.7. **Isomer B**: ^{13}C NMR (150 MHz, DMSO) δ 163.4, 160.2, 158.7, 157.5, 150.5, 137.9, 130.3, 129.7, 127.0, 126.0, 125.0, 124.3, 115.1, 113.2, 110.3, 109.7, 70.7, 46.8, 26.8, 24.6, 23.8, 22.5, 12.8, 10.7. HRMS (m/z): $[M+H]^+$ calcd. for $C_{24}H_{26}NO_4$, 392.1856; found, 392.1856; Elem. Anal. Calcd for $C_{24}H_{25}NO_4$: C, 73.64; H, 6.44; N, 3.58. Found: C, 73.27; H, 6.54; N 3.55.

*7-Propoxy-4-propyl-8-(1,2,3,4-tetrahydroquinolin-1-carbonyl)-2H-chromen-2-one (**4c**):* The product was prepared according to General Method 5. Compound **2c** was used as starting material. Yield: 73% as a white solid; mp 122-123 °C as a dynamic equilibrium of *cis-trans* amide bond rotamers distinguishable by NMR in a ratio 2:1. ^{1}H NMR (500 MHz, DMSO) δ 8.06 (1H, d, *J* = 8.5 Hz, CH, **B**), 7.86 (1H, d, *J* = 8.5 Hz, CH, **B**), 7.71 (1H, d, *J* = 8.9 Hz, CH, **A**), 7.24-7.15 (3H, m, CH, **B**), 7.14-7.01 (1H, m, CH, **A**, 1H, m, CH, **B**), 6.97 (1H, d, *J* = 8.9 Hz, CH, **A**), 6.90 (1H, t, *J* = 7.5 Hz, CH, **A**), 6.69 (1H, t, *J* = 7.5 Hz, CH, **A**), 6.55 (1H, d, *J* = 7.5 Hz, CH, **A**), 6.21 (1H, s, CH, **B**), 6.08 (1H, s, CH, **A**), 4.13 (2H, t, *J* = 5.8 Hz, CH_2, **B**), 4.04-3.81 (4H, m, CH_2, **A**), 3.52-3.43 (1H, m, CH_2, **B**), 3.42-3.35 (1H, m, CH_2, **B**), 2.93-2.59 (4H, m, CH_2, **A**, 4H, m, CH_2, **B**), 2.15-2.06 (1H, m, CH_2, **A**), 2.00-1.91 (1H, m, CH_2, **A**), 1.91-1.84 (2H, m, CH_2, **B**), 1.75-1.47 (4H, m, CH_2, **A**, 4H, m, CH_2, **B**), 0.99 (3H, t, *J* = 7.5 Hz, CH_3, **B**), 0.92 (3H, t, *J* = 7.5 Hz, CH_3, **A**), 0.91 (3H, t, *J* = 7.2 Hz, CH_3, **A**), 0.91 (3H, t, *J* = 7.2 Hz, CH_3, **B**). **Isomer A**: ^{13}C NMR (125 MHz, DMSO) δ 163.3, 159.5, 157.8, 156.8, 150.6, 138.2, 132.9, 128.2, 126.9, 125.4, 125.3, 122.6, 115.0, 112.3, 110.5, 108.8, 70.3, 42.6, 32.8, 26.3, 23.3, 22.0, 21.3, 13.7, 10.3. **Isomer B:** ^{13}C NMR (125 MHz, DMSO) δ 163.1, 159.7, 157.2, 156.9, 150.2, 137.5, 129.9, 129.3, 126.9, 125.6, 124.7, 124.0, 114.8, 112.9, 110.9, 109.3, 70.4, 46.4, 33.0, 26.5, 23.5, 22.1, 21.5, 13.9, 10.3. HRMS (m/z): $[M+H]^+$ calcd. for $C_{25}H_{28}NO_4$, 406.2013; found, 406.2015; Elem. Anal. Calcd for $C_{25}H_{27}NO_4$: C, 74.05; H, 6.71; N, 3.45. Found: C, 74.24; H, 6.93; N 3.70.

*4-Butyl-7-propoxy-8-(1,2,3,4-tetrahydroquinolin-1-carbonyl)-2H-chromen-2-one (**4d**):* The product was prepared according to General Method 5. Compound **2d** was used as starting material. Yield: 59% as a white solid; mp 116-117 °C as a dynamic equilibrium of *cis-trans* amide bond rotamers distinguishable by NMR in a ratio 2.5:1. ^{1}H NMR (600 MHz, DMSO) δ 8.03 (1H, d, *J* = 9.0 Hz, CH, **B**), 7.82 (1H, d, *J* = 9.0 Hz, CH, **B**), 7.67 (1H, d, *J* = 8.7 Hz, CH, **A**), 7.21-7.12 (3H, m, CH, **B**), 7.11-7.03 (1H, m, CH, **A**, 1H, m, CH, **B**), 6.94 (1H, d, *J* = 8.7 Hz, CH, **A**), 6.87 (1H, t, *J* = 7.4 Hz, CH, **A**), 6.66 (1H, t, *J* = 7.4 Hz, CH, **A**), 6.51 (1H, d, *J* = 7.4 Hz, CH, **A**), 6.18 (1H, s, CH, **B**), 6.05 (1H, s, CH, **A**), 4.12-4.07 (2H, m, CH_2, **B**), 4.00-3.94 (1H, m, CH_2, **A**), 3.94-3.78 (3H, m, CH_2, **A**), 3.46-3.40 (1H, m, CH_2, **B**), 3.38-3.31 (1H, m, CH_2, **B**),

2.88-2.60 (4H, m, CH_2, **A**, 4H, m, CH_2, **B**), 2.11-2,03 (1H, m, CH_2, **A**,), 1.97-1.87 (1H, m, CH_2, **A**), 1,87-1.81 (2H, m, CH_2, **B**), 1.70-1.43 (4H, m, CH_2, **A**, 4H, m, CH_2, **B**), 1.41-1,35 (2H, m, CH_2, **B**), 1.35-1,26 (2H, m, CH_2, **A**), 0.90 (3H, t, $J = 7.5$ Hz, CH_3, **B**), 0.89 (3H, t, $J = 7.5$ Hz, CH_3, **A**), 0.89 (3H, t, $J = 7.5$ Hz, CH_3, **B**), 0.86 (3H, t, $J = 7.5$ Hz, CH_3, **A**). **Isomer A**: ^{13}C NMR (150 MHz, DMSO) δ 163.3, 159.5, 157.7, 157.0, 150.6, 138.2, 132.9, 128.2, 126.9, 125.3, 125.3, 122.6, 115.0, 112.2, 110.4, 108.8, 70.3, 42.6, 30.7, 30.2, 26.3, 23.3, 22.0, 22.0, 13.8, 10.3. **Isomer B**: ^{13}C NMR (150 MHz, DMSO) δ 163.1, 159.7, 157.2, 157.2, 150.2, 137.5, 129.9, 129.3, 126.9, 125.6, 124.7, 124.0, 114.8, 112.9, 110.8, 109.3, 70.3, 46.4, 30.9, 30.4, 26.4, 23.4, 22.1, 22.1, 13.9, 10.3. HRMS (m/z): $[M+H]^+$ calcd. for $C_{26}H_{30}NO_4$, 420.2169; found, 420.2176; Elem. Anal. Calcd for $C_{26}H_{29}NO_4$: C, 74.44; H, 6.97; N, 3.34. Found: C, 74.69; H, 6.97; N 3.27.

General Method 6. Synthesis of compounds 5a-d. 2-Bromoethan-1-ol (0.23 g, 0.13 mL, 1.8 mmol) was added to a solution of corresponding 4-alkyl-7-hydroxy-8-(1,2,3,4-tetrahydroquinoline-1-carbonyl)-2*H*-chromen-2-one **2a-d** (0.6 mmol) and potassium carbonate (0.25 g, 1.8 mmol) in DMF (7 mL). The reaction mixture was heated to 100 °C for 12 h. After cooling, the reaction mixture was diluted with EtOAc (50 mL), washed with water (3 × 30 mL) and brine (1 × 30 mL), organic layer was dried over anhydrous sodium sulfate, and concentrated under reduced pressure. The product was purified by column chromatography (mobile phase: hexane/EtOAc, 1:1; then hexane/EtOAc/acetic acid, 5:5:1).

*7-(2-Hydroxyethoxy)-4-methyl-8-(1,2,3,4-tetrahydroquinolin-1-carbonyl)-2H-chromen-2-one (**5a**):* The product was prepared according to General Method 6. Compound **2a** was used as starting material. Yield: 29% as a white solid; mp 171-172 °C as a dynamic equilibrium of *cis-trans* amide bond rotamers distinguishable by NMR in a ratio 2.5:1. ^1H NMR (500 MHz, DMSO) δ 8.09 (1H, d, $J = 8.6$ Hz, CH, **B**), 7.80 (1H, d, $J = 8.6$ Hz, CH, **B**), 7.65 (1H, d, $J = 8.8$ Hz, CH, **A**), 7.25-7.18 (3H, m, CH, **B**), 7.13-7.06 (1H, m, CH, **A**, 1H, m, CH, **B**), 7.03 (1H, d, $J = 8.8$ Hz, CH, **A**), 6.90 (1H, t, $J = 7.5$ Hz, CH, **A**), 6.70 (1H, t, $J = 7.5$ Hz, CH, **A**), 6.63 (1H, d, $J = 7.5$ Hz, CH, **A**), 6.27 (1H, s, CH, **B**), 6.14 (1H, s, CH, **A**), 4.23-4.18 (2H, m, CH_2, **B**), 4.13-4.06 (1H, m, CH_2, **A**), 4.02-3.92 (1H, m, CH_2, **A**, 1H, m, CH_2, **A**), 3.83-3.76 (1H, m, CH_2, **A**), 3.69 (2H, t, $J = 4.9$ Hz, CH_2, **B**), 3.64 (2H, t, $J = 4.9$ Hz, CH_2, **A**), 3.50-3.45 (2H, m, CH_2, **B**), 2.92-2.71 (2H, m, CH_2, **A**, 2H, m, CH_2, **B**), 2.44 (3H, s, CH_3, **B**), 2.33 (3H, s, CH_3, **A**), 2.17-2.07 (1H, m, CH_2, **A**,), 2.00-1.89 (1H, m, CH_2, **A**), 1.89-1.72 (2H, m, CH_2, **B**). **Isomer A**: ^{13}C NMR (125 MHz, DMSO) δ 163.3, 159.4, 158.1, 153.5, 150.3, 138.3, 133.0, 128.1, 127.1, 125.4, 125.4, 122.9, 114.9, 113.1, 111.5, 109.1, 70.8, 59.4, 42.6, 26.3, 23.4, 18.3. **Isomer B:** ^{13}C NMR (125 MHz, DMSO) δ 163.1, 159.6, 157.4, 153.6, 150.0, 137.6, 130.1, 129.3, 127.1, 125.6, 124.7, 124.2, 114.7, 113.8, 111.9, 109.6, 71.0, 59.6, 46.4, 26.5, 23.4, 18.4. HRMS (m/z): $[M+H]^+$ calcd. for $C_{22}H_{22}NO_5$, 380.1492; found, 380.1490; Elem. Anal. Calcd for $C_{22}H_{27}NO_8$ (trihydrate): C, 60.96; H, 6.28; N 3.23. Found: C, 60.82; H, 6.03; N 3.21.

*4-Ethyl-7-(2-hydroxyethoxy)-8-(1,2,3,4-tetrahydroquinolin-1-carbonyl)-2H-chromen-2-one (**5b**):* The product was prepared according to General Method 6. Compound **2b** was used as starting material. Yield: 60% as a white solid; mp 130-131 °C as a dynamic equilibrium of *cis-trans* amide bond rotamers distinguishable by NMR in a ratio 2.5:1. ^1H NMR (500 MHz, DMSO) δ 8.09 (1H, d, $J = 8.6$ Hz, CH, **B**), 7.85 (1H, d, $J = 8.6$ Hz, CH, **B**), 7.70 (1H, d, $J = 9.0$ Hz, CH, **A**), 7.25-7.18 (3H, m, CH, **B**), 7.14-7.06 (1H, m, CH, **A**, 1H, m, CH, **B**), 7.02 (1H, d, $J = 9.0$ Hz, CH, **A**), 6.90 (1H, dt, $J = 7.3$ Hz, $J = 1.0$ Hz, CH, **A**),

6.69 (1H, t, *J* = 7.3 Hz, CH, **A**), 6.63 (1H, d, *J* = 7.3 Hz, CH, **A**), 6.22 (1H, s, CH, **B**), 6.07 (1H, s, CH, **A**), 4.23-4.18 (2H, m, CH_2, **B**), 4.13-4.06 (1H, m, CH_2, **A**), 4.02-3.92 (1H, m, CH_2, **A**, 1H, m, CH_2, **A**), 3.83-3.76 (1H, m, CH_2, **A**), 3.70-3.67 (2H, m, CH_2, **B**), 3.64 (2H, t, *J* = 5.1 Hz, CH_2, **A**), 3.51-3.34 (2H, m, CH_2, **B**), 2.93-2.63 (4H, m, CH_2, **A**, 4H, m, CH_2, **B**), 2.18-2.05 (1H, m, CH_2, **A**), 2.02-1.88 (1H, m, CH_2, **A**), 1.88-1.79 (2H, m, CH_2, **B**), 1.24 (3H, t, *J* = 7.3 Hz, CH_3, **B**), 1.16 (3H, t, *J* = 7.3 Hz, CH_3, **A**). **Isomer A**: ^{13}C NMR (125 MHz, DMSO) δ 163.4, 159.7, 158.2, 158.0, 150.4, 138.3, 133.0, 128.2, 126.7, 125.5, 125.4, 122.9, 115.1, 112.3, 109.6, 109.2, 70.8, 59.4, 42.7, 26.3, 24.1, 23.4, 12.2. **Isomer B**: ^{13}C NMR (125 MHz, DMSO) δ 163.1, 159.9, 158.4, 157.3, 150.2, 137.6, 130.1, 129.3, 126.7, 125.6, 124.7, 124.2, 114.9, 113.0, 110.0, 109.6, 71.0, 59.6, 46.5, 26.5, 24.3, 23.4, 12.5. HRMS (m/z): $[M+H]^+$ calcd. for $C_{23}H_{24}NO_5$, 394.1649; found, 394.1660; Elem. Anal. Calcd for $C_{23}H_{23}NO_5$: C, 70.21; H, 5.89; N, 3.56. Found: C, 70.0; H, 6.2; N 3.55.

7-(2-Hydroxyethoxy)-4-propyl-8-(1,2,3,4-tetrahydroquinolin-1-carbonyl)-2H-chromen-2-one ***(5c)***: The product was prepared according to General Method 6. Compound **2c** was used as starting material. Yield: 82% as a white solid; mp 148-149 °C as a dynamic equilibrium of *cis-trans* amide bond rotamers distinguishable by NMR in a ratio 2.2:1. ^1H NMR (500 MHz, DMSO) δ 8.10 (1H, d, *J* = 8.8 Hz, CH, **B**), 7.86 (1H, d, *J* = 8.8 Hz, CH, **B**), 7.70 (1H, d, *J* = 9.0 Hz, CH, **A**), 7.24-7.18 (3H, m, CH, **B**), 7.13-7.06 (1H, m, CH, **A**, 1H, m, CH, **B**), 7.01 (1H, d, *J* = 9.0 Hz, CH, **A**), 6.90 (1H, dt, *J* = 7.6 Hz, *J* = 1.5 Hz, CH, **A**), 6.69 (1H, dt, *J* = 7.6 Hz, *J* = 1.5 Hz, CH, **A**), 6.62 (1H, d, *J* = 7.6 Hz, CH, **A**), 6.22 (1H, s, CH, **B**), 6.08 (1H, s, CH, **A**), 4.23-4.18 (2H, m, CH_2, **B**), 4.13-4.06 (1H, m, CH_2, **A**), 4.03-3.92 (1H, m, CH_2, **A**, 1H, m, CH_2, **A**), 3.82-3.75 (1H, m, CH_2, **A**), 3.71-3.67 (2H, m, CH_2, **B**), 3.65 (2H, t, *J* = 5.1 Hz, CH_2, **A**), 3.51-3.34 (2H, m, CH_2, **B**), 2.92-2.59 (4H, m, CH_2, **A**, 4H, m, CH_2, **B**), 2.18-2.06 (1H, m, CH_2, **A**), 2.02-1.89 (1H, m, CH_2, **A**), 1.89-1.80 (2H, m, CH_2, **B**), 1.71-1.49 (2H, m, CH_2, **A**, 2H, m, CH_2, **B**), 0.99 (3H, t, *J* = 7.3 Hz, CH_3, **B**), 0.91 (3H, t, *J* = 7.3 Hz, CH_3, **A**). **Isomer A**: ^{13}C NMR (125 MHz, DMSO) δ 163.4, 159.5, 158.0, 156.8, 150.5, 138.2, 133.0, 128.1, 126.9, 125.4, 125.3, 122.9, 115.1, 112.4, 110.5, 109.1, 70.8, 59.4, 42.7, 32.8, 26.3, 23.4, 21.3, 13.7. **Isomer B**: ^{13}C NMR (125 MHz, DMSO) δ 163.1, 159.8, 157.3, 156.9, 150.3, 137.6, 130.1, 129.3, 126.8, 125.6, 124.7, 124.2, 114.9, 113.0, 111.0, 109.6, 71.0, 59.6, 46.5, 33.0, 26.5, 23.4, 21.5, 13.9. HRMS (m/z): $[M+H]^+$ calcd. for $C_{24}H_{26}NO_5$, 408.1805; found, 408.1812; Elem. Anal. Calcd for $C_{24}H_{27}NO_6$ (hydrate): C, 67.75; H, 6.40; N, 3.29. Found: C, 68.13; H, 6.05; N 3.14.

4-Butyl-7-(2-hydroxyethoxy)-8-(1,2,3,4-tetrahydroquinolin-1-carbonyl)-2H-chromen-2-one ***(5d)***: The product was prepared according to General Method 6. Compound **2d** was used as starting material. Yield: 78% as a white solid; mp 114-115 °C as a dynamic equilibrium of *cis-trans* amide bond rotamers distinguishable by NMR in a ratio 2.2:1. ^1H NMR (500 MHz, DMSO) δ 8.09 (1H, d, *J* = 8.3 Hz, CH, **B**), 7.85 (1H, d, *J* = 8.3 Hz, CH, **B**), 7.70 (1H, d, *J* = 8.8 Hz, CH, **A**), 7.24-7.18 (3H, m, CH, **B**), 7.13-7.05 (1H, m, CH, **A**, 1H, m, CH, **B**), 7.02 (1H, d, *J* = 8.8 Hz, CH, **A**), 6.90 (1H, dt, *J* = 7.6 Hz, *J* = 1.0 Hz, CH, **A**), 6.69 (1H, dt, *J* = 7.6 Hz, *J* = 1.0 Hz, CH, **A**), 6.61 (1H, d, *J* = 7.6 Hz, CH, **A**), 6.22 (1H, s, CH, **B**), 6.08 (1H, s, CH, **A**), 4.23-4.19 (2H, m, CH_2, **B**), 4.13-4.06 (1H, m, CH_2, **A**), 4.03-3.92 (1H, m, CH_2, **A**, 1H, m, CH_2, **A**), 3.83-3.76 (1H, m, CH_2, **A**), 3.70-3.67 (2H, m, CH_2, **B**), 3.64 (2H, t, *J* = 5.2 Hz, CH_2, **A**), 3.51-3.36 (2H, m, CH_2, **B**), 2.92-2.61 (4H, m, CH_2, **A**, 4H, m, CH_2, **B**), 2.18-2.07 (1H, m, CH_2, **A**), 2.02-1.80 (1H, m, CH_2, **A**), 1.80-1.72 (2H, m, CH_2, **B**), 1.67-1.45 (2H, m, CH_2, **A**, 2H, m, CH_2, **B**), 1.45-1.37 (2H, m, CH_2, **B**), 1.37-1.28 (2H, m, CH_2, **A**), 0.93 (3H, t, *J* = 7.2 Hz, CH_3, **B**), 0.88 (3H, t, *J* = 7.2 Hz, CH_3, **A**).

Isomer A: ^{13}C NMR (125 MHz, DMSO) δ 163.4, 159.5, 158.0, 157.1, 150.5, 138.2, 133.0, 128.1, 126.9, 125.4, 125.3, 122.9, 115.1, 112.3, 110.4, 109.1, 70.8, 59.4, 42.6, 30.7, 30.2, 26.3, 23.4, 22.0, 13.9. **Isomer B**: ^{13}C NMR (125 MHz, DMSO) δ 163.1, 159.8, 157.3, 157.2, 150.3, 137.6, 130.1, 129.3, 126.8, 125.6, 124.7, 124.2, 114.9, 113.0, 110.9, 109.6, 71.0, 59.6, 46.5, 30.9, 30.4, 26.5, 23.4, 22.1, 13.9. HRMS (m/z): $[M+H]^+$ calcd. for $C_{25}H_{28}NO_5$, 422.1962; found, 422.1965; Elem. Anal. Calcd for $C_{25}H_{31}NO_7$ (dihydrate): C, 65.63; H, 6.83; N 3.06. Found: C, 65.25; H, 6.46; N, 3.21.

Scheme 4. Synthesis of compounds **6-8**.

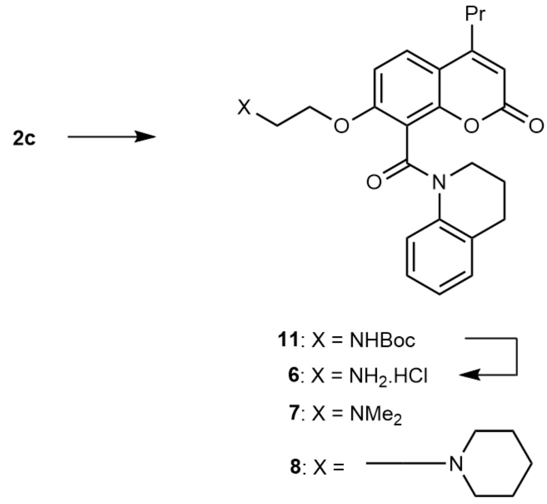

*Tert-Butyl (2-((2-oxo-4-propyl-8-(1,2,3,4-tetrahydroquinolin-1-carbonyl)-2H-chromen-7-yl)oxy)ethyl)carbamate (**11**):* *Tert*-Butyl (2-bromoethyl)carbamate (0.45 g, 2 mmol) was added to a suspension of compound **2c** (0.47 g, 1.3 mmol) and potassium carbonate (0.36 g, 2.6 mmol) in DMF (10 mL). The reaction mixture was heated to 130 °C for 24 h. After cooling, the reaction mixture was diluted with EtOAc (50 mL) and washed with water (3 × 70 mL) and brine (1 × 50 mL). The organic layer was dried over anhydrous sodium sulfate and concentrated under reduced pressure. Product **11** was purified by column chromatography (mobile phase: hexane/EtOAc, 1:1). Yield: 47% as a white solid; mp 73-74 °C as a dynamic equilibrium of *cis-trans* amide bond rotamers distinguishable by NMR in a ratio 2.4:1. ^{1}H NMR (500 MHz, DMSO) δ 8.07 (1H, d, J = 8.7 Hz, CH, **B**), 7.88 (1H, d, J = 8.7 Hz, CH, **B**), 7.72 (1H, d, J = 9.0 Hz, CH, **A**), 7.24-7.16 (3H, m, CH, **B**), 7.14-7.08 (1H, m, CH, **A**, 1H, m, CH, **B**), 7.01 (1H, d, J = 9.0 Hz, CH, **A**), 6.94-6.86 (1H, NH, **A**, 1H, NH, **B**), 6.90 (1H, td overlapped, J = 7.7 Hz, J = 1.2 Hz, CH, **A**), 6.69 (1H, td, J = 7.7 Hz, J = 1.2 Hz, CH, **A**), 6.56 (1H, dd, J = 7.7 Hz, J = 1.2 Hz, CH, **A**), 6.23 (1H, s, CH, **B**), 6.09 (1H, s, CH, **A**), 4.27-4.13 (2H, m, CH_2, **B**), 4.08-4.00 (1H, m, CH_2, **A**), 4.00-3.89 (2H, m, CH_2, **A**), 3.86-3.78 (1H, m, CH_2, **A**), 3.42 (2H, t, J = 6.5 Hz, CH_2, **B**), 3.30-3.25 (2H, m, CH_2, **B**), 3.23-3.17 (2H, m CH_2, **A**), 2.91-2.60 (4H, m CH_2, **A**, 4H, m CH_2, **B**), 2.16-2.25 (1H, m CH_2, **A**), 1.99-1.83 (1H, m CH_2, **A**, 2H, m CH_2, **B**), 1.71-1.50 (2H, m CH_2, **A**, 2H, m CH_2, **B**), 1.36 (9H, s, CH_3, **A**), 1.35 (9H, s, CH_3, **B**), 0.99 (3H, t, J = 7.3 Hz, CH_3, **B**), 0.91 (3H, t, J = 7.3 Hz, CH_3, **A**). **Isomer A:** ^{13}C NMR (125 MHz, DMSO) δ 163.2, 159.4, 157.5, 156.8, 155.8, 150.6, 138.2, 133.0, 128.1, 127.0, 125.4, 125.3, 122.8, 115.1, 112.5,

110.7, 108.9, 78.1, 67.5, 42.6, 39.2, 32.8, 28.3, 26.3, 23.4, 21.3, 13.7. **Isomer B:** ^{13}C NMR (125 MHz, DMSO) δ 162.9, 159.7 156.9, 156.8, 155.7, 150.3, 137.6, 130.0, 129.2, 126.9, 125.5, 124.7, 124.3, 114.9, 113.2, 111.1, 109.4, 78.0, 67.5, 46.4, 39.4, 33.0, 28.3, 26.5, 23.5, 21.5, 13.9. HRMS (m/z): $[M+H]^+$ calcd. for $C_{29}H_{35}N_2O_6$, 507.2490; found, 507.2498; Elem. Anal. Calcd for $C_{29}H_{34}N_2O_6$: C, 68.76; H, 6.77; N, 5.53. Found: C, 68.55; H, 6.74; N, 5.44.

*2-((2-Oxo-4-propyl-8-(1,2,3,4-tetrahydroquinoline-1-carbonyl)-2H-chromen-7-yl)oxy)ethan-1-amin hydrochloride (**6**):* Compound **11** (0.4 g, 0.8 mmol) was dissolved in acetic acid (5 mL), bubbled with hydrogen chloride and stirred at RT for 2 h. The reaction mixture was diluted with diethyl ether (20 mL) and concentrated under reduced pressure. The product was dried over P_2O_5 in a desiccator. Yield: 70% as a white solid; mp 129-130 °C as a dynamic equilibrium of *cis-trans* amide bond rotamers distinguishable by NMR in a ratio 2.7:1. ^{1}H NMR (500 MHz, DMSO) δ 8.34 (3H, bs, NH_3, **A**, 3H, bs, NH_3, **B**), 8.12 (1H, d, *J* = 8.6 Hz, CH, **B**), 7.91 (1H, d, *J* = 8.6 Hz, CH, **B**), 7.77 (1H, d, *J* = 9.0 Hz, CH, **A**), 7.28 (1H, d, *J* = 9.1 Hz, CH, **B**), 7.24-7.18 (2H, m, CH, **B**), 7.12 (1H, d, *J* = 9.0 Hz, CH, **A**), 7.11-7.07 (1H, m, CH, **A**, 1H, m, CH, **B**), 6.91 (1H, t, *J* = 7.7 Hz, CH, **A**), 6.70 (1H, t, *J* = 7.7 Hz, CH, **A**), 6.56 (1H, d, *J* = 7.7 Hz, CH, **A**), 6.26 (1H, s, CH, **B**), 6.08 (1H, s, CH, **A**), 4.43 (2H, d, *J* = 5.7 Hz, CH, **B**), 4.40-4.32 (1H, m, CH_2, **A**), 4.27-4.20 (1H, m, CH_2, **A**), 4.20-4.11 (1H, m, CH_2, **A**), 3.71-3.63 (1H, m, CH2, **A**), 3.51-3.41 (2H, m, CH_2, **B**), 3.20-3.08 (2H, m, CH_2, **A**, 2H, m, CH_2, **B**), 2.95-2.59 (4H, m, CH_2, **A**, 4H, m, CH_2, **B**), 2.23-2.12 (1H, m CH_2, **A**), 1.96-1.83 (1H, m, CH_2, **A**, 2H, m, CH_2, **B**), 1.72-1.48 (2H, m CH_2, **A**, 2H, m CH_2, **B**), 0.99 (3H, t, *J* = 7.3 Hz, CH_3, **B**), 0.90 (3H, t, *J* = 7.3 Hz, CH_3, **A**). **Isomer A**: ^{13}C NMR (125 MHz, DMSO) δ 163.2, 159.2 157.3, 156.6, 150.2, 138.1, 133.2, 128.1, 127.2, 125.5, 125.3, 123.1, 115.5, 113.1, 111.0, 109.9, 66.1, 42.8, 38.0, 32.8, 26.3, 23.4, 21.3, 13.7. **Isomer B**: ^{13}C NMR (125 MHz, DMSO) δ 162.8, 159.6 156.9, 156.4, 150.2, 137.6, 130.1, 129.3, 127.0, 125.6, 124.8, 124.3, 115.3, 113.7, 111.4, 110.2, 66.2, 46.5, 38.1, 33.0, 26.6, 23.4, 21.5, 13.9. HRMS (m/z): $[M+H]^+$ calcd. for $C_{24}H_{27}N_2O_4$, 407.1965; found, 407.1978; Elem. Anal. Calcd for $C_{24}H_{27}ClN_2O_4$: C, 65.08; H, 6.14; N, 6.32. Found: C, 64.88; H, 6.17; N, 6.24.

7-(2-(Dimethylamino)ethoxy)-4-propyl-8-(1,2,3,4-tetrahydroquinolin-1-carbonyl)-2H-chromen-2-one (7): 2-Chloro-*N,N*-dimethylethan-1-amine hydrochloride (0.18 g, 1.25 mmol) was added to a suspension of compound **2c** (0.3 g, 0.82 mmol) and potassium carbonate (0.23 g, 1.64 mmol) in DMF (10 mL). The reaction mixture was heated to 130 °C for 24 h. After cooling to RT, the reaction mixture was diluted with EtOAc (50 mL) and washed with water (3 × 50 mL) and brine (1 × 50 mL). The organic layer was dried over anhydrous sodium sulfate and concentrated under reduced pressure. Product **7** was suspended with diethyl ether (15 mL), filtered off and obtained in high quality without additional purification. Yield: 74% as a white solid; mp 128-129 °C as a dynamic equilibrium of *cis-trans* amide bond rotamers distinguishable by NMR in a ratio 2:1. ^{1}H NMR (500 MHz, DMSO) δ 8.06-8.03 (1H, m, CH, **B**), 7.86 (1H, d, *J* = 8.4 Hz, CH, **B**), 7.71 (1H, d, *J* = 9.3 Hz, CH, **A**), 7.23-7.18 (3H, m, CH, **B**), 7.13-7.06 (1H, m, CH, **A**, 1H, m, CH, **B**), 7.01 (1H, d, *J* = 9.1 Hz, CH, **A**), 6.90 (1H, dt, *J* = 7.7 Hz, *J* = 1.0 Hz, CH, **A**), 6.69 (1H, dt, *J* = 7.7 Hz, *J* = 1.0 Hz, CH, **A**), 6.59 (1H, dd, *J* = 7.7 Hz, *J* = 0.9 Hz, CH, **A**), 6.22 (1H, s, CH, **B**), 6.09 (1H, s, CH, **A**), 4.30-4.21 (2H, m, CH_2, **B**), 4.18-4.10 (1H, m, CH_2, **A**), 4.01-3.89 (1H, m, CH_2, **A**, 1H, m, CH_2, **A**), 3.88-3.81 (1H, m, CH_2, **A**), 3.50-3.30 (2H, m, CH_2, **B**), 2.91-2.57 (4H, m, CH_2, **A**, 6H, m, CH_2, **B**), 2.53-2.48 (2H, m, CH_2, **A**), 2.18 (6H, s, CH_3, **B**), 2.15 (6H, s, CH_3, **A**), 2.14-2.05 (1H, m, CH_2, **A**), 2.00-1.83 (1H, m, CH_2, **A**, 2H, m, CH_2, **B**), 1.71-1.50 (2H, m, CH_2, **A**, 2H, m, CH_2, **B**), 0.99 (3H, t, *J* = 7.4 Hz, CH_3, **B**), 0.92

(3H, t, J = 7.2 Hz, CH_3, **A**). **Isomer A**: ^{13}C NMR (125 MHz, DMSO) δ 163.3, 159.5, 157.6, 156.8, 150.6, 138.2, 132.9, 128.2, 126.9, 125.4, 125.3, 122.8, 115.0, 112.4, 110.6, 108.9, 67.2, 57.4, 45.8, 45.7, 32.8, 26.4, 23.3, 21.3, 13.7. **Isomer B**: ^{13}C NMR (125 MHz, DMSO) δ 163.1, 159.7, 157.0, 156.9, 150.2, 137.5, 130.0, 129.3, 126.9, 125.6, 124.7, 124.1, 114.8, 113.0, 111.0, 109.2, 67.8, 57.6, 46.5, 42.6, 33.0, 26.5, 23.4, 21.5, 13.9. HRMS (m/z): $[M+H]^+$ calcd. for $C_{26}H_{31}N_2O_4$, 435.2278; found, 435.2285; Elem. Anal. Calcd for $C_{26}H_{30}N_2O_4$: C, 71.87; H, 6.96; N, 6.45. Found: C, 71.92; H, 6.57; N, 6.18.

*7-(3-(Piperidin-1-yl)propoxy)-4-propyl-8-(1,2,3,4-tetrahydroquinolin-1-carbonyl)-2H-chromen-2-one (**8**):* 1-(3-Chloropropyl)piperidine hydrochloride (0.25 g, 1.26 mmol) was added to a suspension of compound **2c** (0.3 g, 0.82 mmol) and potassium carbonate (0.23 g, 1.64 mmol) in DMF (10 mL). The reaction mixture was heated to 130 °C for 24 h. After cooling to RT, the reaction mixture was diluted with EtOAc (50 mL) and washed with water (3 × 50 mL) and brine (1 × 50 mL). The organic layer was dried over anhydrous sodium sulfate and concentrated under reduced pressure. Product **8** was suspended with diethyl ether (15 mL), filtered off and obtained in high quality without additional purification. Yield: 98% as a white solid; mp 154-155 °C as a dynamic equilibrium of *cis-trans* amide bond rotamers distinguishable by NMR in a ratio 2:1. ^1H NMR (500 MHz, DMSO) δ 8.09-8.05 (1H, m, CH, **B**), 7.86 (1H, d, J = 9.0 Hz, CH, **B**), 7.71 (1H, d, J = 9.0 Hz, CH, **A**), 7.23-7.17 (3H, m, CH, **B**), 7.13-7.06 (1H, m, CH, **A**, 1H, m, CH, **B**), 6.98 (1H, d, J = 9.0 Hz, CH, **A**), 6.90 (1H, dt, J = 7.8 Hz, J = 1.4 Hz, CH, **A**), 6.69 (1H, t, J = 7.8 Hz, CH, **A**), 6.54 (1H, d, J = 7.8 Hz, CH, **A**), 6.22 (1H, s, CH, **B**), 6.09 (1H, s, CH, **A**), 4.19 (2H, t, J = 6.2, CH_2, **B**), 4.11-4.05 (1H, m, CH_2, **A**), 3.99-3.90 (1H, m, CH_2, **A**, 1H, m **A**), 3.88-3.81 (1H, m, CH_2, **A**), 3.51-3.44 (1H, m, CH_2, **B**), 3.40-3.31 (1H, m, CH_2, **B**), 2.93-2.59 (4H, m, CH_2, **A**, 4H, m, CH_2, **B**), 2.37-2.15 (6H, m, CH_2, **A**, 6H, m, CH_2, **B**), 2.15-2.05 (1H, m, CH_2, **A**), 2.01-1.91 (1H, m, CH_2, **A**), 1.91-1.51 (4H, m, CH_2, **A**, 6H, m, CH_2, **B**), 1.51-1.25 (6H, m, CH_2, **A**, 6H, m, CH_2, **B**), 0.99 (3H, t, J = 7.4 Hz, CH_3, **B**), 0.92 (3H, t, J = 7.2 Hz, CH_3, **A**). **Isomer A**: ^{13}C NMR (125 MHz, DMSO) δ 163.3, 159.5, 157.7, 156.8, 150.6, 138.2, 132.9, 128.2, 126.9, 125.4, 125.3, 122.7, 115.0, 112.3, 110.5, 108.9, 67.2, 54.7, 54.2, 42.6, 32.8, 26.4, 26.1, 25.8, 24.3, 23.4, 21.3, 13.7. **Isomer B**: ^{13}C NMR (125 MHz, DMSO) δ 163.1, 159.7, 157.2, 156.9, 150.2, 137.5, 129.9, 129.3, 126.9, 125.6, 124.7, 124.0, 114.8, 113.0, 110.9, 109.3, 67.3, 54.9, 54.2, 46.5, 33.0, 26.5, 26.3, 25.7, 24.2, 23.5, 21.5, 13.9. HRMS (m/z): $[M+H]^+$ calcd. for $C_{30}H_{37}N_2O_4$, 489.2748; found, 489.2750; Elem. Anal. Calcd for $C_{30}H_{36}N_2O_4$: C, 73.74; H, 7.43; N, 5.73. Found: C, 73.90; H, 7.40; N, 5.71.

Scheme 5. Synthesis of topobexin (**9**).

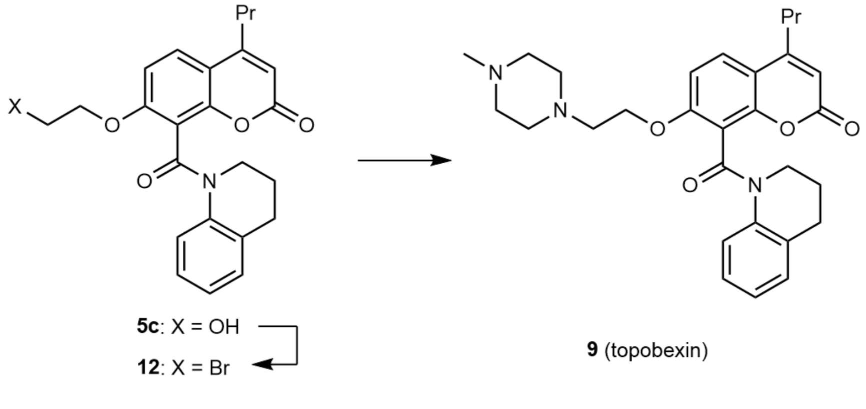

7-(2-Bromoethoxy)-4-propyl-8-(1,2,3,4-tetrahydroquinolin-1-carbonyl)-2H-chromen-2-one ***(12):*** Tetrabromomethane (0.53 g, 1.6 mmol) was added to a solution of compound **5c** (0.5 g, 1.23 mmol) and triphenylphosphine (0.42 g, 1.6 mmol) in acetonitrile (50 mL). The reaction mixture was stirred at RT for 48 h. Then, the reaction mixture was concentrated under reduced pressure. The product **12** was purified by column chromatography (mobile phase: hexane/EtOAc, 2:1). Yield: 52% as a white solid; mp 128-129 °C as a dynamic equilibrium of *cis-trans* amide bond rotamers distinguishable by NMR in a ratio 1.6:1. ^{1}H NMR (500 MHz, DMSO) δ 8.09 (1H, d, J = 8.5 Hz, CH, **B**), 7.87 (1H, d, J = 8.5 Hz, CH, **B**), 7.73 (1H, d, J = 9.0 Hz, CH, **A**), 7.24-7.18 (3H, m, CH, **B**), 7.13-7.07 (1H, m, CH, **A**, 1H, m, CH, **B**), 7.02 (1H, d, J = 9.0 Hz, CH, **A**), 6.90 (1H, td, J = 7.6 Hz, J = 1.3 Hz, CH, **A**), 6.72-6.67 (1H, m, CH, **A**), 6.65 (1H, dd, J = 7.6 Hz, J = 1.3 Hz CH, **A**), 6.24 (1H, s, CH, **B**), 6.10 (1H, s, CH, **A**), 4.59-4.27 (2H, m, CH_2, **A**, 2H, m, CH_2, **B**), 4.05-3.97 (1H, m, CH_2, **A**), 3.84-3.66 (3H, m, CH_2, **A**, 2H, m, CH_2, **B**), 3.49-3.42 (2H, m, CH_2, **B**), 2.93-2.59 (4H, m, CH_2, **A**, 4H, m, CH_2, **B**), 2.18-2.07 (1H, m CH_2, **A**), 2.02-1.81 (1H, m CH_2, **A**, 2H, m CH_2, **B**), 1.72-1.49 (2H, m CH_2, **A**, 2H, m CH_2, **B**), 0.99 (3H, t, J = 7.4 Hz, CH_3, **B**), 0.91 (3H, t, J = 7.3 Hz, CH_3, **A**). **Isomer A:** ^{13}C NMR (125 MHz, DMSO) δ 163.0, 159.4 157.0, 156.7, 150.5, 138.2, 132.9, 128.2, 126.9, 125.4, 125.3, 122.9, 115.2, 112.8, 110.8, 109.0, 69.0, 42.7, 32.8, 30.8, 26.3, 23.4, 21.3, 13.7. **Isomer B:** ^{13}C NMR (125 MHz, DMSO) δ 162.8, 159.7 156.9, 156.3, 150.2, 137.5, 130.1, 129.2, 126.1, 125.6, 124.7, 124.2, 115.0, 113.4, 111.2, 109.5, 69.0, 46.5, 33.0, 31.3, 26.5, 23.5, 21.5, 13.9. HRMS (m/z): $[M+H]^+$ calcd. for $C_{24}H_{25}BrNO_4$, 470.0961; found, 470.0970; Elem. Anal. Calcd for $C_{24}H_{24}BrNO_4$: C, 61.29; H, 5.14; N 2.98. Found: C, 61.47; H, 5.02; N 2.92.

*7-(2-(4-Methylpiperazin-1-yl)ethoxy)-4-propyl-8-(1,2,3,4-tetrahydroquinolin-1-carbonyl)-2H-chromen-2-one (**9**, topobexin):* 1-Methylpiperazine (0.18 g, 0.2 mL, 1.8 mmol) was added to a suspension of compound **12** (0.28 g, 0.6 mmol) and potassium carbonate (0.25 g, 1.8 mmol) in DMF (5 mL). The reaction mixture was heated to 60 °C for 5 h. After cooling, the reaction mixture was diluted with EtOAc (50 mL) and washed with water (3 × 70 mL) and brine (1 × 50 mL). The organic layer was dried over anhydrous sodium sulfate and concentrated under reduced pressure. Product **9** was suspended with diethyl ether (10 mL), filtered off and obtained in high quality without additional purification. Yield: 78% as a white solid; mp 45-46 °C as a dynamic equilibrium of *cis-trans* amide bond rotamers distinguishable by NMR in a ratio 1.7:1.

^{1}H NMR (500 MHz, DMSO) δ 8.09-8.00 (1H, m, CH, **B**), 7.86 (1H, d, J = 9.0 Hz, CH, **B**), 7.71 (1H, d, J = 9.0 Hz, CH, **A**), 7.23-7.17 (3H, m, CH, **B**), 7.13-7.07 (1H, m, CH, **A**, 1H, m, CH, **B**), 7.01 (1H, d, J = 9.0 Hz, CH, **A**), 6.90 (1H, td, J = 7.8 Hz, J = 1.3 Hz, CH, **A**), 6.69 (1H, td, J = 7.8 Hz, J = 1.3 Hz, CH, **A**), 6.59 (1H, dd, J = 7.8 Hz, J = 1.3 Hz, CH, **A**), 6.22 (1H, s, CH, **B**), 6.08 (1H, s, CH, **A**), 4.31-4.20 (2H, m, CH_2, **B**), 4.20-4.13 (1H, m, CH_2, **A**), 4.05-3.93 (2H, m, CH_2, **A**), 3.83-3.76 (1H, m, CH_2, **A**), 3.51-3.43 (1H, m, CH_2, **B**), 3.43-3.36 (1H, m, CH_2, **B**), 2.91-2.54 (6H, m, CH_2, **A**, 6H, m, CH_2, **B**), 2.48-2.34 (4H, m, CH_2, **A**, 4H, m, CH_2, **B**), 2.34-2.21 (4H, m, CH_2, **A**, 4H, m, CH_2, **B**), 2.11 (3H, s, CH_3, **A**), 2.06 (3H, s, CH_3, **B**), 2.00-1.82 (2H, m CH_2, **A**, 2H, m CH_2, **B**), 1.71-1.62 (2H, m CH_2, **B**), 1.62-1.50 (2H, m CH_2, **A**), 0.99 (3H, t, J = 7.3 Hz, CH_3, **B**), 0.91 (3H, t, J = 7.3 Hz, CH_3, **A**).

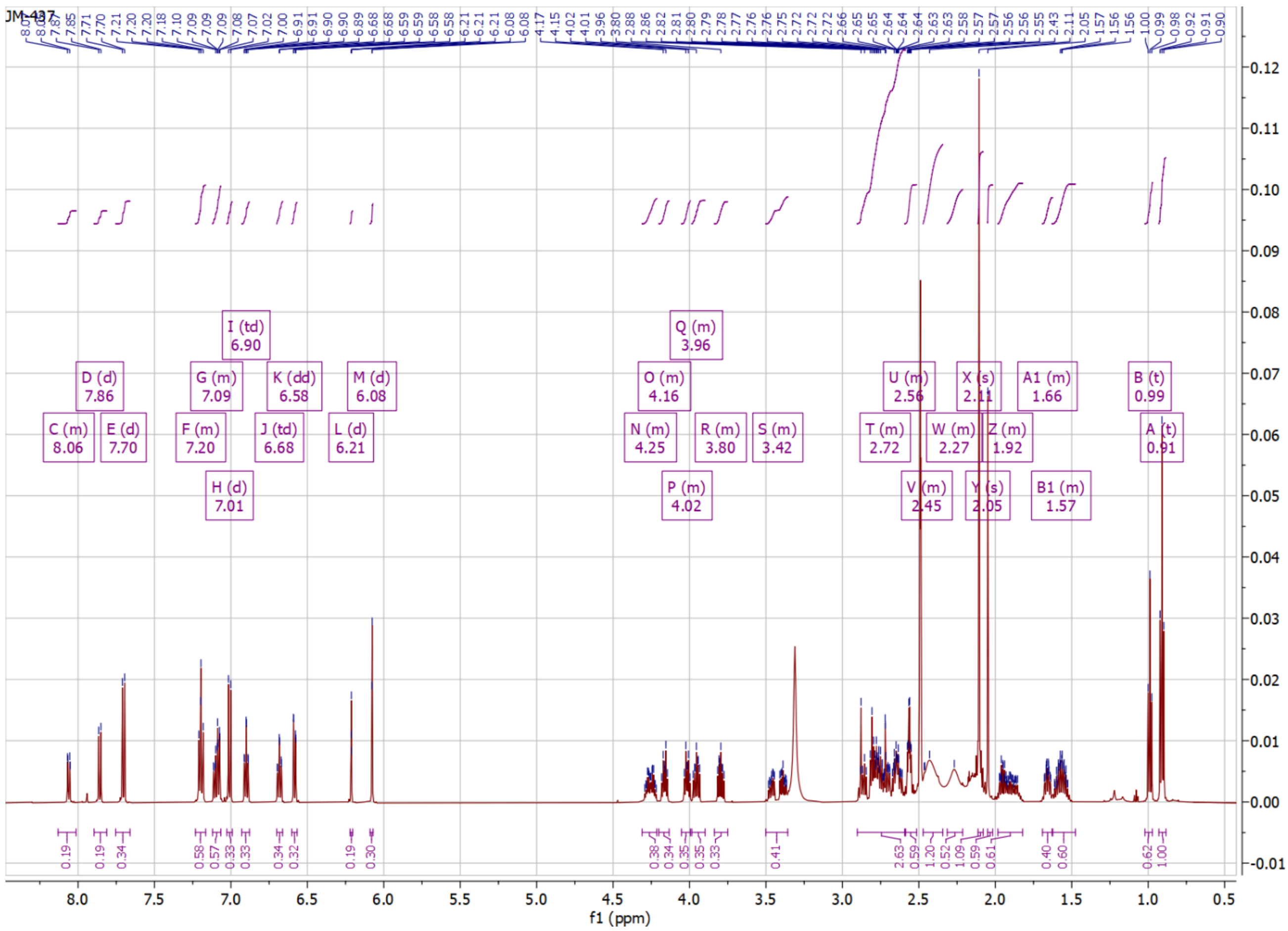

Isomer A: ^{13}C NMR (125 MHz, DMSO) δ 163.3, 159.5, 157.6, 156.8, 150.6, 138.2, 133.0, 128.2, 126.9, 125.4, 125.3, 122.8, 115.0, 112.4, 110.6, 109.0, 67.0, 56.3, 54.9, 53.2, 45.9, 42.6, 32.8, 26.4, 23.4, 21.3, 13.7. **Isomer B**: ^{13}C NMR (125 MHz, DMSO) δ 163.1, 159.7, 157.0, 156.9, 150.2, 137.5, 129.9, 129.3, 126.9, 125.6, 124.6, 124.0, 114.8, 113.0, 111.0, 109.2, 67.7, 56.5, 54.8, 53.2, 46.5, 45.9, 33.0, 26.6, 23.4, 21.5, 13.9.

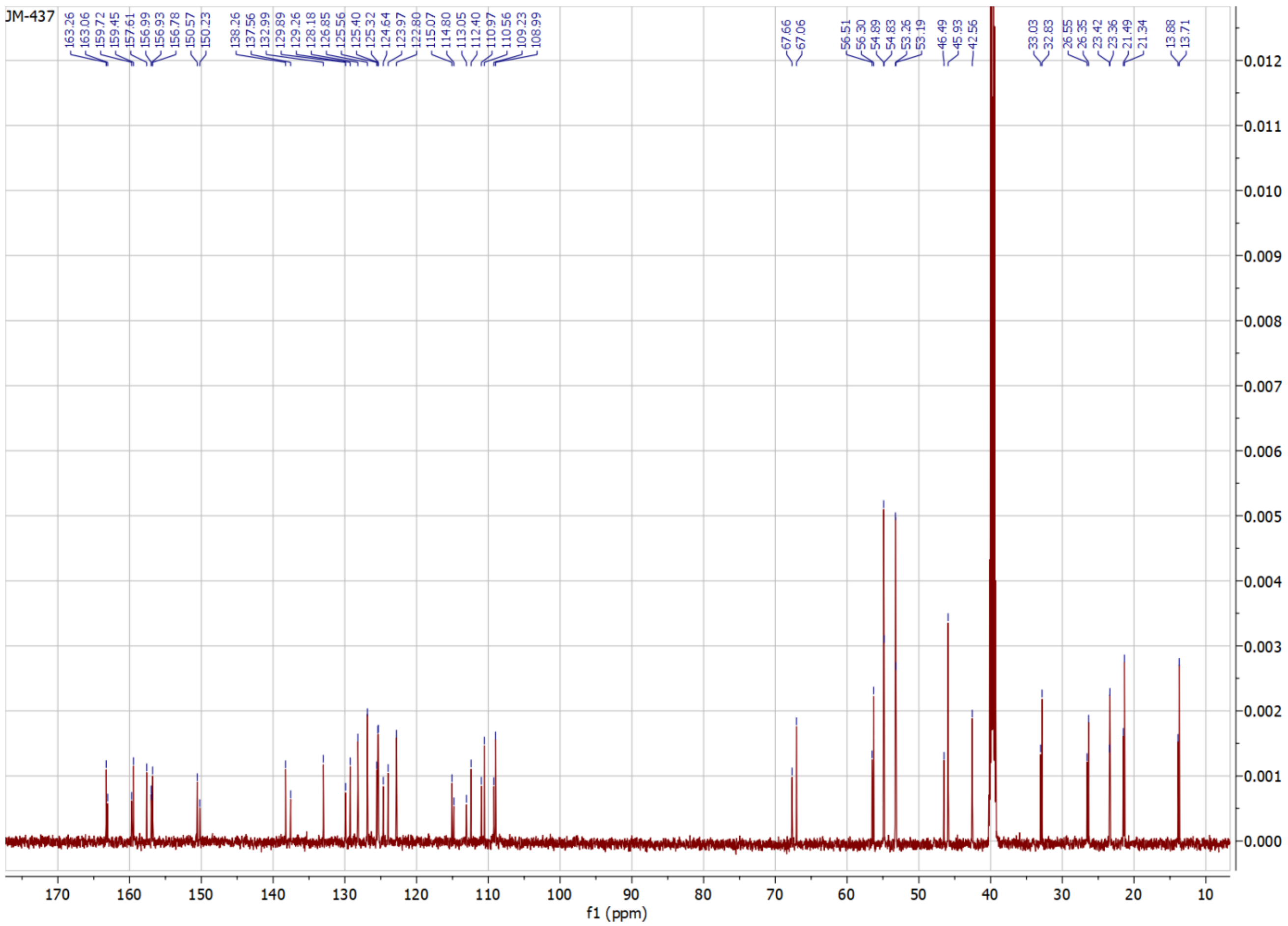

HRMS (m/z): $[M+H]^+$ calcd. for $C_{29}H_{36}N_3O_4$, 490.2700; found, 490.2708.

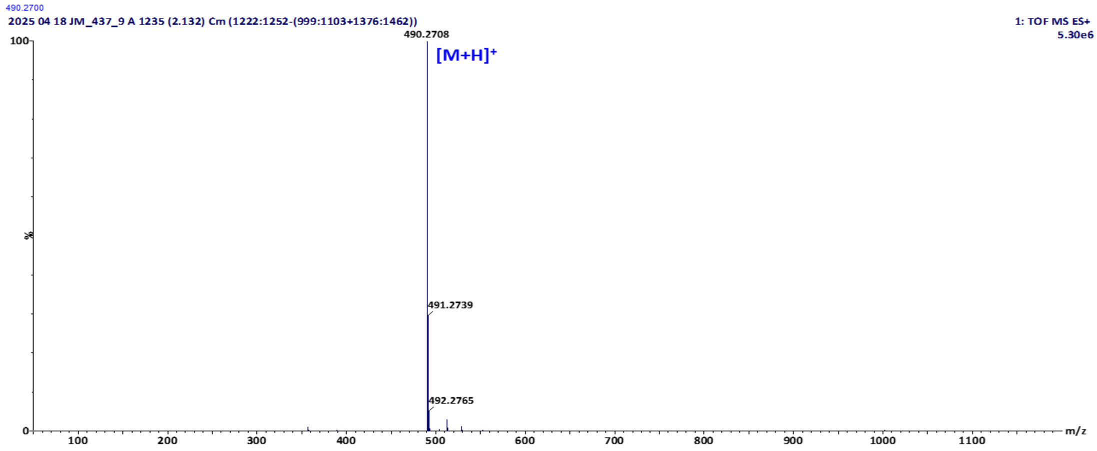

Elem. Anal. Calcd for $C_{29}H_{35}N_3O_4$: C, 71.14; H, 7.21; N 8.58. Found: C, 71.06; H, 7.32; N 8.50.

Scheme 6. Synthesis of compound **14**.

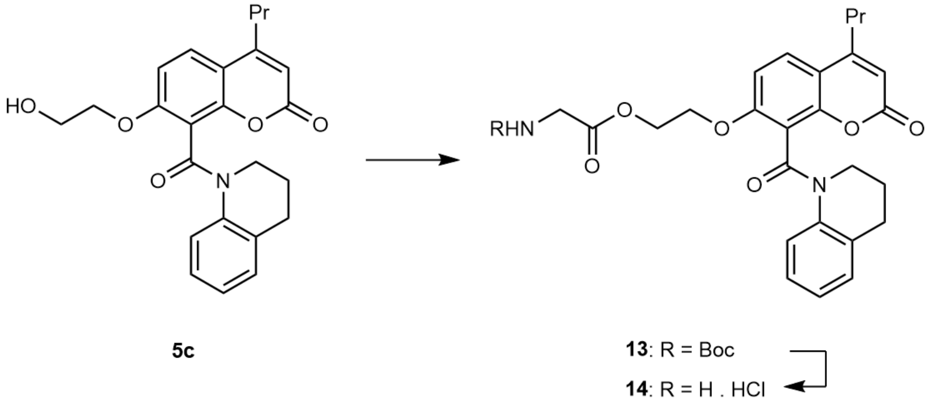

5c

13: R = Boc

14: R = H . HCl

2-((2-Oxo-4-propyl-8-(1,2,3,4-tetrahydroquinoline-1-carbonyl)-2H-chromen-7-yl)oxy)ethyl (tert-butoxycarbonyl)glycinate (13): EDC.HCl (1.41 g, 7.4 mmol) was added to the solution of *compound* **5c** (1.00 g, 2.4 mmol), (*tert*-butoxycarbonyl)glycine (1.29 g, 7.4 mmol) and DMAP (0.015 g, 0.1 mmol) in CH_2Cl_2 (15 mL). The reaction mixture was stirred under inert atmosphere at RT for 24 h. After completion, the reaction mixture was filtered off, filtrate was washed with 1 % aq. HCl (50 mL), water (50 mL), brine (50 mL) and water (50 mL). Organic layer was separated, dried over anhydrous sodium sulfate and evaporated under reduced pressure. The product was purified using column chromatography (mobile phase: hexane/EtOAc, 2:3). Yield: 87 % as a white solid; mp 94-95 °C as a dynamic equilibrium of *cis-trans* amide bond rotamers distinguishable by NMR in a ratio 2.3:1. ^{1}H NMR (600 MHz, DMSO-d_6) δ 8.02 (1H, d, J = 6.8 Hz, CH, **B**), 7.84 (1H, d, J = 7.5 Hz, CH, **B**), 7.69 (1H, d, J = 9.0 Hz, CH, **A**), 7.22-7.13 (3H, m, CH, **B**), 7.10-7.04 (1H, m, CH, **A**, 1H, m, CH, **B**), 6.99 (1H, d, J = 9.0 Hz, CH, **A**), 6.87 (1H, td, J = 7.3 Hz, J = 1.2 Hz, CH, **A**), 6.68 (1H, td, J = 7.3 Hz, J = 1.2 Hz, CH, **A**), 6.55 (1H, d, J = 7.3 Hz, CH, **A**), 6.20 (1H, s, CH, **B**), 6.07 (1H, s, CH, **A**), 4.44-4.32 (4H, m, CH_2, **B**), 4.32-4.21 (3H, m, CH_2, **A**), 4.19-4.12 (1H, m, CH_2, **A**), 3.97-3.89 (1H, m, CH_2, **A**), 3.84-3.75 (1H, m, CH_2, **A**), 3.64 (2H, d, J = 5.1 Hz, CH_2, **A**), 3.62-3.50 (2H, m, CH_2, **B**), 3.42-3.37 (2H, m, CH_2, **B**), 2.90-2.54 (4H, m, CH_2, **A**, 4H, m, CH_2, **B**), 2.13-2.03 (1H, m, CH_2, **A**), 1.97-1.79 (1H, m, CH_2, **A**, 2H, m, CH_2, **B**), 1.69-1.46 (2H, m, CH_2, **A**, 2H, m, CH_2, **B**), 1.34 (9H, s, CH_3, **A**), 1.32 (9H, s, CH_3, **B**), 0.96 (3H, t, J = 7.3 Hz, CH_3, **B**), 0.89 (3H, t, J = 7.3 Hz, CH_3, **A**). **Isomer A**: ^{13}C NMR (150 MHz, DMSO-d_6) δ 170.5, 163.1, 159.4, 157.2, 156.7, 155.9, 150.5, 138.2, 133.0, 128.2, 126.9, 125.4, 125.4, 122.7, 115.5, 112.7, 110.8, 109.1, 78.4, 67.1, 62.8, 42.6, 41.9, 32.8, 28.3, 26.2, 23.4, 21.3, 13.7. **Isomer B**: ^{13}C NMR (150 MHz, DMSO-d_6) δ 170.4, 162.8, 159.6, 156.8, 156.6, 155.9, 150.2, 137.5, 130.0, 129.3, 126.9, 125.6, 124.7, 124.1, 115.0, 113.4, 111.2, 109.5, 78.4, 67.2, 62.9, 46.4, 41.8, 33.0, 28.3, 26.4, 23.4, 21.5, 13.9. HRMS (m/z): $[M+H]^+$ calcd. for $C_{31}H_{37}N_2O_8$, 565.2544; found, 565.2548; Elem. Anal. Calcd. for $C_{31}H_{36}N_2O_8$: C, 65.94; H, 6.43; N, 4.96. Found: C, 65.76; H, 6.29; N, 4.62.

2-Oxo-2-(2-((2-oxo-4-propyl-8-(1,2,3,4-tetrahydroquinoline-1-carbonyl)-2H-chromen-7-yl)oxy)ethoxy)ethan-1-aminium chloride (14): Compound **13** (0.50 g, 0.9 mmol) was dissolved in acetic acid (5 mL), bubbled with hydrogen chloride for 5 min and the reaction mixture was stirred at RT for additional 2 h. Diethyl ester (25 mL) was added and the formed solid was filtered off and dried under reduced pressure. Yield: 81 % yield as a white solid; mp 119-120 °C as a dynamic equilibrium of *cis-trans*

amide bond rotamers distinguishable by NMR in a ratio 2.4:1. ^{1}H NMR (500 MHz, DMSO-d_6) δ 8.51 (3H, bs, $-(NH_3)^+$, **A**, 3H, bs, $-(NH_3)^+$, **B**), 8.06 (1H, d, J = 8.8 Hz, CH, **B**), 7.89 (1H, d, J = 8.8 Hz, CH, **B**), 7.74 (1H, d, J = 9.0 Hz, CH, **A**), 7.28-7.19 (3H, m, CH, **B**), 7.14-7.04 (1H, m, CH, **A**, 1H, m, CH, **B**), 7.06 (1H, d, J = 9.0 Hz, CH, **A**), 6.92 (1H, t, J = 7.4 Hz, CH, **A**), 6.72 (1H, t, J = 7.4 Hz, CH, **A**), 6.59 (1H, d, J = 7.4 Hz, CH, **A**), 6.24 (1H, s, CH, **B**), 6.10 (1H, s, CH, **A**), 4.51-4.32 (3H, m, CH_2, **A**, 4H, m, CH_2, **B**), 4.28-4.21 (1H, m, CH_2, **A**), 4.07-3.98 (1H, m, CH_2, **A**), 3.81-3.71 (1H, m, CH_2, **A**), 3.77 (2H, s, CH_2, **A**), 3.66 (2H, s, CH_2, **B**), 3.47-3.39 (2H, m, CH_2, **B**), 2.93-2.58 (4H, m, CH_2, **A**, 4H, m, CH_2, **B**), 2.20-2,07 (1H, m, CH_2, **A**), 1.98-1.85 (1H, m, CH_2, **A**, 2H, m, CH_2, **B**), 1.71-1.49 (2H, m, CH_2, **A**, 2H, m, CH_2, **B**), 0.99 (3H, t, J = 7.2 Hz, CH_3, **B**), 0.91 (3H, t, J = 7.5 Hz, CH_3, **A**). **Isomer A:** ^{13}C NMR (125 MHz, DMSO-d_6) δ 167.8, 163.2, 159.4, 157.3, 156.8, 150.4, 138.2, 133.1, 128.2, 127.0, 125.6, 125.4, 122.8, 115.3, 112.8, 110.8, 109.3, 67.0, 63.7, 42.7, 39.8, 32.8, 26.3, 23.4, 21.3, 13.7. **Isomer B:** ^{13}C NMR (125 MHz, DMSO-d_6) δ 167.8, 162.9, 159.7, 156.9, 156.5, 150.2, 137.5, 130.1, 129.4, 127.0, 125.7, 124.8, 124.1, 115.1, 113.5, 111.3, 109.7, 67.2, 63.9, 46.5, 39.7, 33.1, 26.5, 23.4, 21.5, 13.9. HRMS (m/z): $[M+H]^+$ calcd. for $C_{26}H_{29}N_2O_6$, 465.2020; found, 465.2032; Elem. Anal. Calcd. for $C_{26}H_{29}ClN_2O_6$: C, 62.34; H, 5.84; N, 5.59. Found: C, 62.02; H, 5.62; N, 5.29.
