# Supplementary material for: Topobexin targets the Topoisomerase II ATPase domain for beta isoform-selective inhibition and anthracycline cardioprotection
Source: Nat Commun. 2025 May 28;16:4928. doi: 10.1038/s41467-025-60167-9 (PMC12116762; doi:10.1038/s41467-025-60167-9)
Supplement: Supplementary file 2 — Reporting Summary [file 41467_2025_60167_MOESM2_ESM.pdf]

Corresponding author(s): Caroline A. Austin, Martin Štěrba, Tomáš Šimůnek, Jaroslav Roh, and Matthew J. Schellenberg

Last updated by author(s): Apr 16, 2025

Reporting Summary

Nature Portfolio wishes to improve the reproducibility of the work that we publish. This form provides structure for consistency and transparency in reporting. For further information on Nature Portfolio policies, see our Editorial Policies and the Editorial Policy Checklist.

Statistics

For all statistical analyses, confirm that the following items are present in the figure legend, table legend, main text, or Methods section.

n/a	Confirmed	
☐	☒	The exact sample size (*n*) for each experimental group/condition, given as a discrete number and unit of measurement
☐	☒	A statement on whether measurements were taken from distinct samples or whether the same sample was measured repeatedly
☐	☒	The statistical test(s) used AND whether they are one- or two-sided<br>*Only common tests should be described solely by name; describe more complex techniques in the Methods section.*
☒	☐	A description of all covariates tested
☐	☒	A description of any assumptions or corrections, such as tests of normality and adjustment for multiple comparisons
☐	☒	A full description of the statistical parameters including central tendency (e.g. means) or other basic estimates (e.g. regression coefficient) AND variation (e.g. standard deviation) or associated estimates of uncertainty (e.g. confidence intervals)
☐	☒	For null hypothesis testing, the test statistic (e.g. *F*, *t*, *r*) with confidence intervals, effect sizes, degrees of freedom and *P* value noted<br>*Give P values as exact values whenever suitable.*
☒	☐	For Bayesian analysis, information on the choice of priors and Markov chain Monte Carlo settings
☒	☐	For hierarchical and complex designs, identification of the appropriate level for tests and full reporting of outcomes
☒	☐	Estimates of effect sizes (e.g. Cohen's *d*, Pearson's *r*), indicating how they were calculated

Our web collection on statistics for biologists contains articles on many of the points above.

Software and code

Policy information about availability of computer code

Data collection	The following commercially available software were used: Zeiss Zen 3.6, Crysalis pro 1.171.42.71a, Image Lab 6.1.0, Tecan i-control 1.10.4.0, Magellan V7, Unicorn 7.3, Clariostar 5.61, LabChart 8, QuantStudio v 1.3, Fusion Solo 7S Edge 18.02, MassLynx 4.1.
Data analysis	The following commercially available software were used: Graphpad Prism 10, HKL2000 version 720, Phenix 1.17.1, Coot 0.89, Pymol 2.5.2, Fiji 1.0, Olex2 1.5, Crysalis pro 1.171.42.71a, Image Lab 6.1.0, MestReNova 15.0.1-38027, SigmaPlot 14.0, MassLynx 4.1.

For manuscripts utilizing custom algorithms or software that are central to the research but not yet described in published literature, software must be made available to editors and reviewers. We strongly encourage code deposition in a community repository (e.g. GitHub). See the Nature Portfolio guidelines for submitting code & software for further information.

Data

Policy information about availability of data

All manuscripts must include a data availability statement. This statement should provide the following information, where applicable:

- Accession codes, unique identifiers, or web links for publicly available datasets
- A description of any restrictions on data availability
- For clinical datasets or third party data, please ensure that the statement adheres to our policy

Cell lines and plasmids used in this study are made available through a material transfer agreement (MTA) upon request from the corresponding authors. Atomic coordinates and structure factors have been deposited in the PDB under accession numbers 9BQ6 [http://doi.org/10.2210/pdb9BQ6/pdb] (TOP2A ATPase), 9BQ7 [http://doi.org/10.2210/pdb9BQ7/pdb] (TOP2A ATPase + BNS-22), 9BQ9 [http://doi.org/10.2210/pdb9BQ9/pdb] (TOP2A ATPase + obex 5c), 9BQB [http://

doi.org/10.2210/pdb9BQB/pdb] (TOP2A ATPase + topobexin), 9BQ8 [http://doi.org/10.2210/pdb9BQ8/pdb] (TOP2B ATPase), 9BQA [http://doi.org/10.2210/pdb9BQA/pdb] (TOP2B ATPase + BNS-22), 9BQC [http://doi.org/10.2210/pdb9BQC/pdb] (TOP2B ATPase + obex 5c), and 9BQD [http://doi.org/10.2210/pdb9BQD/pdb] (TOP2B ATPase + topobexin). The X-ray crystallographic coordinates for small molecule structures reported in this study have been deposited at the Cambridge Crystallographic Data Centre (CCDC), under deposition numbers 2354347 (topobexin (9)) and 2354203 (BNS-22). These data can be obtained free of charge from The Cambridge Crystallographic Data Centre via www.ccdc.cam.ac.uk/data_request/cif.

Research involving human participants, their data, or biological material

Policy information about studies with human participants or human data. See also policy information about sex, gender (identity/presentation), and sexual orientation and race, ethnicity and racism.

Reporting on sex and gender	*Use the terms sex (biological attribute) and gender (shaped by social and cultural circumstances) carefully in order to avoid confusing both terms. Indicate if findings apply to only one sex or gender; describe whether sex and gender were considered in study design; whether sex and/or gender was determined based on self-reporting or assigned and methods used.* *Provide in the source data disaggregated sex and gender data, where this information has been collected, and if consent has been obtained for sharing of individual-level data; provide overall numbers in this Reporting Summary. Please state if this information has not been collected.* *Report sex- and gender-based analyses where performed, justify reasons for lack of sex- and gender-based analysis.*
Reporting on race, ethnicity, or other socially relevant groupings	*Please specify the socially constructed or socially relevant categorization variable(s) used in your manuscript and explain why they were used. Please note that such variables should not be used as proxies for other socially constructed/relevant variables (for example, race or ethnicity should not be used as a proxy for socioeconomic status).* *Provide clear definitions of the relevant terms used, how they were provided (by the participants/respondents, the researchers, or third parties), and the method(s) used to classify people into the different categories (e.g. self-report, census or administrative data, social media data, etc.)* *Please provide details about how you controlled for confounding variables in your analyses.*
Population characteristics	*Describe the covariate-relevant population characteristics of the human research participants (e.g. age, genotypic information, past and current diagnosis and treatment categories). If you filled out the behavioural & social sciences study design questions and have nothing to add here, write "See above."*
Recruitment	*Describe how participants were recruited. Outline any potential self-selection bias or other biases that may be present and how these are likely to impact results.*
Ethics oversight	*Identify the organization(s) that approved the study protocol.*

Note that full information on the approval of the study protocol must also be provided in the manuscript.

Field-specific reporting

Please select the one below that is the best fit for your research. If you are not sure, read the appropriate sections before making your selection.

[x] Life sciences [ ] Behavioural & social sciences [ ] Ecological, evolutionary & environmental sciences

For a reference copy of the document with all sections, see nature.com/documents/nr-reporting-summary-flat.pdf

Life sciences study design

All studies must disclose on these points even when the disclosure is negative.

Sample size	For biochemical or cellular assays a sufficient number of sample replicates (i.e. 3 or 4) were used to provide a sufficient estimate of error for individual data points. In animal experiments, there were 9-11 animals in each group in the chronic cardioprotective study and 6 animals were included in both the pharmacokinetic experiments and acute DNA damage experiment. The sample size was calculated using alpha set at 0.05, power 0.8-0.9 and the effect size was taken from historical or pilot experiments.
Data exclusions	A pre-determined criterion was applied in enzyme assays to exclude data showing obvious deviations from other technical replicates, or inconsistent readings that precluded slope measurement needed to calculate rates, which is often caused by air bubbles in assay wells. In animal experiments no exclusions were made and all collected data are shown.
Replication	Experiments were independently repeated at least twice and similar results were obtained.
Randomization	Animals were randomized into the experimental groups.
Blinding	Echocardiography was examined in the blinded manner, other analyses were perfomed in the opened manner.

Reporting for specific materials, systems and methods

We require information from authors about some types of materials, experimental systems and methods used in many studies. Here, indicate whether each material, system or method listed is relevant to your study. If you are not sure if a list item applies to your research, read the appropriate section before selecting a response.

Materials & experimental systems

n/a	Involved in the study
☐	☒ Antibodies
☐	☒ Eukaryotic cell lines
☒	☐ Palaeontology and archaeology
☐	☒ Animals and other organisms
☒	☐ Clinical data
☒	☐ Dual use research of concern
☒	☐ Plants

Methods

n/a	Involved in the study
☒	☐ ChIP-seq
☒	☐ Flow cytometry
☒	☐ MRI-based neuroimaging

Antibodies

Antibodies used	Anti-gamma H2A.X (phospho S139) antibody [9F3] (Abcam, product #ab26350, lots #GR3281249-2, #GR305763-6)<br>Anti-Mouse IgG (whole molecule)–Peroxidase antibody produced in rabbit (Sigma Aldrich, product A9044, lot #0000291320)<br>Horseradish peroxidase-conjugated goat anti-mouse immunoglobulin (DAKO, product # P044701)<br>Anti-Topoisomerase II alpha + Topoisomerase II beta/TOP2B antibody [EPR5377] (Abcam, product #ab109524, lot #GR42963-30)<br>Goat F(ab')2 Anti-Rabbit IgG F(ab')2 (HRP) preadsorbed (Abcam, product #ab6112, lot #GR3404091-8)<br>Anti-Topoisomerase 2 alpha antibody (Inspiralis HT2A21)<br>Anti-Topoisomerase 2 beta antibody (BD Biosciences 611493, Lot# 4205115)
Validation	All antibodies used in this study are commercially available and were validated by the supplier, with validation information available on the supplier's website

Eukaryotic cell lines

Policy information about cell lines and Sex and Gender in Research

Cell line source(s)	HEK293F cells were purchased commercially from Thermofisher Scientific, HL-60 cells were purchased commercially from ATCC
Authentication	HEK293F and HL-60 cells were authenticated by the manufacturer
Mycoplasma contamination	HEK293F cells were tested using Lonza Mycoalert mycoplasma detection kit, HL-60 cells were tested by PCR
Commonly misidentified lines (See ICLAC register)	*Name any commonly misidentified cell lines used in the study and provide a rationale for their use.*

Animals and other research organisms

Policy information about studies involving animals; ARRIVE guidelines recommended for reporting animal research, and Sex and Gender in Research

Laboratory animals	Species, strain, sex and age in animal studies are as reported in the Methods (adult, male New Zealand White rabbits, 12–16 weeks old; Velaz, Czech Republic). Wistar rats (Rattus norvegicus; 1- to 3-day-old) were used for isolation of cardiomyocytes.
Wild animals	The study did not involve wild animals.
Reporting on sex	Male animals were used as per well estabilished model. Male sex has been reported to be more prone to the toxicity developement and therefore it is often prefered to demonstrate cardioprotection in proof-of-concept studies. Neonatal cardiomyocytes were isolated from all rats in the litters without sex differentiation.
Field-collected samples	The study did not involve samples collected from the field.
Ethics oversight	The study on New Zealand White rabbits was approved by the Animal Welfare Committee of Charles University, Faculty of Medicine in Hradec Králové, and was carried out in the same institution in accordance with EU Directive 2010/63/EU.<br>The use of Wistar rats was approved by the Animal Welfare Committee of Charles University, Faculty of Pharmacy, and was carried out in the same institution in accordance with EU Directive 2010/63/EU.

Note that full information on the approval of the study protocol must also be provided in the manuscript.

Plants

Seed stocks	*Report on the source of all seed stocks or other plant material used. If applicable, state the seed stock centre and catalogue number. If plant specimens were collected from the field, describe the collection location, date and sampling procedures.*
Novel plant genotypes	*Describe the methods by which all novel plant genotypes were produced. This includes those generated by transgenic approaches, gene editing, chemical/radiation-based mutagenesis and hybridization. For transgenic lines, describe the transformation method, the number of independent lines analyzed and the generation upon which experiments were performed. For gene-edited lines, describe the editor used, the endogenous sequence targeted for editing, the targeting guide RNA sequence (if applicable) and how the editor was applied.*
Authentication	*Describe any authentication procedures for each seed stock used or novel genotype generated. Describe any experiments used to assess the effect of a mutation and, where applicable, how potential secondary effects (e.g. second site T-DNA insertions, mosiacism, off-target gene editing) were examined.*
